# Skeletal muscle properties in long COVID and ME/CFS differ from those induced by bed rest

**DOI:** 10.1038/s41467-026-75725-y

**Published:** 2026-07-28

**Authors:** Braeden T. Charlton, Anouk Slaghekke, Brent Appelman, Moritz Eggelbusch, Jelle Y. Huijts, Wendy Noort, Paul W. Hendrickse, Frank W. Bloemers, Jelle J. Posthuma, Paul van Amstel, Richie P. Goulding, Hans Degens, Richard T. Jaspers, Michèle van Vugt, Rob C. I. Wüst

**Affiliations:** 1 Department of Human Movement Sciences, Faculty of Behavioural and Movement Sciences, Amsterdam Movement Sciences, Vrije Universiteit Amsterdam, Amsterdam, Netherlands; 2 Center for Infection and Molecular Medicine, Amsterdam University Medical Centre, Amsterdam, Netherlands; 3 Amsterdam Institute of Infectious Disease, Amsterdam University Medical Centre, Amsterdam, Netherlands; 4 Exercise Biology Group, School of Medicine and Health, Technical University of Munich, Munich, Germany; 5 Faculty of Health and Medicine, Lancaster University, Lancaster, UK; 6 Department of Trauma Surgery, Amsterdam University Medical Centre, Amsterdam, Netherlands; 7 Department of Trauma Surgery, Flevoziekenhuis Almere, Almere, Netherlands; 8 Department of Pediatric Surgery, Amsterdam University Medical Centre, Amsterdam, Netherlands; 9 Department of Life Sciences, Manchester Metropolitan University, Manchester, UK; 10 Lithuanian Sports University, Institute of Sport Science and Innovations, Kaunas, Lithuania

**Keywords:** Energy metabolism, Infectious diseases, Metabolism

## Abstract

Patients with long COVID and myalgic encephalomyelitis/chronic fatigue syndrome (ME/CFS) suffer from post-exertional malaise. The accompanying physical inactivity may contribute to a lower aerobic capacity and may explain skeletal muscle adaptations in these patients. Here, we compare whole-body exercise responses and skeletal muscle adaptations after strict 60-day bed rest in healthy people with those in long COVID and ME/CFS patients, and healthy age- and sex-matched controls. Bed rest alters respiratory and cardiovascular responses to maximal exercise, which are dissimilar in patients. Bed rest causes muscle atrophy without altering fiber type. Both patient groups have more glycolytic fibers, and ME/CFS patients display type I-specific atrophy. Only after bed rest is oxidative phosphorylation capacity associated with maximal oxygen uptake. As skeletal muscle characteristics differ between patients and healthy individuals after bed rest, physical inactivity cannot solely explain the lower exercise capacity and skeletal muscle adaptations in long COVID and ME/CFS patients.

## Abstract (web summary)

Low aerobic capacity in patients with long COVID and ME/CFS is often attributed to physical inactivity. The authors show that long COVID and ME/CFS patients have distinct skeletal muscle changes that cannot be solely explained by physical inactivity.

## Introduction

Although most acute SARS-CoV-2 infections resolve within days to weeks, some symptoms persist or worsen in a subset of patients^1–3^. The continuation or development of symptoms after 12 weeks is termed long COVID, and patients typically experience debilitating fatigue, brain fog, postural orthostatic tachycardia, myalgia, and post-exertional malaise (PEM)^3^. PEM, experienced by ~90% of patients with long COVID^4^, is the worsening of symptoms following physical or cognitive exertion. While the pathophysiology underlying long COVID remains unclear, recent evidence suggests a close resemblance to myalgic encephalomyelitis/chronic fatigue syndrome (ME/CFS)^5^, another disease characterized by reduced exercise capacity, brain fog, and PEM. Importantly, PEM often results in avoidance of physical activity, which may contribute to the skeletal muscle abnormalities observed in both conditions^6–12^. Due to PEM, long COVID and ME/CFS patients may not achieve intensities or durations frequently enough to induce or maintain whole-body or skeletal muscle adaptations to exercise^13,14^. Depending on habitual activity levels, this may result in either maintenance of baseline muscle properties or, if activity is substantially reduced, progressive deconditioning over time^15^.

Deconditioning can range from mild step reductions to strict bed rest, with more severe models leading to more rapid reductions in aerobic capacity and skeletal muscle function^16–18^, including muscle atrophy, capillary rarefaction, as well as reduced mitochondrial content and mitochondrial function^16,19–21^. Cardiac output^20,22^ and maximal ventilatory capacity^23,24^ are also reduced after bed rest. However, whether these alterations cohere with those in patients with long COVID and ME/CFS has not been directly investigated. Patients with long COVID and ME/CFS exhibit lower aerobic exercise capacity and mitochondrial respiration^6,7,9,10^, reduced capillary densities^9^, muscle fiber atrophy^7^, and higher prevalences of atrophic fibers (small, triangular fibers)^6^, which has often been attributed to deconditioning^15,25^. However, whether deconditioning alone accounts for these changes remains unclear.

The current study aimed to compare whole-body exercise responses and skeletal muscle adaptations in patients with long COVID and ME/CFS to those observed following strict 60-day bed rest. We hypothesized that while whole-body aerobic capacity was similarly lower between patients and following bed rest, adaptations in skeletal muscle markers for oxygen supply and utilization diverged. As current literature regarding skeletal muscle alterations in long COVID patients often includes patients who have been hospitalized during acute infections, and skeletal muscle reports of patients with ME/CFS are scarce, we reasoned that non-hospitalized patients with long COVID and ME/CFS display no muscle atrophy whilst exhibiting lower capillary-to-fiber ratios and capillary densities. In contrast, we expected bed rest to result in severe muscle atrophy, reduced capillary-to-fiber ratios, and increased capillary density. Further, we expected muscle fiber size, capillarization, and mitochondrial markers to be associated with whole-body exercise capacity in healthy participants undergoing bed rest, but that such associations are lost in patients. To test these hypotheses, we analyzed aerobic exercise responses and vastus lateralis skeletal muscle biopsies of patients with ME/CFS and long COVID, and compared these to those of age- and sex-matched healthy controls (clinical trial NCT05225688), as well as a cohort of 24 healthy individuals who completed a strict bed rest for 60 days (clinical trial DRKS00015677).

## Results

### Participant characteristics

**Fig. 1:**
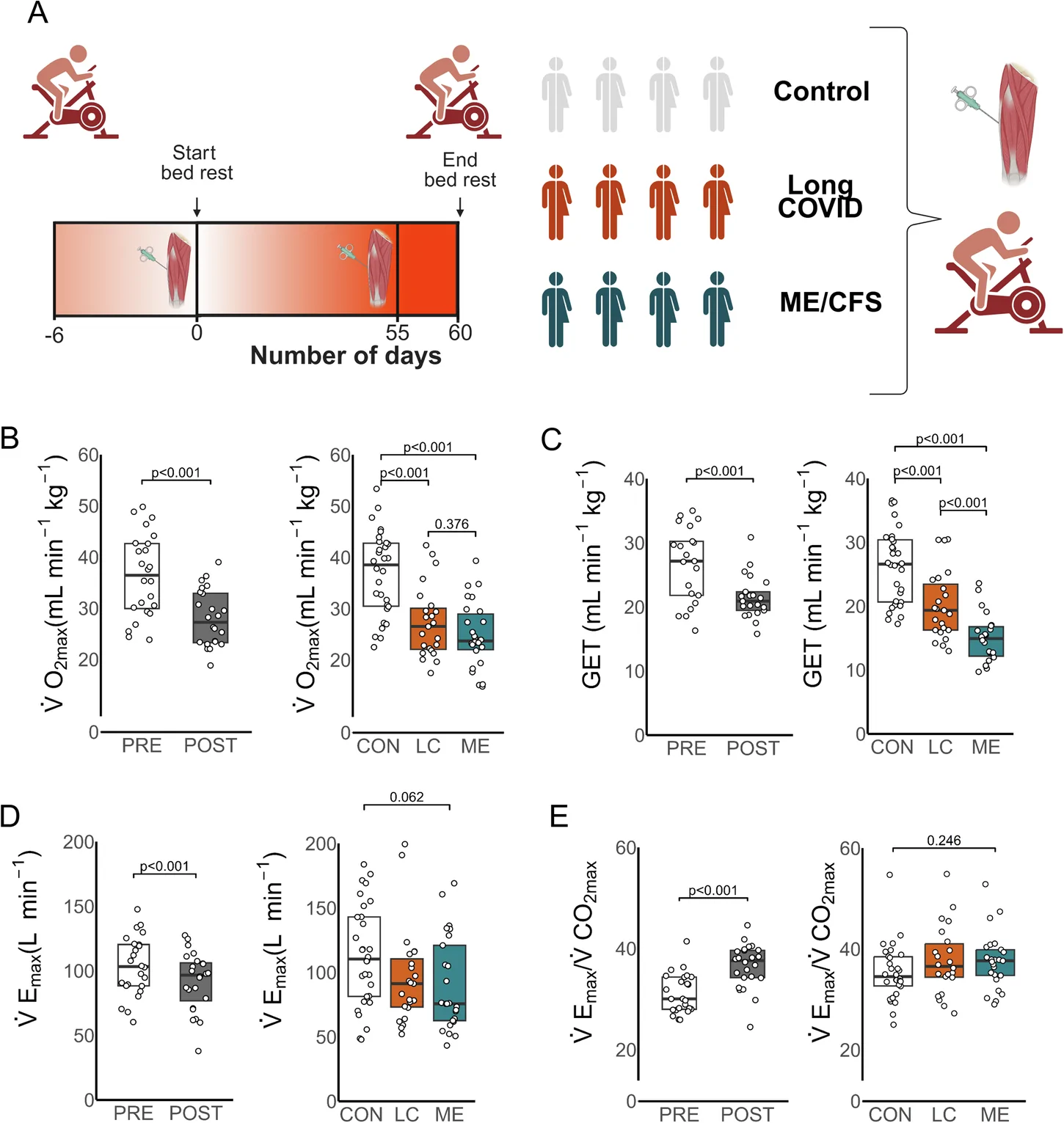
Bed rest results in alterations in respiratory and cardiovascular responses to maximal exercise, which are not seen in either patients with long COVID or ME/CFS. **A** Study designs for the bed rest and the myalgic encephalomyelitis/chronic fatigue syndrome (ME/CFS; ME), long COVID (LC), and healthy control (CON) cohorts. Bed rest decreased $\dot{V}{O}_{2\max }$ (**B**; *p* < 0.001, *n* = 24), gas exchange threshold (GET; **C**; *p* < 0.001, *n* = 21), and $\dot{V}{E}_{\max }$ (**D**; *p* < 0.001, *n* = 24), while $\dot{V}{E}_{\max }/\dot{V}C{O}_{2\max }$ increased (**E**; *p* < 0.001, *n* = 24). Patients with long COVID and ME/CFS had lower $\dot{V}{O}_{2\max }$ (**B**; *p* < 0.001, *n* = 23 LC, *p* < 0.001, *n* = 26 ME/CFS; *n* = 30 CON) and GET (**C**; *p* < 0.001; *n* = 23 LC; *p* < 0.001, *n* = 22 ME/CFS; *n* = 30 CON) compared to controls and $\dot{V}E$ tended to be lower (**D**; ANOVA: *p* = 0.062; *n* = 23 LC, *n* = 25 ME/CFS, *n* = 30 CON), however $\dot{V}{E}_{\max }/\dot{V}C{O}_{2\max }$ was not significantly different than controls (**E**; ANOVA: *p* = 0.246, *n* = 22 LC, *n* = 25 ME, *n* = 30 CON). $\dot{V}{O}_{2\max }$ and GET were non-normally distributed following Box-Cox transformations. Comparisons between pre- and post-bed rest were assessed using two-sided paired t-tests for parametric data and two-sided Mann–Whitney *U* tests for non-parametric data. Comparisons between patients with long COVID and patients with ME/CFS and healthy controls were assessed using analysis of variance, with Tukey HSD post-hoc testing for parametric data or Kruskal–Wallis *H* test, with pairwise Wilcoxon tests with Benjamini–Hochberg correction post-hoc for non-parametric data. Box plots display individual data points, the median (center line), and the first and third quartiles (lower and upper bounds of the box). Pre-bed rest and healthy controls are displayed in white, post-bed rest is displayed in gray, patients with long COVID are displayed in orange, and patients with ME/CFS are displayed in green. Created in BioRender. Charlton^84^ https://BioRender.com/gxz85by.

Table 1 provides the participant characteristics of the two cohorts, and Fig. 1A provides a graphical overview of the cohorts and study design. All healthy controls and 96.0% of patients with long COVID were vaccinated against SARS-CoV-2 at the time of measurements, while ME/CFS patients were diagnosed prior to 2019, and the bed rest study was conducted before the COVID-19 pandemic. Patients with long COVID developed symptoms prior to vaccination and met the Canadian Consensus Criteria for ME/CFS. Patients with ME/CFS were already diagnosed with ME/CFS (type of infection prior to diagnosis presented in Supplementary Fig. 1) before the COVID pandemic, and were therefore ill for a significantly longer time relative to long COVID patients (Table 1). Both patient groups displayed mild symptoms (DePaul Symptom Questionnaire Post-Exertional Malaise reported in Supplementary Fig. 2 and symptoms in Supplementary Fig. 3), as is implied by their willingness and ability to undergo exercise testing. Patients with long COVID and ME/CFS displayed large interindividual differences in daily step count (range: 733-8609 steps/day; Table 1).

**Table 1:** “Cohort characteristics

	Bed rest		Control	Long COVID	ME/CFS
	**Pre**	**Post**			
Participants (# Females)	24 (8)	24 (8)	30 (15)	25 (13)	26 (13)
Age (years)	30 (27–38)	30 (27–37)	42 (33–54) §	43 (31–50) §	42 (32–50) §
Height (cm)	175 (172–181)	175 (170–181)	179 (174–186)	177 (171–184)	176 (171–186)
Weight (kg)	74 (70–79)	74 (69–79)	76 (64–88)	80 (69–92)	84 (75–89)
Symptom Duration (days)	n/a	n/a	n/a	545 (455–686) †††	3793 (2510–5496)
Daily Steps (steps*day^-1^)	ND	0 (0–0)	7153 (5063–8405) †††	4718 (2794–5832) ***	3704 (2286–5052) ***

Symptom duration is reported from symptom onset to the day of study enrollment (February 2022 for long COVID and October 2023 for ME/CFS).

*: *vs*. control; †: *vs*. ME/CFS; §: *vs*. Bed Rest */†/§: *p* < 0.05; **/††/§§: *p* < 0.01; ***/†††/§§§: *p* < 0.001.

Data presented as median (IQR), as the data was non-normally distributed.

Healthy bed rest participants were, on average, younger than healthy controls matched to patients (*p* = 0.02). In a subgroup analysis, we age- and sex-matched pre-bed rest participants to patient-matched healthy controls. Maximal oxygen uptake ($\dot{V}{O}_{2\max }$, Supplementary Fig. 4A) relative to weight was not different, however patient-matched healthy controls had higher $\dot{V}{O}_{2\max }$ compared to the pre-bed rest participants when expressed relative to age- and sex-predicted values (Supplementary Fig. 4B). The gas exchange threshold (GET; Supplementary Fig. 4C, D) did not differ between cohorts. Fiber cross-sectional area (FCSA; Supplementary Fig. 4E) was also similar between groups; however, the pre-bed rest cohort exhibited lower capillary-to-fiber ratios (Supplementary Fig. 4F), lower succinate dehydrogenase (SDH) activity (Supplementary Fig. 4G), and a tendency to have a lower oxidative phosphorylation capacity (*p* = 0.092, Supplementary Fig. 4H). As some skeletal muscle features differed between those control groups, we analyzed them separately: healthy controls were matched to patients with ME/CFS and long COVID (Table 1), whereas the long-term bed rest cohort was analyzed longitudinally.

### Acute exercise responses

Bed rest is known to reduce maximal aerobic exercise capacity, and we expected both patient groups to exhibit lower maximal aerobic exercise capacities compared to healthy controls. Indeed, $\dot{V}{O}_{2\max }$ (Fig. 1B) and peak power output (Supplementary Fig. 5A) were reduced after 60-day bed rest, and were similarly lower in patients with long COVID and ME/CFS compared to healthy controls (Fig. 1B + Supplementary Fig. 5A). The same trends were observed when $\dot{V}{O}_{2\max }$ was expressed as a percentage of age- and sex-predicted values (Supplementary Fig. 6).

The GET, a submaximal non-invasive marker for the lactate threshold, was reduced following bed rest (Fig. 1C), and was similarly lower in both patient groups compared to healthy controls (Fig. 1C). Following bed rest, GET occurred at a higher percentage of $\dot{V}{O}_{2\max }$ (Supplementary Fig. 5B), whereas in patients with ME/CFS, GET occurred at a lower percentage of $\dot{V}{O}_{2\max }$ compared to both patients with long COVID and healthy controls (Supplementary Fig. 5B), suggesting an earlier relative onset of lactate accumulation in patients.

Bed rest also altered ventilatory and cardiovascular responses to exercise. Maximal minute ventilation was reduced ($\dot{V}{E}_{\max }$; Fig. 1D), whereas ventilatory equivalents for O_2_ and CO_2_ ($\dot{V}{E}_{\max }$/$\dot{V}C{O}_{2\max }$ and $\dot{V}{E}_{\max }$/$\dot{V}{O}_{2\max }$), and the $\dot{V}E$/$\dot{V}C{O}_{2}$ slope (Fig. 1E + Supplementary Fig. 5C-D) increased after 60 days of strict bed rest. Cardiovascular alterations included a reduced maximal O_2_-pulse (i.e., $\dot{V}{O}_{2\max }$/maximal heart rate, equal to the product of stroke volume and arteriovenous O_2_ difference; Supplementary Fig. 5E); increased maximal heart rate (Supplementary Fig. 4F), and increased adjusted heart rate reserve (AHRR; Supplementary Fig. 5G).

While $\dot{V}{E}_{\max }$ (Fig. 1D) tended to be lower in patients compared to healthy controls (*p* = 0.062), there were no significant differences in $\dot{V}{E}_{\max }$/$\dot{V}C{O}_{2\max }$, $\dot{V}{E}_{\max }$/$\dot{V}{O}_{2\max }$, the $\dot{V}E$/$\dot{V}C{O}_{2}$ slope, maximal heart rate, or AHRR (Fig. 1E, Supplementary Fig. 5C + D + F + G). However, both patients with long COVID and ME/CFS exhibited a lower O_2_-pulse compared to healthy controls (Supplementary Fig. 5E), similar to the effects of bed rest. Patients with ME/CFS had a more pronounced increase in heart rate relative to oxygen uptake (HR-$\dot{V}{O}_{2}$ slope; Supplementary Fig. 5H) compared to healthy controls, a pattern not seen following bed rest. These results are suggestive of either a lower stroke volume and/or an impaired peripheral oxygen extraction in patients.

Whole-body exercise results suggest that altered ventilatory patterns occurred concomitantly with the reduction in $\dot{V}{O}_{2\max }$ following bed rest, whilst ventilatory patterns in patients remain largely similar to those of healthy controls. Rather, patients indicate reductions in O_2_-pulse and elevations in HR-$\dot{V}{O}_{2}$ slope, which are suggestive of a lower stroke volume or peripheral oxygen extraction.

### Skeletal muscle fiber-type alterations

**Fig. 2:**
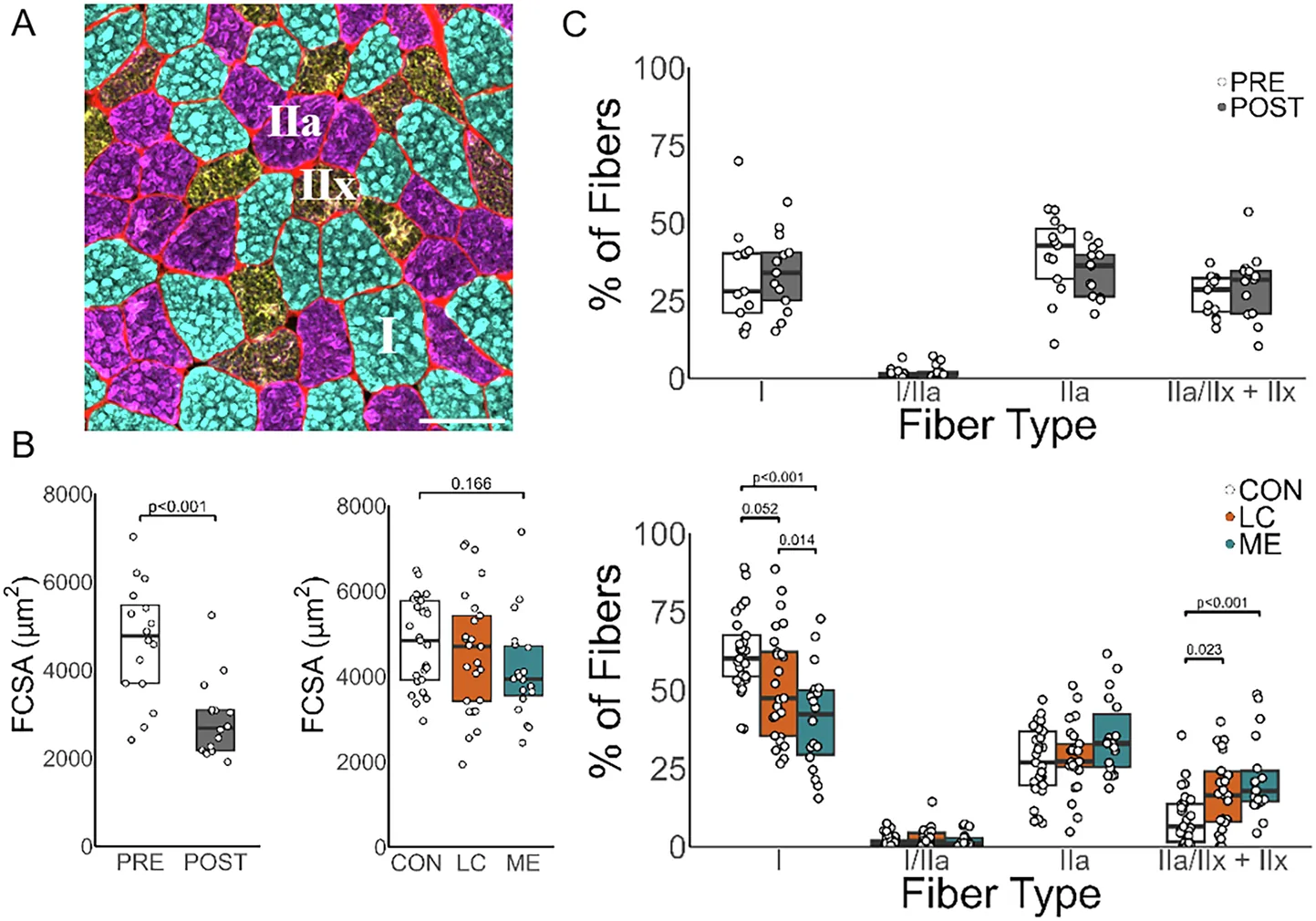
Bed rest induces muscle atrophy, with no change to fiber-type composition. Both patients with long COVID and ME/CFS have more glycolytic fibers than healthy, matched controls. **A** A typical example of muscle fiber-type staining with colors adjusted to be color-blind visible. The scale bar represents 100 µm. Severe fiber atrophy was observed following bed rest (**B**; *p* < 0.001, *n* = 16), whereas fiber cross-sectional area (FCSA) was not different between patients with long COVID (LC, *n* = 25) and myalgic encephalomyelitis/chronic fatigue syndrome (ME/CFS; ME, *n* = 19) and healthy controls (**B**; ANOVA: *p* = 0.174, *n* = 29 CON). Following bed rest, fiber-type composition was not significantly altered (**C**; Linear mixed model: *p* = 0.877, *n* = 16). Fiber-type composition was significantly different between patients with long COVID, patients with ME/CFS, and healthy controls (**C**; Linear mixed model: *p* = 0.004, *n* = 25 long COVID, *n* = 19 ME/CFS, *n* = 29 healthy controls). Long COVID (*p* = 0.052, *n* = 25) and ME/CFS (*p* < 0.001, *n* = 19) patients exhibited lower proportions of type I fibers compared to healthy controls (*n* = 29, albeit *p* = 0.052 for long COVID), while both patients with long COVID (*p* = 0.023, *n* = 25) and ME/CFS (*p* < 0.001, *n* = 19) had significantly higher proportions of type IIa/IIx+IIx fibers compared to healthy controls (**C**; *n* = 29 CON). All data was normally distributed following Box-Cox transformations, except proportions of IIa/IIx+IIx fibers. Fiber-type proportions were assessed using a linear mixed random effects model using % of proportion as the dependent variable and group x fiber type as the fixed effects, with subject ID as the random effects variable, and post-hoc testing carried out per fiber type as described below. Two-sided paired t-tests were used to assess parametric data from the bed rest cohort. Non-parametric data from the bed rest cohort was assessed using two-sided Mann–Whitney *U* tests. Analysis of variance, with Tukey HSD post-hoc testing or Kruskal–Wallis *H* test, with pairwise Wilcoxon tests with Benjamini–Hochberg correction post-hoc, was used to assess parametric and non-parametric data for patient cohorts. Box plots display individual data points, the median (center line), and the first and third quartiles (lower and upper bounds of the box). Pre-bed rest and healthy controls are displayed in white, post-bed rest is displayed in gray, patients with long COVID are displayed in orange, and patients with ME/CFS are displayed in green.

As muscle unloading can induce atrophy within days^19^, we assessed muscle fiber cross-sectional area (FCSA), fiber-type-specific FCSA, and fiber-type composition using immunohistochemistry (Fig. 2A). As expected, bed rest induced muscle atrophy (Fig. 2B), affecting all fiber types similarly (Supplementary Fig. 7A). In contrast, overall muscle FCSA of patients with long COVID and ME/CFS did not differ from healthy controls (Fig. 2B). However, when assessing fiber-type-specific FCSA, patients with ME/CFS exhibited significantly smaller type I fibers compared to healthy controls (Supplementary Fig. 7B), while all other fiber-type sizes remained similar to those of healthy controls. This suggests selective atrophy of type I fibers in patients with ME/CFS, but not after long-term bed rest.

Following long-term bed rest, fiber-type composition did not change (Fig. 2C and Supplementary Fig. 7C). In contrast, patients with long COVID and ME/CFS had a lower proportion of type I fibers (albeit *p* = 0.052 for long COVID) and a greater proportion of type IIa/IIx + IIx fibers compared to healthy controls (Fig. 2C), while patients with ME/CFS had an even lower proportion of type I fibers than patients with long COVID. We observed similar results when expressing fiber-type distribution as numerical percentages (Supplementary Fig. 7D). This indicates either a greater propensity for those with greater proportions of type IIa/IIx + IIx fibers to contract chronic illness or suggests a fiber-type shift occurring in patients. As we noted a selective type I atrophy in patients, these results suggest a fiber-type shift away from type I fibers toward more type II fiber types. The muscle atrophy was not fiber-type-specific after long-term bed rest, and bed rest did not induce a similar fiber-type shift.

### Mitochondrial respiration and activity

**Fig. 3:**
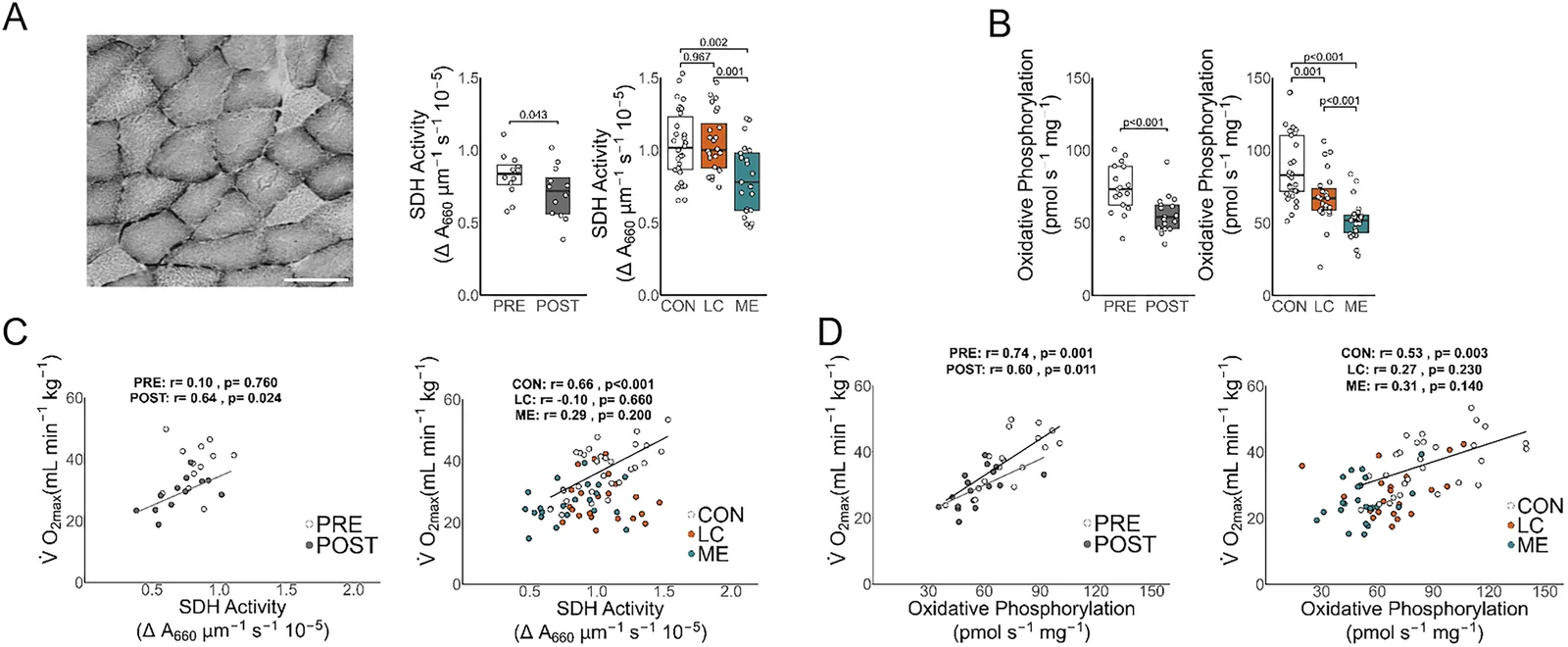
Bed rest decreases both SDH activity and oxidative phosphorylation capacity, which relate to maximal oxygen uptake. Lower SDH activity and oxidative phosphorylation capacity in patients with long COVID and ME/CFS do not relate to maximal oxygen uptake. Succinate dehydrogenase (SDH) activity (**A**) was significantly reduced following bed rest (*p* = 0.043, *n* = 12). SDH activity in patients with long COVID (LC, *n* = 24) was not different from healthy controls (CON; *p* = 0.967, *n* = 30), but lower in myalgic encephalomyelitis/chronic fatigue syndrome (ME/CFS; ME, *n* = 21, *p* = 0.002; **A**). Oxidative phosphorylation capacity (**B**) was reduced following bed rest (*p* < 0.001, *n* = 17) and was lower in both patients with long COVID (*p* = 0.001, *n* = 23) and ME/CFS (*p* < 0.001, *n* = 23) compared to healthy controls (*n* = 30). SDH was associated with$\,\dot{V}{O}_{2\max }$ (**C**) after bed rest (*r* = 0.65, *p* = 0.024, *n* = 12) and in healthy controls (*r* = 0.66, *p* < 0.001, *n* = 30). The relationship between oxidative phosphorylation capacity and $\dot{V}{O}_{2\max }$ (**D**) was significant in pre- and post-bed rest (*r* = 0.74, *p* < 0.001, *n* = 17; *r* = 0.60, *p* = 0.0113, *n* = 17) and healthy controls (*r* = 0.53, *p* = 0.003, *n* = 29), but not in patients with ME/CFS (*r* = 0.31, *p* = 0.144, *n* = 23) or long COVID (*r* = 0.27, *p* = 0.231, *n* = 21). Bars represent 100 µm. Oxidative phosphorylation data in the patient cohort remained non-normally distributed following Box-Cox transformation. Two-sided paired t-tests were used to assess parametric data from the bed rest cohort. Non-parametric data from the bed rest cohort was assessed using two-sided Mann–Whitney *U* tests. Analysis of variance, with Tukey HSD post-hoc testing or Kruskal–Wallis *H* test, with pairwise Wilcoxon tests with Benjamini–Hochberg correction post-hoc, was used to assess parametric and non-parametric data for patient cohorts. Linear relationships were assessed using Pearson’s correlation on non-transformed data; solid lines represent significant correlations (*p* < 0.05). Box plots display individual data points, the median (center line), and the first and third quartiles (lower and upper bounds of the box). Pre-bed rest and healthy controls are displayed in white, post-bed rest is displayed in gray, patients with long COVID are displayed in orange, and patients with ME/CFS are displayed in green.

Whilst we found evidence of either oxygen extraction or stroke volume being impeded in patients, we did not have the resources to measure stroke volume. Thus, we focused on parameters related to oxygen extraction and utilization in the skeletal muscle. Given that physical activity has been shown to shift limitations of maximal aerobic capacity from oxygen utilization to oxygen supply^26^ and the association between mitochondrial function and whole-body aerobic exercise capacity^27^, we assessed mitochondrial respiration of permeabilized muscle fibers and SDH activity in sections to provide further insight into mitochondrial alterations upon bed rest and in patients. Bed rest resulted in lower SDH activity and oxidative phosphorylation capacity (Fig. 3A, B). Compared to healthy controls, only patients with ME/CFS, but not long COVID, exhibited lower SDH activity (Fig. 3A). Both patients with long COVID and ME/CFS presented with lower oxidative phosphorylation capacities compared to healthy controls (Fig. 3B). As mitochondria are the ultimate recipients in the oxygen cascade, we hypothesized that either SDH activity or oxidative phosphorylation correlated with $\dot{V}{O}_{2\max }$. Both SDH activity and oxidative phosphorylation capacity were correlated with $\dot{V}{O}_{2\max }$ in healthy controls (Fig. 3C, D), and such a relation was present following bed rest. However, this association was absent in patients with long COVID and ME/CFS. While the precise limitation of $\dot{V}{O}_{2\max }$ in patients is obscured, the lack of association of $\dot{V}{O}_{2\max }$ with mitochondrial variables suggests that reductions in exercise capacity are not limited by mitochondrial density or respiratory capacity per se.

As mitochondrial respiration reflects both mitochondrial content and function, we subsequently normalized mitochondrial respiration to gain insight into alterations of intrinsic mitochondrial function. After normalizing mitochondrial respiration to SDH activity, there was no effect of bed rest on oxidative phosphorylation capacity (Supplementary Fig. 8A). In contrast, patients with long COVID still displayed lower oxidative phosphorylation capacity normalized to SDH activity compared to healthy controls, while patients with ME/CFS tended to exhibit lower oxidative phosphorylation capacity (*p* = 0.063, Supplementary Fig. 8A). Normalizing leak respiration to maximal uncoupled respiration (E-L coupling efficiency) is reflective of intrinsic mitochondrial biochemical coupling, wherein a less coupled system (lower values) indicates higher proton leak. Bed rest did not alter the E/L coupling efficiency; however, both patients tended to display lower E/L coupling efficiency compared to healthy controls (*p* = 0.066, Supplementary Fig. 8B). The relative NADH-linked respiration (NADH-linked respiration normalized to oxidative phosphorylation capacity) increased, and the relative succinate-linked respiration (succinate-linked respiration normalized to maximal uncoupled respiration) decreased after long-term bed rest (Supplementary Fig. 8C-D). No such differences were observed between patients and healthy controls.

Bed rest reduced maximal oxidative phosphorylation, consistent with lower SDH activity, and slightly altered intrinsic respiration, suggesting a shift in the contributions of complex I and II. In contrast, the lower oxidative phosphorylation capacity in both patient groups was accompanied by a lower coupling efficiency with no changes to qualitative changes in the electron flux through complex I or complex II.

### Skeletal muscle capillarization

**Fig. 4:**
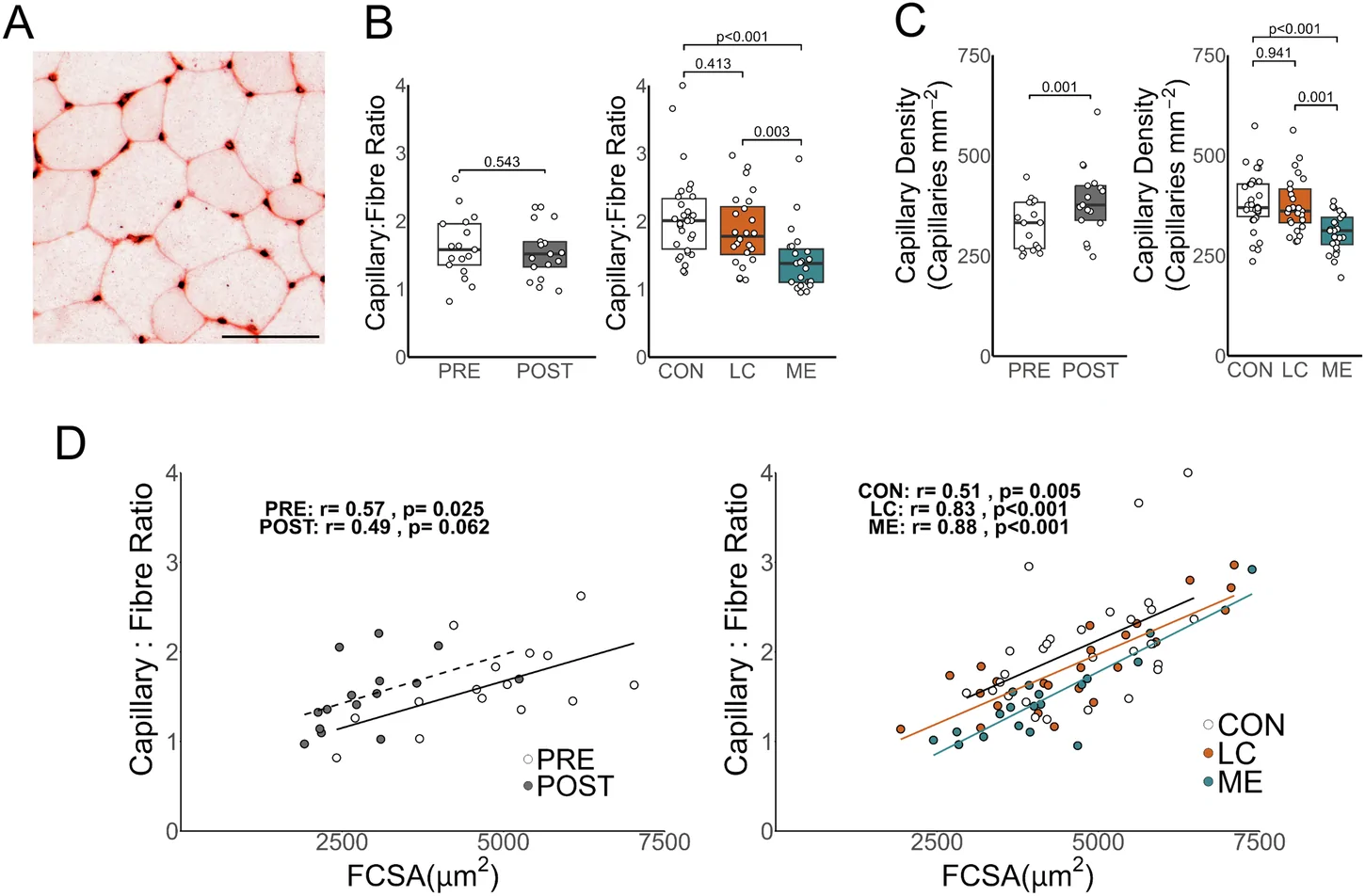
Capillary-to-fiber ratio following bed rest is proportional to muscle fiber size, while both patients with long COVID and ME/CFS have reduced capillary-to-fiber ratio for a given fiber size. A typical example of a lectin stain for capillaries is shown in (**A**). The scale bar represents 100 µm. Following bed rest, capillary-to-fiber ratio was not significantly different (**B**; *p* = 0.543, *n* = 17), while patients with myalgic encephalomyelitis/chronic fatigue syndrome (ME/CFS; ME, *n* = 23) had a significantly lower capillary-to-fiber ratio compared to patients with long COVID (*p* = 0.001, *n* = 24) and healthy controls (*p* < 0.001, *n* = 30; B). Capillary density (**C**) was significantly increased following bed rest (*p* = 0.001, *n* = 17), while both patients with long COVID (*n* = 25) and patients with ME/CFS (*n* = 24) had significantly lower capillary densities compared to healthy controls (**D**; *p* < 0.001, *n* = 30). The relationship between fiber cross-sectional area (FCSA) and capillary-to-fiber ratio significantly correlated in all conditions, except post-bed rest (albeit *p* = 0.062 following bed rest). The relationship between FCSA and capillary-to-fiber ratio was not significantly different following bed rest (*z* = 0.495, *p* = 0.621); however, compared to healthy controls, both patients with long COVID (*z* = −2.176, *p* = 0.030) and patients with ME/CFS (*z* = −2.602, *p* = 0.009) displayed significantly different relationships (G). Capillary density and capillary-to-fiber ratio data were normally distributed following Box-Cox transformation. Two-sided paired t-tests were used to assess parametric data from the bed rest cohort. Non-parametric data from the bed rest cohort was assessed using two-sided Mann–Whitney *U* tests. Analysis of variance, with Tukey HSD post-hoc testing or Kruskal–Wallis *H* test, with pairwise Wilcoxon tests with Benjamini–Hochberg correction post-hoc, was used to assess parametric and non-parametric data for patient cohorts. Linear relationships were assessed using Pearson’s correlation on non-transformed data. Solid lines represent significant correlations, dashed lines represent correlations with *p* < 0.10. Box plots display individual data points, the median (center line), and the first and third quartiles (lower and upper bounds of the box). Pre-bed rest and healthy controls are displayed in white, post-bed rest is displayed in gray, patients with long COVID are displayed in orange, and patients with ME/CFS are displayed in green.

As bed rest induced muscle fiber atrophy and patients exhibited a lower proportion of type I fibers, we hypothesized that capillary supply may be differentially affected. Following bed rest, the average capillary-to-fiber ratio was unchanged (Fig. 4B); however, the cross-sectional area of each capillary was significantly reduced (Supplementary Fig. 9A). Due to greater muscle atrophy relative to capillary loss^19^, capillary density was increased following long-term bed rest (Fig. 4C). These changes were not observed in patients with long COVID or ME/CFS. Patients with ME/CFS displayed lower capillary-to-fiber ratios and capillary densities (Fig. 4C) compared to healthy controls and patients with long COVID, while capillary measures in patients with long COVID were not different from those of healthy controls. The relationship between muscle fiber size and the capillary-to-fiber ratio is reflective of adaptive remodeling to support the tight link between these two parameters to maintain adequate diffusive oxygen supply in larger fibers^28^. As we found differential intrinsic mitochondrial alterations between patients and bed rest, we queried whether alterations of mitochondrial capacities and capillarization subsequently were reflected by an altered capillary-fiber size relationship. In all groups, capillary-to-fiber ratios were proportional to FCSA, with a trend following long-term bed rest (*p* = 0.062; Fig. 4D). Both patient groups had significantly lower intercepts than healthy controls (both *p* < 0.05), indicating less capillary supply for a given fiber cross-sectional area.

Due to the selective type I fiber atrophy in patients, we queried whether the capillary alterations were different by fiber type. To this end, we calculated the capillary-to-fiber ratio on a fiber-type basis, comparing type I to type II fibers (Supplementary Fig. 11) in a subgroup of participants. In all conditions, type I fibers had a greater capillary-to-fiber ratio than type II fibers. Bed rest reduced capillary-to-fiber ratio in both type I and type II fibers equally. ME/CFS patients had lower capillarization compared to both healthy controls and long COVID patients, independent of fiber type. There was no effect of disease on fiber-type-specific capillarization.

Due to the reduced capillary-to-fiber ratio for a given fiber size, despite no fiber-specific alterations to capillary indices, we queried whether intramyocyte diffusive parameters may be altered. To this end, we evaluated the intracellular myoglobin content in a subset of participants. Following bed rest, myoglobin content increased unexpectedly (Supplementary Fig. 10B), whereas patients and healthy controls did not differ (Supplementary Fig. 10C).

Therefore, the results indicate that the capillary number for a given FCSA remains largely unchanged after long-term bed rest. In both patient groups, however, capillary number for a given FCSA was lower, with patients with ME/CFS exhibiting an overall reduction in capillary-to-fiber ratios, independent of fiber type. Myoglobin content was not different between patients and healthy controls.

### Comparing skeletal muscle alterations in long COVID and ME/CFS with long-term bed rest

Since both patient groups had lower daily physical activity levels than healthy controls, we reasoned that the healthy participants after bed rest could serve as an alternative control group. We therefore analyzed a subgroup of 13 patients with ME/CFS, 13 patients with long COVID, and 13 age- and sex-matched healthy individuals following long-term bed rest (participant characteristics outlined in Supplementary Table 1).

$\dot{V}{O}_{2\max }$ did not differ between groups (Supplementary Fig. 12A), however patients with long COVID tended to have a higher $\dot{V}{O}_{2\max }$ than post-bed rest individuals when expressed relative to age- and sex-predicted values (ANOVA: *p* = 0.076, post-hoc: *p* = 0.031 Supplementary Fig. 12B). Patients with ME/CFS had significantly lower GET than those with long COVID and healthy post-bed rest individuals (Supplementary Fig. 12C). GET occurred at a lower relative intensity in patients with ME/CFS than in both patients with long COVID and participants following bed rest (Supplementary Fig. 12D).

Participants after bed rest had significantly smaller FCSA compared to both patient groups (Supplementary Fig. 12E). Patients with long COVID displayed higher capillary-to-fiber ratios, SDH activity, and oxidative phosphorylation capacity compared to participants after bed rest and patients with ME/CFS (Supplementary Fig. 12F–H).

When assessing the relationships between capillary-to-fiber ratio and FCSA, only patients with long COVID displayed a significant relationship, while patients with ME/CFS showed a similar trend (Supplementary Fig. 12I). Conversely, only participants after bed rest displayed a significant correlation between $\dot{V}{O}_{2\max }$ and SDH activity and oxidative phosphorylation capacity (Supplementary Fig. 12J, K). These results suggest that capillary supply and mitochondrial function are disrupted differently in long COVID and ME/CFS compared to bed rest-induced deconditioning.

Thus, when retrospectively comparing patients to age- and sex-matched participants after bed rest, patients with long COVID and ME/CFS still display alterations that are different than those found after bed rest. Despite similar $\dot{V}{O}_{2\max }$, patients show no associations with skeletal muscle oxidative function compared to those observed with bed rest.

## Discussion

The major finding of the present study is that acute exercise responses and skeletal muscle alterations in long COVID and ME/CFS differ from those caused by bed rest-induced deconditioning per se. Despite similar whole-body exercise capacity in both patient groups and participants after bed rest, the pulmonary and cardiovascular responses to acute exercise were distinct. Long-term (60 days) bed rest induced severe muscle atrophy, which was not observed in patients with long COVID or ME/CFS. Instead, both patient groups displayed a lower proportion of type I fibers, whereas only patients with ME/CFS exhibited lower capillary-to-fiber ratios and type I-specific atrophy. Further, the lower oxidative phosphorylation capacity in both patient groups did not correlate with $\dot{V}{O}_{2\max }$, in contrast to bed rest participants, suggesting that physiological impairments distinct from inactivity drive reduced exercise capacity in patients. These findings challenge the notion that deconditioning is the primary driver of reduced exercise capacity in long COVID and ME/CFS. Rather, our observations suggest intrinsic skeletal muscle abnormalities contribute to limited exercise capacity, including reduced capillary supply relative to muscle fiber size and mitochondrial impairments.

Maximal aerobic capacity was similar between patients with long COVID and ME/CFS and those undergoing a strict 60-day bed rest. However, the physiological determinants underpinning this reduction differed. Ventilatory responses to acute exercise differed following bed rest compared to the patient groups. Bed rest resulted in reduced $\dot{V}{E}_{\max }$, consistent with the decrease in $\dot{V}{O}_{2\max }$, whereas the $\dot{V}{E}_{\max }/\dot{V}C{O}_{2\max }$ and the $\dot{V}E-\dot{V}C{O}_{2}$ slope were increased. Patients only exhibited higher $\dot{V}E-\dot{V}C{O}_{2}$ slope during submaximal exercise, and a tendency of lower $\dot{V}{E}_{\max }$ and higher $\dot{V}E-\dot{V}C{O}_{2}$ slope for the entirety of the exercise test. While arterial PCO_2_ was not measured, reductions in $\dot{V}{E}_{\max }$ and increases in $\dot{V}{E}_{\max }/\dot{V}C{O}_{2\max }$ and the $\dot{V}E-\dot{V}C{O}_{2}$ slope at submaximal exercise suggests earlier indications of hyperpnea^29–31^ in patients. The causes of these findings are currently unclear; however, reduced blood or muscle CO_2_ buffering capacity^32^, increased chemoreceptor sensitivity^33^, or pulmonary vascular dysfunction resulting in increased dead space ventilation^34^ may contribute.

Acute cardiovascular responses to exercise also differed between cohorts. Previous studies report that deconditioning typically leads to an increase in the HR-$\dot{V}{O}_{2}$ slope^20^, reflecting a greater heart rate increase per unit change in $\dot{V}{O}_{2}$, which is often associated with reduced oxygen extraction^20,35,36^. However, in our study, bed rest participants did not present with this pattern, which may be attributed to the disproportionate increase in baseline heart rate (+30%) relative to their maximal heart rate (+5%) and reduction in $\dot{V}{O}_{2\max }$ (−24%) following bed rest. The increase in both resting and maximal heart rate following bed rest, which has been noted previously^37–39^, is not fully understood, but may be related to reductions in plasma and stroke volume^38^, altered baroreflex function^39^, or shifts in the balance between parasympathetic and sympathetic nervous system activity^37^observed after bed rest. In contrast, our patient groups exhibited an increased HR-$\dot{V}{O}_{2}$ slope, suggesting a greater reliance on changes in heart rate to satisfy a given oxygen demand. While this may be indicative of either decreased stroke volume or reduced peripheral oxygen extraction^20^, it is consistent with results from invasive CPET studies, which have shown preload failure and marked reduction in peripheral oxygen extraction in long COVID and ME/CFS patients^40,41^. Future studies in long COVID and ME/CFS should focus on measuring markers of cardiovascular function during exercise, such as stroke volume, peripheral oxygen extraction, and baroreflex sensitivity, to further elucidate the underlying mechanism.

We showed that the reduction in oxidative phosphorylation capacity following bed rest was primarily attributed to a reduction in SDH activity, often used as a proxy for mitochondrial density^42^. In contrast, the reduced oxidative phosphorylation capacity in both patient groups was still present following normalization to SDH activity, suggesting that intrinsic mitochondrial respiration may be primarily affected. Furthermore, while E/L coupling efficiency tended to be reduced in both patient groups, this was not observed following bed rest. These results suggest that skeletal muscle oxidative impairments in long COVID and ME/CFS cannot solely be attributed to deconditioning. Indeed, previous work suggests abnormal ultrastructure, particularly reduced cristae density, in patients with long COVID and ME/CFS^11^, while bed rest is typically associated with more fragmented mitochondria^16^. What causes the intrinsic mitochondrial alterations in long COVID and ME/CFS is currently unclear. Future work is required to quantify skeletal muscle mitochondrial ultrastructure in long COVID and ME/CFS.

Notably, oxidative phosphorylation capacity and SDH activity correlated with whole-body maximal oxygen uptake in healthy individuals (before and after bed rest), whereas these were absent in long COVID and ME/CFS. This suggests that factors beyond skeletal muscle mitochondrial respiration constrain exercise capacity in long COVID and ME/CFS, including impairments in endothelial function^43–47^, a reduced venous return and cardiac preload^40^, or a reduction in muscle diffusive capacity^48^ contributing to impaired peripheral oxygen extraction^41^. Conversely, mitochondrial respiration contributes to $\dot{V}{O}_{2\max }$ in healthy sedentary, untrained individuals^26,49^, which is consistent with the associations observed between mitochondrial markers and $\dot{V}{O}_{2\max }$ across all healthy cohorts. Thus, it is likely the constraints on $\dot{V}{O}_{2\max }$ imposed by bed rest, long COVID and ME/CFS differ, implying that the reduced aerobic exercise capacity in long COVID and ME/CFS cannot solely be ascribed to deconditioning.

Capillary-to-fiber ratios and capillary density were lower in patients with ME/CFS, but not long COVID, compared to healthy controls, reducing the structural potential to adequately supply oxygen and nutrients and remove metabolic byproducts during exercise. These findings are contrary to previous reports of lower capillary densities in long COVID^9,50^. While capillary-to-fiber ratio was unaltered following bed rest, capillary density increased due to significant muscle atrophy^19,51^, consistent with the principle that alterations in muscle size typically occur before capillary alterations^19,52,53^. Intriguingly, the average area of individual capillaries was reduced following bed rest (Supplementary Fig. 9A). This may be due to reduced diameter or capillary tortuosity^54,55^, resulting in less obliquely cut capillaries, or due to the lack of shear stress experienced during head-down tilt, resulting in a structural remodeling of the capillary beds^56^. That skeletal muscle microvascular-endothelial function was impaired following a 10-day bed rest^57^ is consistent with this notion. Although we report unaltered structural indices of capillarization in long COVID, it is unknown whether skeletal muscle capillary perfusion (and oxygen diffusion) was altered during exercise in either long COVID or ME/CFS, as direct measurements of skeletal muscle capillary blood flow are challenging to assess in humans in vivo.

Capillary-to-fiber ratio is related to muscle FCSA, wherein larger muscle fibers typically have more capillary contacts to accommodate oxygen and nutrient supply, and metabolite removal^52,53^. While these relations existed in all groups, except following bed rest, this relationship was downshifted in both long COVID and ME/CFS patients compared to healthy controls. This suggests that fewer capillaries are available per unit fiber area in patients, which is distinct from the capillary alterations following strict bed rest^19^. This reduces the surface area and spatial distribution for gas exchange at high metabolic rates and represents a structural limitation to oxygen diffusion, irrespective of upstream regulation of blood flow at the arteriolar level. Future work on endothelial alterations in long COVID and ME/CFS might provide further mechanistic insight^58^.

$\dot{V}{O}_{2\max }$ is determined by the integrated function of convective oxygen delivery, diffusive oxygen transport, and mitochondrial oxygen utilization, as conceptualized in the Wagner diagram^59,60^. Although we measured several components of this pathway, we did not assess all sites required to localize the dominant limitation. Importantly, while mitochondrial oxidative capacity was reduced in patients, its lack of association with $\dot{V}{O}_{2\max }$ (in contrast to healthy controls and bed rest participants) argues against a purely mitochondrial limitation. Instead, our findings are consistent with the presence of interacting limitations upstream of the mitochondria, including impaired muscle oxygen diffusive capacity or maximal convective oxygen delivery, which cannot be resolved within the current dataset. Notably, reduced whole-body oxygen extraction does not necessarily imply low intramyocyte PO_2_, as a reduction in mitochondrial oxygen utilization would be expected to reduce the diffusion gradient required to support $\dot{V}{O}_{2}$ rather than increase it. Localized reductions in tissue PO_2_ would therefore require sufficiently impaired diffusive conductance or microvascular perfusion heterogeneity, mechanisms that were not directly assessed here. This situation contrasts with healthy sedentary individuals, where mitochondrial capacity is the primary limitation to $\dot{V}{O}_{2\max }$ in small muscle mass exercise (i.e., under conditions where cardiovascular limitations are minimized)^26^. Although we utilized whole-body exercise in the current study, and the mitochondrial oxidative ceiling is far less likely to be reached in this mode of exercise, our observation of the significant association between SDH activity and oxidative phosphorylation and $\dot{V}{O}_{2\max }$ that occurs after bed rest (Fig. 3C), and across different populations^27^ is at least consistent with the notion of a role for mitochondria in explaining the variation of $\dot{V}{O}_{2\max }$ in patient populations.

Invasive CPET results indicate that at maximal exercise, arterial PO_2_ in long COVID and ME/CFS is similar to that in healthy people^40,41^. Intramyocyte oxygen movement is dependent on convective oxygen delivery, oxygen diffusion through the tissue, and mitochondrial oxygen consumption^61^. The decrease in capillary-to-fiber ratio for a given fiber size reduces the potential for oxygen convective delivery. While the reduced mitochondrial oxidative capacity in both patients with long COVID and ME/CFS would suggest a higher intracellular PO_2_, we were not able to measure intracellular PO_2_ directly. However, as myoglobin is an integral piece in intramyocyte oxygen diffusion, we suspected that if intracellular PO_2_ was indeed higher and oxygen convective delivery was impaired, there may be a compensatory response of myoglobin in order to maintain the oxygen diffusion gradient. As myoglobin content was similar amongst the groups, it would suggest that intramyocyte diffusion capacity may be maintained. However, it is unknown whether any of the hemoglobin, capillary, or myocyte membrane function is altered, which also impacts oxygen diffusive capacity. Future work should aim to address whether membrane functions are altered and may contribute to impaired oxygen extraction. We confirmed that bed rest causes significant atrophy across all fiber types^62–64^. Conversely, overall FCSA in patients with long COVID and ME/CFS was not different from that of healthy controls. However, we observed selective type I fiber atrophy in patients with ME/CFS, suggesting fiber-type-specific vulnerability to atrophy. The underlying mechanism remains unclear, as we lack the longitudinal data to determine whether these changes result from disease progression or pre-disease differences.

Previous studies show conflicting results regarding changes in fiber-type composition following bed rest^19,65–68^. In our cohort, fiber-type composition remained unchanged following bed rest, calculated as both the area occupied by fiber type and the numerical proportion of fibers of a given type relative to all fibers present. In contrast, both patients with long COVID and ME/CFS exhibited higher proportions of type IIa/IIx fibers and lower proportions of type I fibers compared to healthy controls, expressed both as relative to area occupied and total number of fibers. Since we did not obtain longitudinal muscle biopsies from patients, we cannot determine whether this is due to a fiber-type transition or whether individuals with higher proportions of type IIa/IIx fibers are predisposed to developing long COVID or ME/CFS. However, given the type I fiber-specific atrophy pattern observed in patients with ME/CFS, the lower proportion of type I fibers and higher proportions of type IIa/IIx fibers in patients, our findings suggest that a shift toward type IIa/IIx fibers may occur in ME/CFS. The type I atrophy and the higher prevalence of type IIa/IIx fibers were more pronounced in ME/CFS compared to long COVID patients, possibly because ME/CFS patients were diagnosed before the COVID pandemic and therefore were ill for a significantly longer time. Whether the disease duration or intrinsic differences between long COVID and ME/CFS underlie these differences is therefore unknown.

Previous studies have indicated an overlap in potential pathological mechanisms in both long COVID and ME/CFS, which include (but certainly not limited to) autonomic dysfunction, microclot formation in the blood, endothelial dysfunction, and mitochondrial dysfunction^69^. In our current study, we provide evidence for skeletal muscle mitochondrial impairments to be implicated in both long COVID and ME/CFS. However, as both diseases are heterogeneous, other mechanisms may be involved depending on clinical presentation. In our current cohort, we focused on long COVID patients who exhibited PEM as a key symptom. Thus, it may be that the mitochondrial dysfunction we highlight is more unique to those patients who exhibit PEM rather than other long COVID symptoms.

The current study indicates that skeletal muscle adaptations and lower aerobic exercise capacities observed in patients with long COVID and ME/CFS are different from those occurring after bed rest; however, there are several limitations. A limitation of the present study is its cross-sectional design in patients with long COVID and ME/CFS, which precludes inference on temporal disease progression or causality. While longitudinal muscle biopsies would be informative, such data are currently unavailable, and ethical constraints limit experimental manipulation of activity levels in patients. Consequently, our conclusions are restricted to demonstrating that the skeletal muscle phenotype observed in patients differs from that induced by prolonged bed rest, rather than establishing the temporal origin of these abnormalities. We did not induce physical inactivity in patients with ME/CFS or long COVID. Patients are typically more sedentary because PEM worsens both their symptoms and skeletal muscle abnormalities^6^. We also cannot completely exclude the contribution of physical inactivity to the skeletal muscle alterations observed in patients with long COVID and ME/CFS; however, our results do indicate that physical inactivity alone is insufficient to explain the skeletal muscle changes in patients. There was likely self-selection bias toward patients with milder symptoms, as they volunteered to travel to the laboratory for multiple occasions and undergo a maximal exercise test. Our results are therefore not directly applicable to home-bound patients^70^. Similarly, we were unable to account for training status before disease onset. We do not know if previous training status influences susceptibility to skeletal muscle alterations^71,72^. Additionally, the current research design precludes us from drawing conclusions about the differences in pathophysiology of long COVID and ME/CFS. ME/CFS patients were diagnosed before 2020 and, therefore, were ill for a significantly longer period. Therefore, we cannot distinguish between disease duration and disease pathophysiology. While we only found a lower capillarization in patients with ME/CFS, we did not assess qualitative markers for microvascular blood flow or red blood cell flux through capillaries in long COVID or ME/CFS. While we attempted to account for any sex differences, we cannot exclude any sex-specific alterations that may occur during bed rest due to an underpowered comparison. A further limitation is the use of head-down tilt bed rest as a comparator for clinical deconditioning. Although this model enables long-term experimental control of physical inactivity, it introduces hemodynamic alterations that differ from those experienced by ambulatory patients, particularly with respect to fluid redistribution and orthostatic loading. As such, conclusions drawn from the bed rest comparison are most robust for skeletal muscle structural and metabolic outcomes, whereas cardiovascular interpretations are necessarily more constrained. Additionally, head-down tilt bed rest may differently affect women due to sex-specific hormones on blood pressure regulation^73^.

This study demonstrates that the skeletal muscle determinants of exercise capacity in long COVID and ME/CFS differ from those induced by prolonged bed rest. While $\dot{V}{O}_{2\max }$ and markers for aerobic exercise capacity were similarly low in patients with long COVID and ME/CFS compared to long-term bed rest, respiratory and cardiovascular responses to acute exercise were distinct. Patients with long COVID and ME/CFS displayed higher proportions of type IIa/IIx fibers and signs of intrinsic mitochondrial dysfunction, observations that were not seen following bed rest. These findings indicate that the lower exercise capacity in patients with long COVID and ME/CFS is not solely due to physical inactivity; therefore, rehabilitation strategies for patients should consider that patients with long COVID and ME/CFS are not simply deconditioned and should be treated as unique cases with alternative rehabilitation strategies. As mitochondrial and endothelial dysfunction appear prominent in both long COVID and ME/CFS, with clear implications for oxygen extraction, future studies should aim to investigate the viability of improving patient mitochondrial and/or endothelial health to reduce patient burden.

## Methods

### Study approval and previous clinical trial primary outcomes

Two cohorts were compared: one consisting of 24 bed rest participants undergoing a strict 60-day head-down tilt bed rest (occurring before 2020), and one consisting of a cross-sectional design cohort of 25 patients with long COVID, 26 patients with ME/CFS, and 30 age- and sex-matched healthy controls that had successfully recovered from an acute SARS-CoV-2 infection. The comparison between the two different cohorts was done as a non-pre-specified exploratory analysis, approved by the Amsterdam UMC Medical Research Ethics Committee as an amendment. Sex was considered in both study designs. Recruitment for the bed rest study aimed to obtain a 50:50 sex divide, but since only 8 women were included, sex was used as an exploratory variable. The patient cohort study aimed to age- and sex-match all patient participants with healthy controls, thus reducing the influence of sex. As such, no sex-specific analysis was conducted in either cohort. Sex was determined by self-report by participants.

Participants undergoing bed rest were part of the AGBRESA study (registered at DRKS00015677)^19^. The protocol was approved by the ethics commissions of the Medical Association North Rhine (number 2018143) and NASA (Johnson Space Center, Houston, United States). The AGBRESA study was registered as a clinical trial with the aim of investigating the effects of continuous and intermittent centrifugation on 6° head-down tilt bed rest. Written informed consent was obtained from all participants and included the reuse of data for subsequent studies. The primary outcomes from this study have been published previously^16,19,74–76^.

Patients with long COVID and patients with ME/CFS were part of a case-control study in the Amsterdam University Medical Center (AUMC) and the Faculty of Behavioral and Movement Sciences (Vrije Universiteit Amsterdam). The study protocol was approved by the medical ethics committee of the Amsterdam UMC (NL78394.018.21) and registered at www.clinicaltrials.gov (NCT05225688). All participants signed a written informed consent before participation. The study was conducted in accordance with the Declaration of Helsinki. The primary outcomes of this trial were (1) skeletal muscle mitochondrial respiratory function before and 24-h following maximal aerobic exercise and (2) to investigate local and systemic inflammation following maximal aerobic exercise to understand post-exertional malaise in patients with long COVID and ME/CFS (in an amendment). Data pertaining to 21 healthy controls and 25 long COVID patients have been previously published^6^. Primary outcome measurements related to post-exertional malaise in patients with ME/CFS have not yet been published. Here, we present the baseline mitochondrial parameters from patients with ME/CFS.

### Study populations

#### Bed rest

The study details have been reported previously^19^. Briefly, 24 healthy participants underwent a 60-day strict 6 degrees head-down tilt bed rest (Table 1). Participants followed standardized diets, consuming 1.6 times their resting metabolic rate before bed rest and 1.3 times their resting metabolic rate during bed rest. Fluid intake was controlled, and ingestion of caffeine and alcohol was prohibited.

### Long COVID and ME/CFS cohort

Patients with long COVID were diagnosed by two experienced clinicians for long COVID symptomology and exclusion of potential differential diagnoses. All long COVID patients were diagnosed with post-exertional malaise (PEM) by the DSQ-PEM^77^, had a minimum period of long COVID-related symptoms of six months, and were between 18 and 65 years old. No symptoms were present before the confirmed diagnosis of SARS-CoV-2. None of the included participants were admitted to the hospital during acute SARS-CoV-2 infection and were healthy prior to NAAT or serology-proven SARS-CoV-2 infection. Reported symptom durations (Table 1) were from symptom onset to study enrollment (February 2022 for long COVID and October 2023 for ME/CFS). Exclusion criteria were a medical history of cardiovascular/pulmonary disease, diabetes mellitus, concurrent treatment with metabolism- or coagulant-altering drugs during the study period (statins, corticosteroids, SGLT2 inhibitors, GLP1 receptor agonists, platelet aggregation blockers and any anticoagulants), adiposity and body mass index >35 (related to muscle biopsy obtainment), pregnancy, active infection, severe renal dysfunction or any other prior chronic illness or >6 alcohol units per day or >14 alcohol units per week. Smoking was not an exclusion criterion. One long COVID patient was excluded due to a recent SARS-CoV-2 re-infection (<7 days).

Patients with ME/CFS all fulfilled the Canadian Consensus Criteria (CCC), exhibited PEM according to the DSQ-PEM, consulted with an ME/CFS specialist or post-COVID physician, and were between 18 and 65 years old. ME/CFS diagnosis was prior to 2020, and all patients had a confirmed previous diagnosis of SARS-CoV-2. Exclusion criteria were identical to those of the patients with long COVID.

None of the healthy controls had residual symptoms after the SARS-CoV-2 infection, and none were hospitalized within 6 months of study participation. Healthy controls withdrew because of the invasive nature of the protocol (*n*  =  2), symptoms related to burnout (*n*  =  1), and a novel diagnosis of uncontrolled hypertension (*n*  =  1).

### Cardiopulmonary exercise testing

All participants underwent cardiopulmonary exercise testing (CPET) on an electronically braked cycle ergometer to assess aerobic exercise capacity. Generally, each test was preceded by a rest on the ergometer, followed by a baseline cycling phase before beginning the incremental exercise test.

Bed rest participants performed a step incremental test (Lode Excalibur, Lode, Groningen, The Netherlands), whereas patients with long COVID and ME/CFS performed a ramp incremental exercise test (Lode Excalibur Sport, Lode, Groningen, The Netherlands; Monark L7TT, Monark Sports & Medical, Vansbro, Sweden). Step and ramp protocols have been reported to yield comparable $\dot{V}{O}_{2\max }$ values^78^.

#### Step Test Protocol (bed rest cohort)

Participants first completed 5-min of quiet rest on the cycle ergometer, followed by 3-min of baseline cycling at 50 W. Power output then increased by 25 W min^-1^ until task failure. Participants were instructed to maintain 75 RPM, and task failure was defined as the point at which cadence dropped below 70 RPM despite verbal encouragement.

#### Ramp incremental test (long COVID & ME/CFS cohort)

Participants completed a 2-min quiet rest on the ergometer and 4-min baseline cycling at low intensity between 0 and 20 W. This was followed by a ramped, linear increase in power output (10–60 W min^-1^) until task failure. The individual baseline work rates and ramp slopes were selected based on each participant’s anthropometric characteristics and physical activity levels and designed to elicit task failure within 8–12 min. Participants were instructed to maintain their cadence between 70 and 90 revolutions/min, and task failure was defined as the point at which cadence dropped below 60 revolutions/min despite verbal encouragement. Capillary lactate concentrations were determined at rest prior to the onset of the test, during baseline cycling, and immediately following task failure (Lactate Pro 2 LT-1730, ARKRAY Ltd., United Kingdom). Pulmonary gas exchange and ventilation were measured on a breath-by-breath basis (Bed rest: Innocor, Innovision, Odense, Denmark; Patients with long COVID and patients with ME/CFS: Cosmed Quark CPET; Cosmed, Rome, Italy). In all studies, $\dot{V}{O}_{2\max }$ was defined as the highest 30 s rolling average $\dot{V}{O}_{2}$ achieved during the test. Achievement of $\dot{V}{O}_{2\max }$ was primarily determined by the presence of a plateau in the $\dot{V}{O}_{2}$ as defined by either: (1) increase of $\dot{V}{O}_{2}$ < 100 ml/min in the final 2-min of testing^79^, (2) a $\dot{V}{O}_{2}$ slope in the final 2-min of exercise that was >50% different of the extrapolated $\dot{V}{O}_{2}$ slope prior to the gas exchange threshold^80^, or (3) data points in the final 2-minlying outside of the 95% confidence interval of the extrapolated $\dot{V}{O}_{2}$ slope prior to the gas exchange threshold^79^. Secondary endpoint criteria were also assessed, namely achievement of (1) maximal age-predicted heart rate, (2) blood lactate >8.0 mM, and (3) respiratory exchange ratio >1.15.

The gas exchange threshold (GET) was assessed using 10-s binned averages as the point of non-linear rise in $\dot{V}{{CO}}_{2}$ relative to $\dot{V}{O}_{2}$ using the V-slope method^81^, and verified with visual inspection. The GET was then confirmed visually by assessing $\dot{V}E$ vs $\dot{V}{O}_{2}$, $\dot{V}E/\dot{V}{{CO}}_{2}$ vs $\dot{V}{O}_{2}$, and $\dot{V}E/\dot{V}{O}_{2}$ vs $\dot{V}{O}_{2}$. Respiratory compensation point (RCP) was assessed as the point of non-linear rise in $\dot{V}E$ relative to $\dot{V}{O}_{2}$ using the V-slope method, and again verified visually. Presence of the RCP was confirmed by visual inspection of the $\dot{V}E/\dot{V}{{CO}}_{2}$ vs $\dot{V}{O}_{2}$ and PetCO_2_ vs $\dot{V}{O}_{2}$.

Maximal acute exercise values were taken as the highest 30-s rolling averages of the respective parameters. The $\dot{V}E/\dot{V}{{CO}}_{2}$ slope was calculated as the linear rise until RCP. Heart rate-$\dot{V}{O}_{2}$ slopes were calculated using the entirety of the exercise test and taken as the slope of heart rate compared to $\dot{V}{O}_{2}$. Baseline heart rate was calculated using an average over the 5-min quiet rest, with the first 30-s and last 30-s being disregarded. Adjusted heart rate reserve was calculated as the (HR_max_- HR_baseline_)/(220- age-HR_baseline_).

Due to software issues, three patients with ME/CFS undertook ramp rates that were too high, resulting in a duration of exercise that was not sufficient to establish acute exercise responses. Evaluation of maximal exercise was still possible in these participants; thus, submaximal values were excluded.

### Skeletal muscle measurements

#### Biopsy procedure

Muscle biopsies were obtained from the vastus lateralis at one-third of the distal length (approximately 150 mg weight wet) using a rongeur (4 mm diameter for the bed rest cohort) or a suction-supported 5 mm Bergström needle (Pelomi, Albertslund, Denmark; for the long COVID & ME/CFS patient cohort) under sterile conditions after skin disinfection and local anesthesia with 2% lidocaine^6,16^. Muscle biopsies were taken before and after 55 days of head-down tilt bed rest. One ~30 mg piece was aligned according to the muscle fiber arrangement under a light microscope and frozen in liquid nitrogen for histochemistry, and another ~15 mg piece was utilized for high-resolution respirometry experiments. Muscle biopsies from patients and healthy controls were obtained 6 days prior to the CPET, and all participants were instructed to refrain from strenuous activity at least 24 h prior to the biopsy procedure.

All biopsies used for histochemistry were frozen in liquid nitrogen and stored in liquid nitrogen at −196 °C. Frozen muscle samples were mounted using freeze-gel (Q Path, VWR, Netherlands) and cut into 10-µm-thick sections in transverse orientation in a cryostat (Microm HM550, Adamas Instrumenten, Netherlands) at −20 °C, before being mounted on polylysine-coated slides and stored at −80 °C until staining. Due to issues with tissue freezing, histological analysis was completed on samples from 17 participants pre- and post-bed rest, 24 patients with long COVID, 23 patients with ME/CFS, and 30 healthy controls.

### Skeletal muscle respirometry

Mitochondrial respiration was assessed in permeabilized fibers as described previously^6^. Small bundles of freshly isolated fibers were permeabilized with 50 µg mL^−1^ saponin for 20 min at 4 °C in a solution consisting of (in mM) CaEGTA (2.8), EGTA (7.2), ATP (5.8), MgCl_2_ (6.6), taurine (20), phosphocreatine (15), imidazole (20), DTT (0.5), and MES (50) (pH 7.1). Tissue was washed in respiration solution containing EGTA (0.5), MgCl_2_ (3), K-lactobionate (60), taurine (20), KH_2_PO_4_ (10), HEPES (20), sucrose (110) and 1 g L^−1^ fatty acid-free BSA (pH 7.1), quickly blotted dry, weighed and transferred to a respirometer (Oxygraph-2k; Oroboros Instruments, Innsbruck, Austria) in respiration solution at 37 °C. Oxygen concentration was maintained above 300 μM throughout the experiment to avoid limitations in oxygen supply. Background respiration was assessed before adding substrates and was subtracted from all subsequent values. Leak respiration (L) was assessed after the addition of sodium glutamate (10 mM), sodium malate (0.5 mM), and sodium pyruvate (5 mM). NADH (Complex I)-linked respiration was assessed after the addition of 5 mM ADP and 10 μM cytochrome c to confirm the absence of outer-mitochondrial membrane damage. Maximal oxidative phosphorylation capacity, with simultaneous convergent electron input via NADH and FADH-linked pathways, was measured after the addition of 10 mM succinate. Maximum uncoupled respiration (E) was determined via titration of carbonylcyanide-4-trifluoro-methoxyphenylhydrazone (FCCP) in 0.5 µM steps until no further increase in oxygen consumption was observed. Succinate-linked respiration was measured after blocking mitochondrial complex I with 0.5 μM rotenone. Two measurements per sample were performed simultaneously, and the results were averaged. Respiration values were normalized to wet weight and expressed in pmol O_2_·s^−1^ mg^−1^. Samples that increased more than 10% from the addition of 5 mM of ADP to 10 μM cytochrome c were excluded from analysis. E/L coupling efficiency was calculated as the difference between maximum uncoupled respiration and leak respiration divided by the maximum uncoupled respiration ((E-L)/L). The phosphorylation capacity through complex I (normalized NADH-linked respiration) and complex II (normalized succinate-linked respiration) was calculated as Complex I-linked respiration normalized to maximal oxidative phosphorylation capacity and Complex II-linked respiration normalized to maximal uncoupled respiration, respectively.

### Immunohistochemistry

To visualize muscle fiber type and cross-sectional area, immunohistochemistry was carried out on 10-µm-thick sections from the vastus lateralis muscle biopsies. Details about dilutions and vendors can be found in Table 2.

**Table 2:** List of antibody vendors

Parameter	Primary antibody, dilution, vendor	Catalog #/RRID	Secondary antibody, dilution, vendor	Catalog #/RRID
**Muscle fiber type**	BA-D5, 1 µg/mL, DSHB	DSHB Cat# BA-D5, RRID:AB_2235587	Goat Anti-mouse IgG2B 555, 1:2000, Invitrogen	Thermo Fisher Scientific Cat# A-21147, RRID:AB_2535783
	6H1, 5 µg/mL, DSHB	DSHB Cat# 6H1, RRID:AB_1157897	Goat Anti-mouse IgM 488, 1:2000, Invitrogen	Thermo Fisher Scientific Cat# A-21042, RRID:AB_2535711
	SC-71 1 µg/mL, DSHB	DSHB Cat# SC-71, RRID:AB_2147165	Goat Anti-mouse IgG1 647,1:2000, Invitrogen	Thermo Fisher Scientific Cat# A-21240, RRID:AB_2535809
	WGA 350, 1:25, Invitrogen	W11263		
**Capillaries**	Ulex europaeus agglutinin I, 20 µg/mL, Vector Laboratories	Vector Laboratories Cat# B-1065, RRID:AB_2336766	Vectastain Elite ABC Kit PK-6100, Vector Laboratories	Vector Laboratories Cat# PK-6100, RRID:AB_2336819
			ImmPACT™ AMEC Red Peroxidase Substrate, Vector Laboratories	Vector Laboratories Cat# SK-4285, RRID:AB_2336519

### Fiber-type composition

Skeletal muscle fiber-type quantification and size were assessed using immunofluorescence techniques using primary antibodies against myosin heavy chain (MHC) I (BA-D5), MHC IIa (SC-71), and MHC IIx (6H1)^6^. Firstly, sections were air-dried for 10 min and then blocked with 10% normal goat serum (NGS) for 60 min. Subsequently, slides were washed 3 times for 5 min each in 1× phosphate-buffered saline (PBS) and incubated with the primary antibodies for 60 min at room temperature. Slides were washed and subsequently incubated with the secondary antibodies for 60 min in the dark at room temperature. Another washing step was performed, and subsequently, the slides were incubated with Wheat Germ Agglutinin (WGA) for 30 min in the dark at room temperature. This was followed by a final washing step before the sections were mounted with coverslips using Vectashield Vibrance. Analysis was performed with ImageJ and a modified version of Sandia Matlab AnalysiS Hierarchy (SMASH) Toolbox (version 1.0) in MATLAB (Version 2022a). Fiber-type composition was expressed as the cross-sectional area occupied by each fiber-type as a percentage of the cross-sectional area of all fibers. This allowed for quantification of fiber-type-specific fiber cross-sectional area.

### Succinate dehydrogenase activity

Sections were stained for SDH activity as described previously^6^. Immediately after cutting, sections were air-dried for 15 min and then immersed in a pre-heated (37 °C) solution containing sodium phosphate buffer (0.1 M, pH 7.6), sodium succinate (0.2 M), sodium azide (14 mM) and tetranitro blue tetrazolium (TNBT, 0.55 mM, Sigma-Aldrich) for 20 min in the dark, and the reaction was stopped by brief HCl (0.01 M) exposure before sections were washed and mounted with glycerin-gelatin. Sections were stored at 4 °C and imaged within ten days. The averaged SDH activity was obtained by outlining 40 individual skeletal muscle fibers per participant, and absorbance was assessed using ImageJ (Version 1.54f, NIH, Bethesda, USA). Values were expressed as Δ*A*_660_ per µm tissue thickness per second of staining time (Δ*A*_660_ µm^−1^ s^−1^).

### Capillarization

Capillarization was assessed by staining for Ulex Europaeus Agglutinin 1 lectin (UEA-1)^6^. Sections were air-dried for 10 min and fixed in ice-cold acetone (−20 °C) for 15 min. Subsequently, slides were washed 3 times for 2 min in PBS and blocked with 1% bovine serum albumin for 30 min. Afterward, slides were incubated with UEA-1 for 30 min at room temperature, followed by wash steps and incubation with Vectastain Elite ABC kit for 30 min at room temperature. After washing, incubation with Red Peroxidase substrate for 10 min at room temperature was performed, washed, and sections were mounted with glycerin-gelatin (heated at 37 °C). Capillary-to-fiber ratio and capillary density by capillaries per mm^2^ muscle tissue, and capillary cross-sectional area were determined in ImageJ. Where available, capillary images were overlain with fiber-type images to quantify fiber-specific capillary-to-fiber ratios, wherein a minimum of 20 type I and 20 type II fibers were analyzed per participant. Fibers were deemed either type I positive (type I) or type I negative (type II), and capillaries were summed as capillary divided by # of fiber contacts per capillary.

### Myoglobin content

To assess intracellular myoglobin content, muscle biopsy sections were freeze-dried and fixed with paraformaldehyde (158127, Sigma-Aldrich) at 70–75 °C for 60 min using a vapor-fixation technique^82^. Slides were fixed with 2.5% glutaraldehyde (G5882, Sigma-Aldrich) buffer for 10 min before a 60 min incubation in O-tolidine-containing solution. The O-tolidine-containing solution was prepared in 62 ml 50 mM Tris-80 mM KCl buffer containing 25 mg ortho-tolidine (T8533, Sigma-Aldrich), dissolved in 2 ml 95% ethanol, and 1.43 ml 70% tertiary-butyl-hydroperoxide (458139, Sigma-Aldrich). Incubations were performed at 50 °C. Sections were washed and mounted with glycerin-gelatin. Ninety randomly selected fibers, regardless of fiber type, were analyzed using Fiji^83^. Values were expressed as arbitrary units (AU).

### Image acquisition

Slides were dried overnight at 4 °C before imaging. Images for fiber type and capillarization analysis were taken at ×20 magnification with VS200 Research Slide Scanner (Olympus) using Slideview 5.0. SDH images were taken with a DMRB microscope (Leica, Wetzlar) using a CCD camera, calibrated gray filters, and an individual calibration curve at 660 nm. Myoglobin images were captured with ×20/0.4 objective using a CCD camera at 436 ± 9 nm absorbance. Approximately fifteen images were taken per sample, with 5 images per slide-section. All images were manually evaluated to exclude those containing out-of-focus areas, artifacts, and large areas of connective tissue. Image processing and analysis were conducted using ImageJ (version 1.53 u and later). Fiber-type composition was performed with ImageJ and Sandia Matlab AnalysiS Hierarchy (SMASH) Toolbox (version 1.0) in MATLAB (version 2022a).

### Statistical analysis

Distributions were assessed with histograms and Shapiro–Wilk tests. Parametric quantitative variables are presented as means ± standard deviation (SD), and non-parametric quantitative variables are presented as median and interquartile ranges (IQR; 25th and 75th percentiles). Jitter plots are plotted with means represented as single bars. All individual points represent a participant.

Non-normally distributed data were transformed using the Box-Cox transformation in order to obtain a normal distribution for statistical analysis. Continuous parametric data were analyzed using a 2-tailed paired *t*-test or analysis of variance, with Tukey HSD post-hoc testing, where appropriate. If data remained non-normally distributed following the Box-Cox transformation, the continuous non-parametric data were analyzed using the Mann–Whitney *U* test, Kruskal–Wallis *H* test, or pairwise Wilcoxon test with Benjamini–Hochberg correction where appropriate. Correlations were analyzed using the Pearson correlation coefficient for linear relationships. Correlation coefficients were compared using the R package *cocor*. Pertinent to bed rest data, if data was missing in either the before or after bed rest condition, the participant was removed from the analysis to maintain equal sample sizes and appropriate pairing. All data were analyzed using RStudio built under R version 4.0.3 (R Core Team 2013, Vienna, Austria).

### Reuse of previously published data

Some results from bed rest participants were reanalyzed from previously published data^19,75^. Biopsies were stained and analyzed for capillarization, muscle fiber type, and myoglobin content to account for differences in staining protocols and analysis procedures. A part of the results from patients with long COVID and 21 healthy controls were previously published^6^.

### Reporting summary

Further information on research design is available in the Nature Portfolio Reporting Summary linked to this article.

## Supplementary information

Supplementary Information

Peer Review file

Reporting Summary

## Source data

Source data

## Data Availability

The processed data generated in this study are publicly available via the GitHub repository (doi: 10.5281/zenodo.20642303)^84^. Any raw data that underlies the source data is freely available from the corresponding author. The source data file is available in the supplemental files. Source data can also be accessed via the GitHub repository: https://github.com/bcn95/LC_ME_CFS_BED_REST. Source data are provided with this paper.

## Code availability

R codes are available in the supplemental files and via the GitHub repository^84^. All codes and a ReadMe file can be found at https://github.com/bcn95/LC_ME_CFS_BED_REST.
