# Supplementary material for: Skeletal muscle properties in long COVID and ME/CFS differ from those induced by bed rest
Source: Nat Commun. 2026 Jul 28;17:9125. doi: 10.1038/s41467-026-75725-y (PMC13518852; doi:10.1038/s41467-026-75725-y)
Supplement: Supplementary file 1 [file 41467_2026_75725_MOESM1_ESM.pdf]

Supplemental file to: Skeletal muscle properties in Long COVID and ME/CFS differ from those induced by bed rest

Braeden T. Charlton[1], Anouk Slaghekke[1], Brent Appelman[2,3*], Moritz Eggelbusch[1,4], Jelle Y. Huijts[1], Wendy Noort[1], Paul W. Hendrickse[5], Frank W. Bloemers[6], Jelle J. Posthuma[7], Paul van Amstel[8], Richie P. Goulding[1], Hans Degens[9,10], Richard T. Jaspers[1], Michèle van Vugt[3#], Rob C.I. Wüst[1#]*

*,#: equal contribution

[1] Department of Human Movement Sciences, Faculty of Behavioural and Movement Sciences, Amsterdam Movement Sciences, Vrije Universiteit Amsterdam, Amsterdam, Netherlands

[2] Center for Infection and Molecular Medicine, Amsterdam University Medical Centre, Amsterdam, Netherlands

[3] Amsterdam Institute of Infectious Disease, Amsterdam University Medical Centre, Amsterdam, Netherlands

[4] Professorship of Exercise Biology, TUM School of Medicine and Health, Technical University of Munich, Munich, Germany

[5] Faculty of Health and Medicine, Lancaster University, Lancaster, United Kingdom

[6] Department of Trauma Surgery, Amsterdam University Medical Centre, Amsterdam, Netherlands

[7] Department of Trauma Surgery, Flevoziekenhuis Almere, Almere, Netherlands

[8] Department of Pediatric Surgery, Amsterdam University Medical Centre, Amsterdam, Netherlands

[9] Department of Life Sciences, Manchester Metropolitan University, Manchester, United Kingdom

[10] Lithuanian Sports University, Institute of Sport Science and Innovations, Kaunas, Lithuania

Supplemental Table 1. Sub-cohort characteristics with both cohorts now matched for age and sex. Data presented as median (IQR). Pre-bed rest participants were shorter than healthy, patient-matched controls (p=0.038). Patients with long COVID had experienced symptoms for a shorter time-period than patients with myalgic encephalomyelitis/chronic fatigue syndrome (ME/CFS, p<0.001). All data remained non-normally distributed following Box-Cox transformations. Group differences between pre-bed rest and healthy, patient-matched controls were assessed using Group differences between post-bed rest, long COVID and ME/CFS were compared using a Kruskal-Wallis test, with pairwise Wilcoxon signed-rank test post-hoc where applicable. Differences between patients with long COVID and patients with ME/CFS in symptom duration and daily steps were assessed using Wilcoxon signed-rank tests.

	**Bed rest**	**Healthy Control**	**Bed rest**	**Long COVID**	**ME/CFS**
	**Pre**		**Post**		
**Participants (# Females)**	17 (7)	17 (7)	13 (7)	13 (7)	13 (7)
**Age (years)**	34 (29-43)	34 (29-42)	29 (27-40)	31 (28-42)	32 (28-42)
**Height (cm)**	175 (172-179) *	180 (176-186)	174 (162-175)	177 (168-191)	175 (172-186)
**Weight (kg)**	76 (69-80)	74 (63-86)	70 (66-76)	82 (71-90)	84 (75- 86)
**Symptom Duration (days)**	n/a	n/a	n/a	500 (408-599) †††	3793 (2463-4613)
**Daily Steps (steps.day^{-1})**	ND	7011 (4770-8316)	0 (0 0)	3970 (2794-5030)	3827 (2722-4901)

***: *vs.* healthy control; †: *vs.* ME/CFS; §: *vs.* Bed rest**

***: *p*<0.05; **: *p*<0.01; ***: *p*<0.001**

Supplemental Table 2. Primary and secondary Endpoints for exercise testing. Data pertaining to primary and secondary endpoint criteria of cardiopulmonary exercise tests are summarized below. Across 129 tests, 40 tests did not meet any of the primary endpoint criteria, while only 7 tests failed to meet any primary or secondary endpoint criteria. Lactate values were not collected during the bed rest study.

**Primary Endpoints**	**Before Bed rest (n=24)**	**After Bed rest (n=24)**	**CON (n=30)**	**LC (n=25)**	**ME/CFS (n=26)**
Final 2min $\dot{V}O_2$ slope <100ml/min (n)	4	3	5	10	10
Final 2min $\dot{V}O_2$ Slope >50% difference (n)	1	9	7	13	7
Points outside 95% CI in final 2min (n)	11	7	17	11	7
**Secondary Endpoints**					
Achieved Adjusted Max Heart Rate (n)	2	9	13	8	4
Achieved blood lactate >8mM (n)	ND	ND	24	19	11
Achieved RER >1.15 (n)	19	18	20	16	8
Number of participants that failed to meet any endpoint criteria (n)	3	1	0	0	3

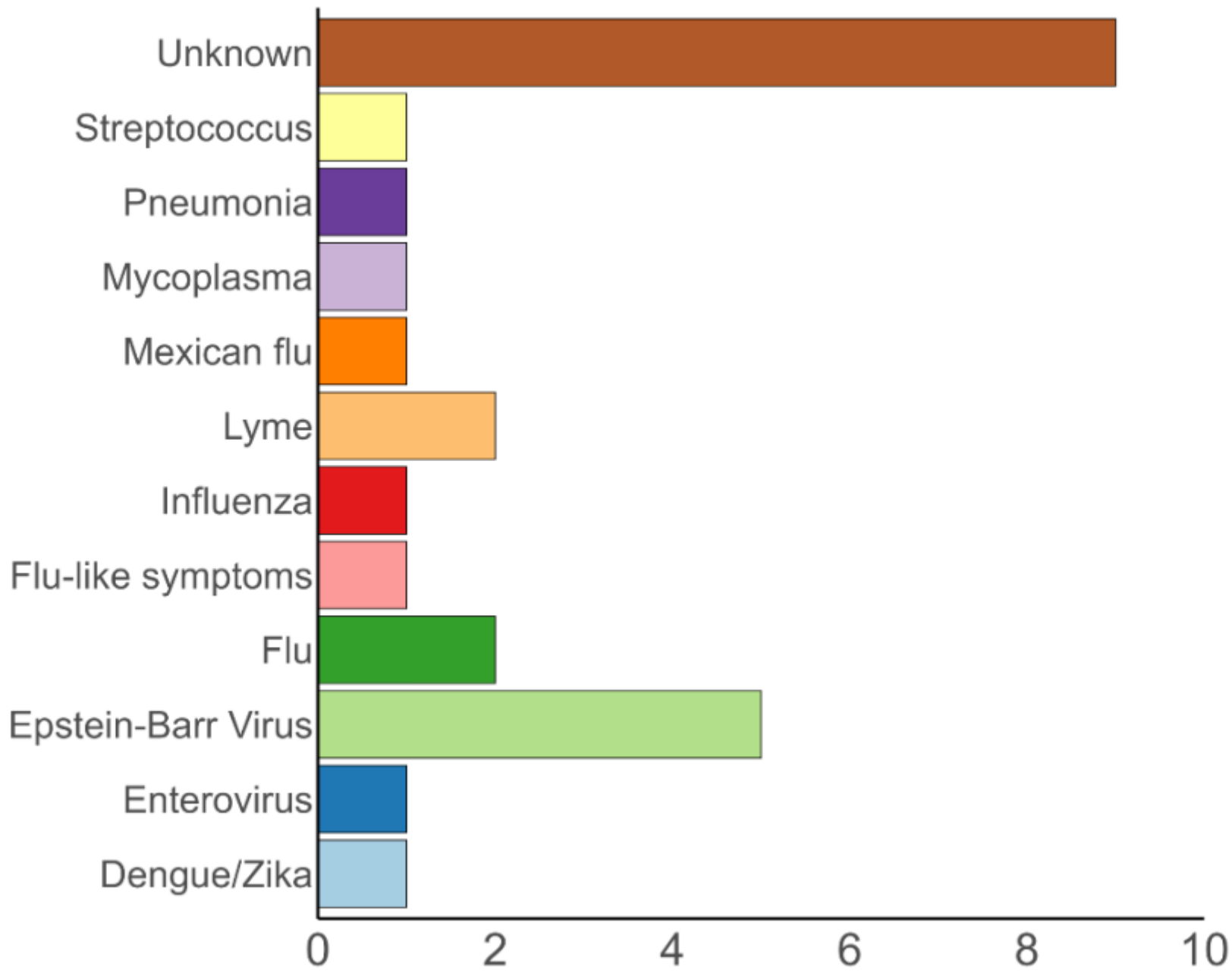

Supplemental Figure 1: **Patients with ME/CFS report a range of infections prior to disease onset.** Infection type prior to myalgic encephalomyelitis/chronic fatigue syndrome (ME/CFS) diagnosis, based on responses from participants and not necessarily clinically confirmed. All ME/CFS diagnoses (n=26) were made prior to 2020. Bars display the total number of responses.

A

		CON	LC	ME/CFS
Frequency	DSQ-PEM1	0 (0,1)$^{***, \dagger\dagger\dagger}$	4 (1,4)**	4 (2,4)
	DSQ-PEM2	0 (0,1)$^{***, \dagger\dagger\dagger}$	3 (0,4)	3.5 (1,4)
	DSQ-PEM3	0 (0,0)$^{***, \dagger\dagger\dagger}$	3 (2,4)	3 (1,4)
	DSQ-PEM4	0 (0,0)$^{***, \dagger\dagger\dagger}$	3 (0,4)*	3 (1,4)
	DSQ-PEM5	0 (0,0)$^{***, \dagger\dagger\dagger}$	3 (1,4)	3 (2,4)
Severity	DSQ-PEM1	0 (0,1)$^{***, \dagger\dagger\dagger}$	3 (1,4)***	3 (3,4)
	DSQ-PEM2	0 (0,1)$^{***, \dagger\dagger\dagger}$	2 (0,3)**	3 (1,4)
	DSQ-PEM3	0 (0,0)$^{***, \dagger\dagger\dagger}$	3 (1,4)	3 (1,4)
	DSQ-PEM4	0 (0,0)$^{***, \dagger\dagger\dagger}$	2 (0,4)**	3 (1,4)
	DSQ-PEM5	0 (0,0)$^{***, \dagger\dagger\dagger}$	3 (1,4)*	3 (2,4)

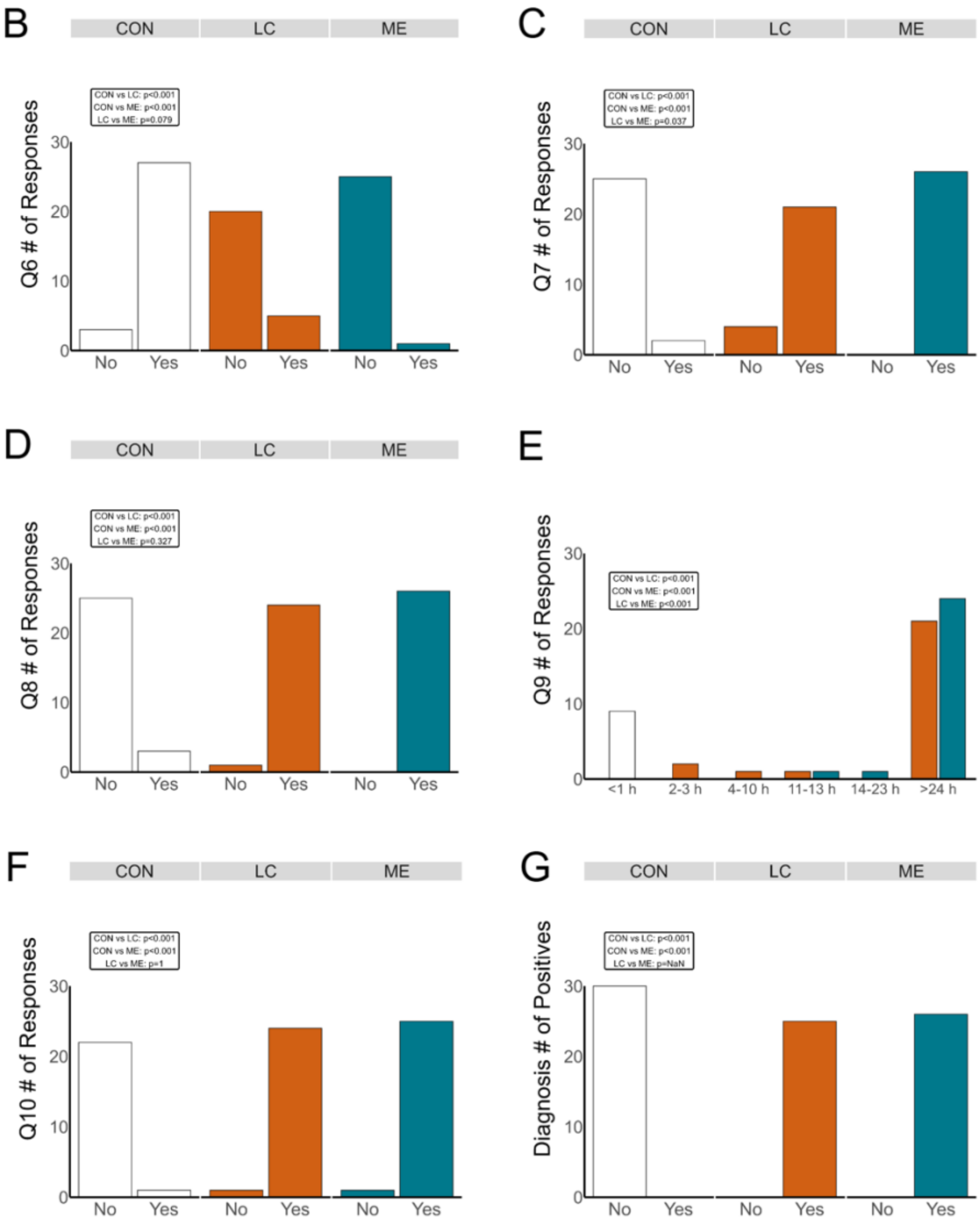

Supplemental Figure 2: **The DSQ-PEM distinguishes both patients with long COVID and ME/CFS from healthy controls.** DePaul Symptom Questionnaire-Post-exertional Malaise (DSQ-PEM) responses. Patients with long COVID (LC, n=25) and myalgic encephalomyelitis/chronic fatigue syndrome (ME/CFS; ME, n=26) reported significantly higher DSQ-PEM severity and frequency scores (**A**) compared to healthy controls (n=30). Patients with ME/CFS scored higher than patients with long COVID in DSQ-PEM F1 (p=0.024), DSQ-PEM F4 (p=0.028), DSQ-PEM S1 (p<0.001, DSQ-PEM S2 (p=0.008), DSQ-PEM S4, (p=0.009) and DSQ-PEM S5 (p=0.018). Patients with long COVID and ME/CFS responded more negatively to questions 6-8 and 10 of the DSQ-PEM (**B-D,F**) and indicated longer durations of feeling worse following activities (**E**) compared to healthy controls. All patients with long COVID and ME/CFS had a positive PEM diagnosis using the DSQ-PEM (**G**), while no controls were positive for PEM. Responses in **A** are displayed as median (minimum, maximum). Comparisons were made using a Kruskal–Wallis H test, with pairwise Wilcoxon tests with Benjamini–Hochberg correction post-hoc. *p<0.05, **p<0.01, ***p<0.001. *Compared to ME/CFS, †Compared to long COVID. Patients with long COVID are displayed in orange, and patients with ME/CFS are displayed in green.

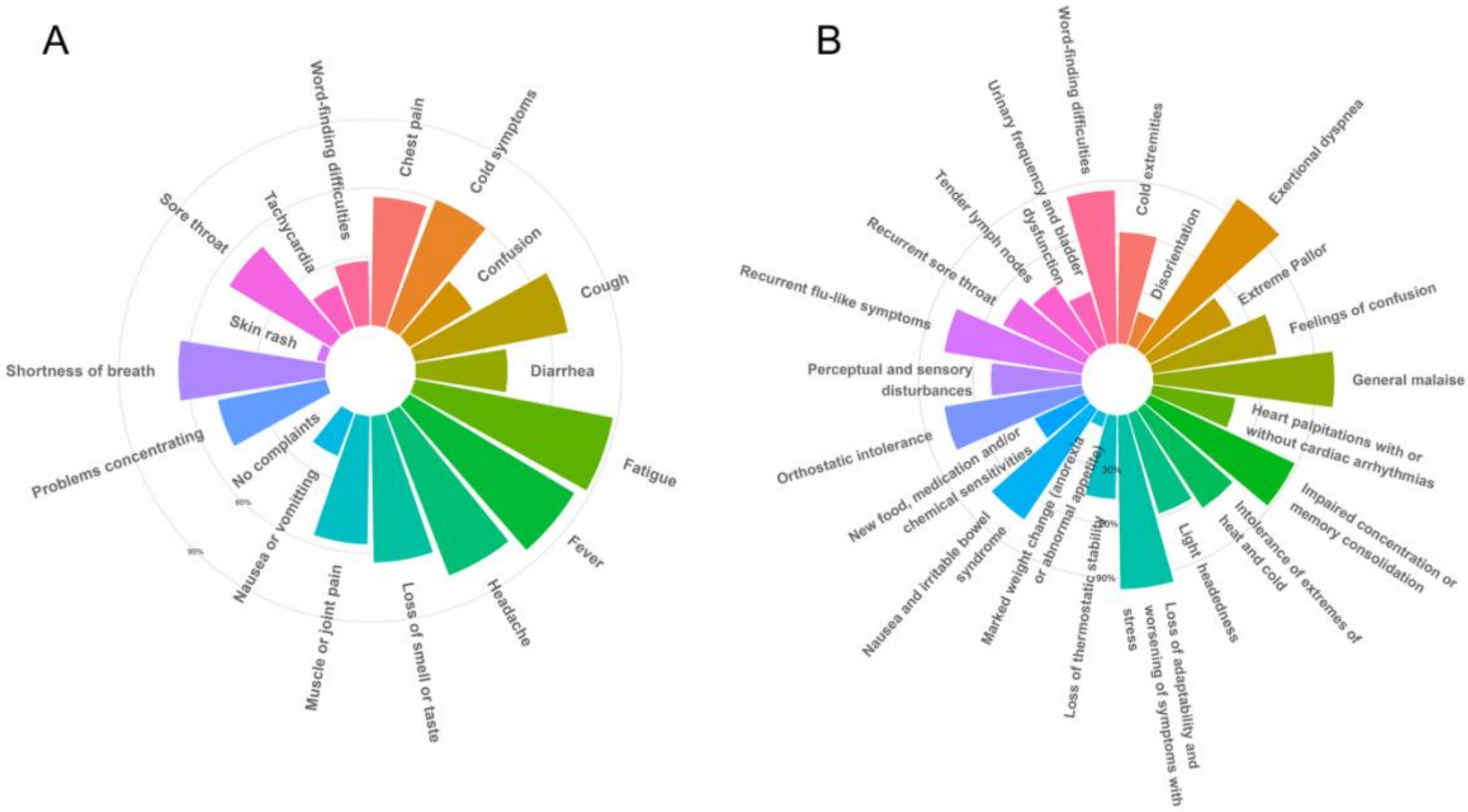

Supplemental Figure 3. **Patients with long COVID and ME/CFS report a range of symptoms.** Circular bar plots of symptoms reported by patients with long COVID (**A**, n=25) and myalgic encephalomyelitis/chronic fatigue syndrome (n=26, **B**). Bars represent percentage of patients indicating positively for the given symptom.

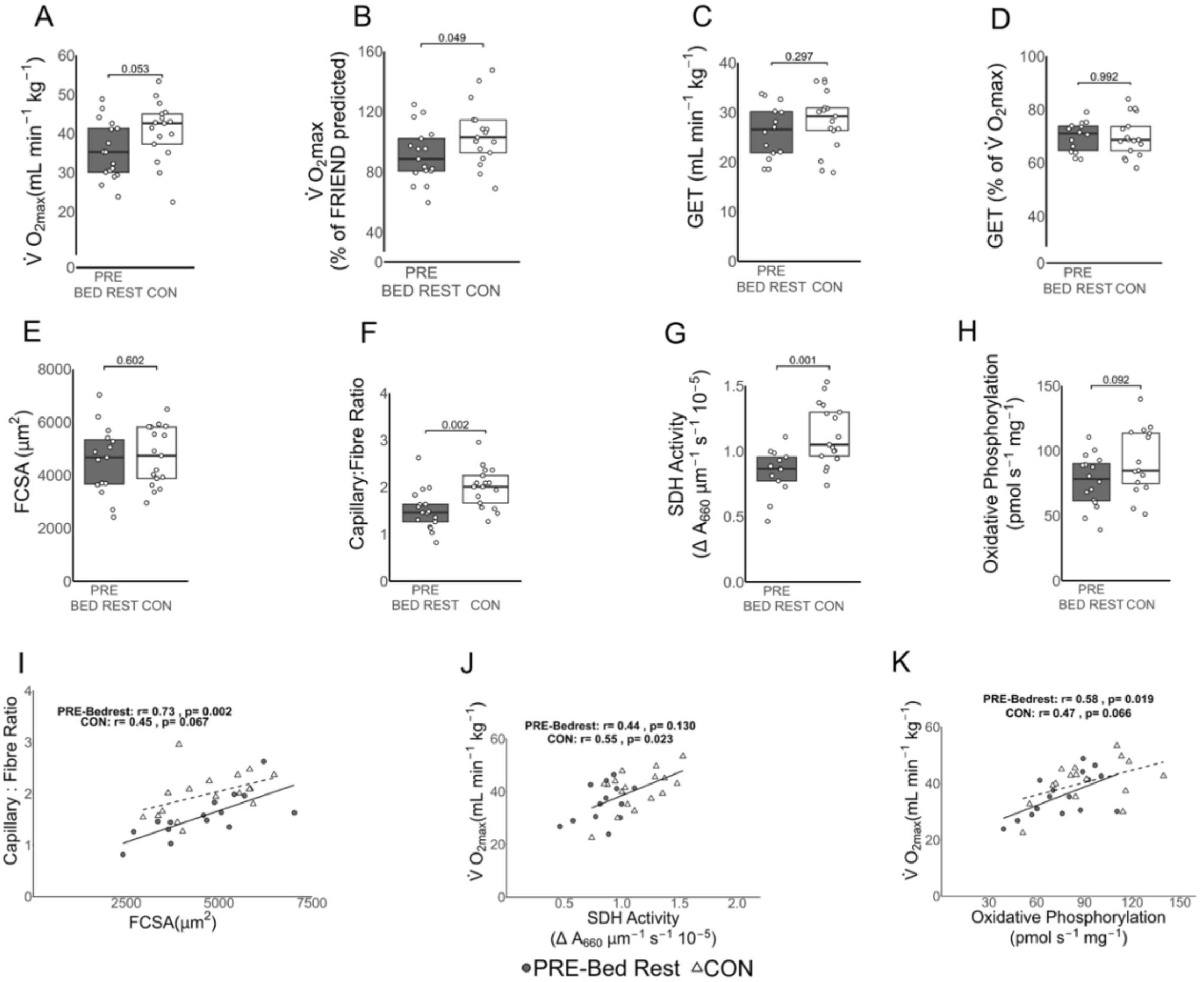

Supplemental Figure 4. **Healthy bed rest participants tend to have lower oxidative capacity than patient-matched healthy controls.** A sub-analysis was conducted on age- and sex-matched individuals between healthy individuals pre-bed rest (n=17) and healthy individuals matched to patients with long COVID and myalgic encephalomyelitis/chronic fatigue syndrome (CON, n=17; Supplemental Table 1). There was a tendency for $\dot{V}O_{2max}$ to be lower in bed rest participants compared to the patient-matched healthy controls (**A,** *p*=0.053) when expressed relative to body weight, and a significant difference when expressed as % of predicted $\dot{V}O_{2max}$ based on the FRIEND equation (**B**). However, there were no differences in in gas exchange threshold (GET) (**C**), the occurrence of the gas exchange threshold relative to $\dot{V}O_{2max}$ (**D**) or fiber cross sectional area (FCSA, **E**) between groups. Participants before bed rest had a lower capillary-to-fiber ratio (**F**), lower succinate dehydrogenase (SDH) activity (**G**), and oxidative phosphorylation capacity (**H**) than patient-matched healthy controls. Both groups had, or tended to have a significant relation between FCSA and capillary-to-fiber ratio (**I**). Only patient-matched healthy controls had a significant relation between $\dot{V}O_{2max}$ and SDH activity (**J**). Both groups had, or tended to have, a significant association between $\dot{V}O_{2max}$ and oxidative phosphorylation capacity (**K**). Data for $\dot{V}O_{2max}$, FCSA, and SDH activity remained non-normally distributed after Box-Cox transformations. Parametric comparisons were assessed using non-paired two-sided t-tests. Non-parametric comparisons were assessed using two-sided Wilcoxon signed rank tests. Solid lines represent significant correlations, dashed lines represent correlations with $p<0.10$. Box plots display individual data points, median (center line) and the first and third quartiles (lower and upper bound of box). Pre-bed rest and healthy controls are displayed in white, post-bed rest is displayed in grey, patients with long COVID are displayed in orange, and patients with ME/CFS are displayed in green.

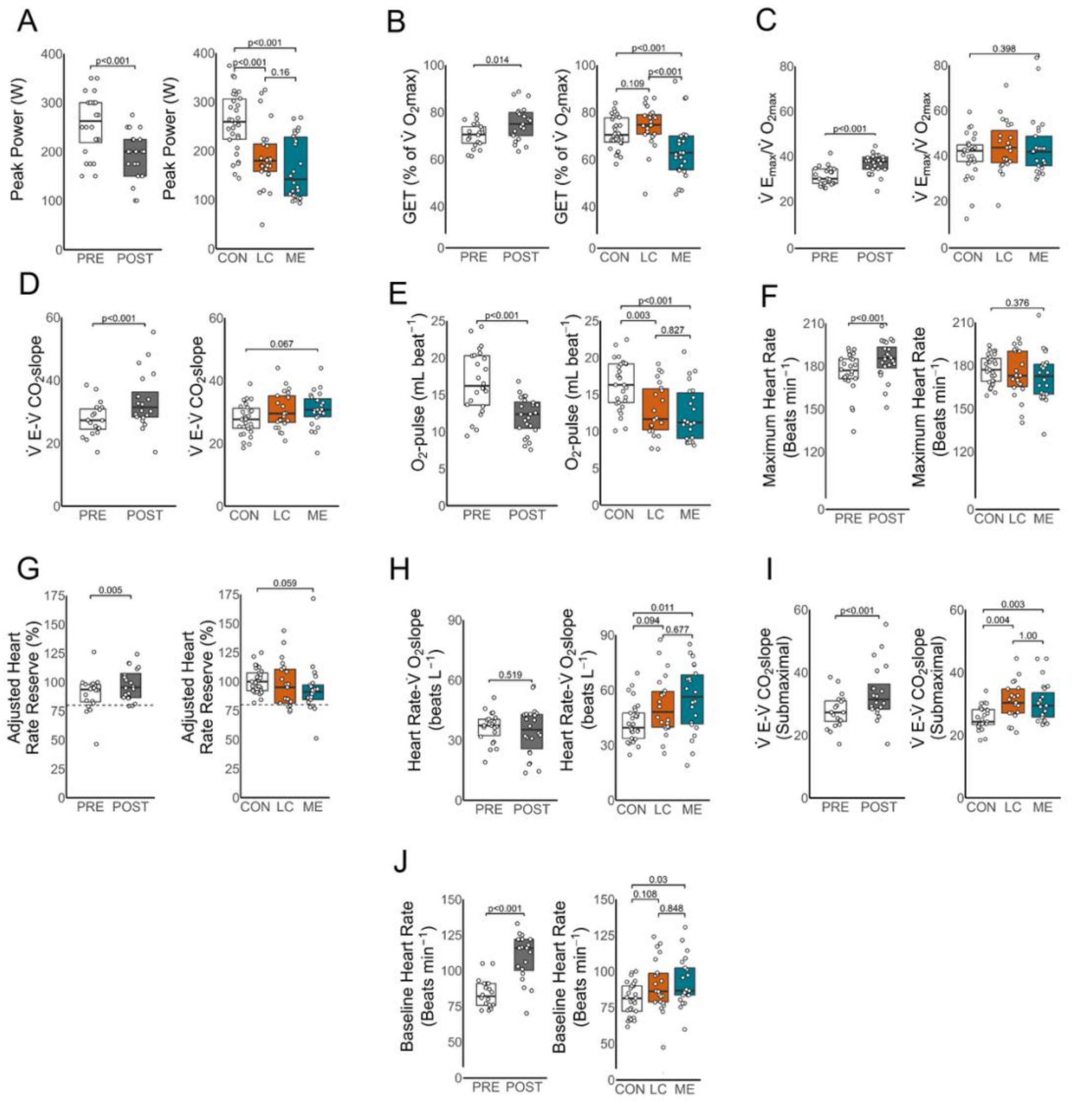

Supplementary Figure 5. **Bed rest results in alterations in respiratory and cardiovascular responses to maximal exercise, which are not seen in either patients with long COVID or ME/CFS.** Cardiopulmonary exercise test (CPET) data from pre- and post-bed rest, healthy controls (CON), patients with long COVID (LC) and patients with myalgic encephalomyelitis/chronic fatigue syndrome (ME/CFS; ME). Peak power was reduced following bed rest (**A**; n=24), and was also lower in LC and ME/CFS. Following bed rest, $\dot{V}E_{max}/\dot{V}CO_{2max}$ (**B**, n=24), $\dot{V}E_{max}/\dot{V}O_{2max}$ (**C**; n=24), and $\dot{V}E/\dot{V}CO_2$ slope (**D**; n=20) all increased, whereas LC and ME/CFS were not significantly different from CON. O_2-pulse was significantly lower following bed rest (**E**; n=24), and lower in LC (n=22) and ME/CFS (n=22) compared to CON (n=29). Maximal heart rate was increased following bed rest (n=24), while LC (n=22) and ME/CFS (n=22) had similar maximal heart rates to CON (n=22; **F**). Following bed rest (n=20), adjusted heart rate reserve (AHRR) was significantly higher, but ME/CFS (n=21) had the tendency have a lower AHRR compared to CON (n=29; **G**). The $\dot{V}O_2$-heart rate slope was not altered following bed rest (n=22), while both LC (n=22) and ME/CFS (n=22) had, or tended to have, a higher $\dot{V}O_2$-heart rate slope compared to CON (n=29; **H**). After bed rest (n=21), the gas exchange threshold (GET) occurred at a higher percentage of $\dot{V}O_{2max}$, whereas the GET occurred at a lower percentage of $\dot{V}O_{2max}$ in ME/CFS (n=22) compared to both CON (n=30) and LC (n=23; **I**). Submaximal $\dot{V}E/\dot{V}CO_2$ slope was increased following bed rest (n=20), and also increased in both LC (n=21) and ME/CFS (n=23) compared to CON (n=27; **J**). Baseline heart rates

were elevated following bed rest (n=20; **K**), and tended to be higher in LC (n=22) and ME/CFS (n=21) than CON (n=29). Comparisons between pre- and post- bed rest were assessed using paired t-tests for parametric data and Mann–Whitney *U* test for non-parametric data. Comparisons between LC, ME/CFS and healthy controls were assessed using ANOVA, with Tukey HSD post-hoc testing for parametric data or Kruskal–Wallis *H* test, with pairwise Wilcoxon tests with Benjamini–Hochberg correction post-hoc for non-parametric data. Box plots display individual data points, median (center line) and the first and third quartiles (lower and upper bound of box).

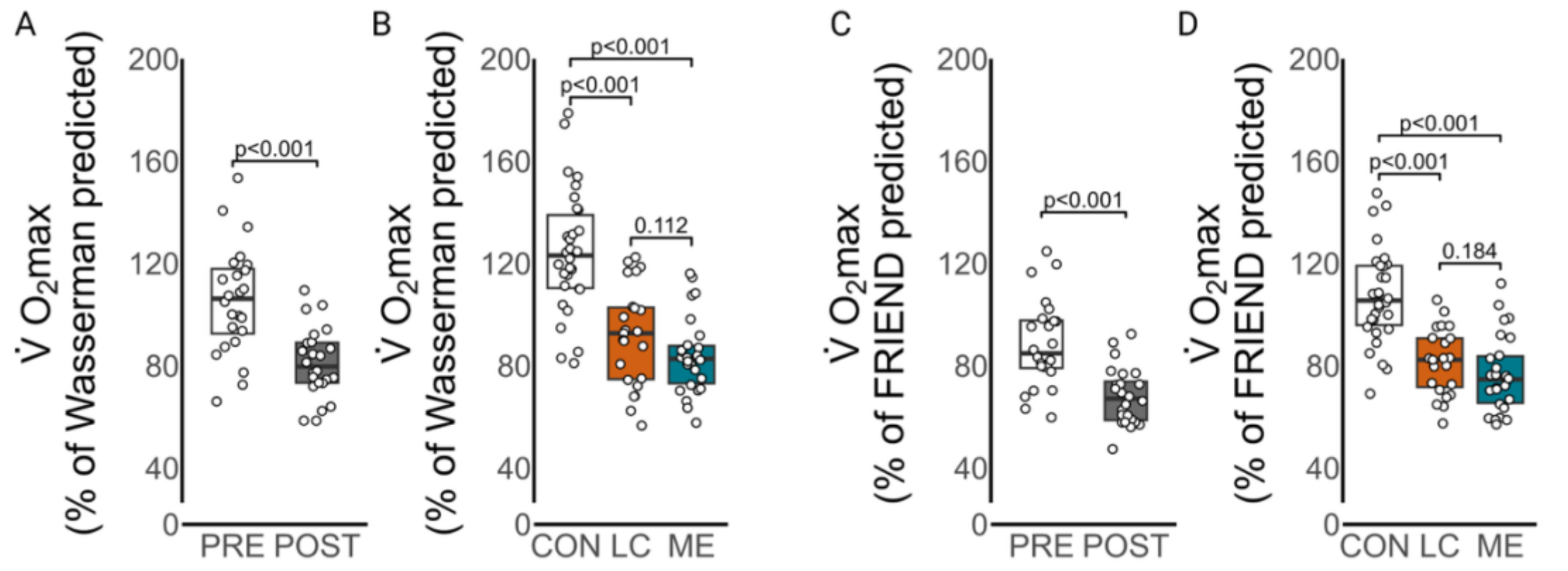

Supplemental Figure 6. **Lower $\dot{V}O_{2max}$ still exists after bed rest and in both patients with long COVID and ME/CFS after correcting for predicted $\dot{V}O_{2max}$.** Maximal oxygen consumption ($\dot{V}O_{2max}$) presented as a percentage of predicted $\dot{V}O_{2max}$, using the Wasserman (**A,B**) and the FRIEND equation (**C,D**). After Box-Cox transformations, only data from bed rest participants calculated with the Wasserman equation were normally distributed, all others remained non-normally distributed. Comparisons between pre- and post- bed rest (n=24) were assessed using paired t-tests for parametric data and Mann–Whitney *U* test for non-parametric data. Comparisons between patients with long COVID (LC, n=23) and patients with myalgic encephalomyelitis/chronic fatigue syndrome (ME/CFS; ME, n=26) and healthy controls (CON, n=30) were assessed using a Kruskal–Wallis *H* test, with pairwise Wilcoxon tests with Benjamini–Hochberg correction post-hoc for non-parametric data. Box plots display individual data points, median (center line) and the first and third quartiles (lower and upper bound of box). Pre-bed rest and healthy controls are displayed in white, post-bed rest is displayed in grey, patients with long COVID are displayed in orange, and patients with ME/CFS are displayed in green.

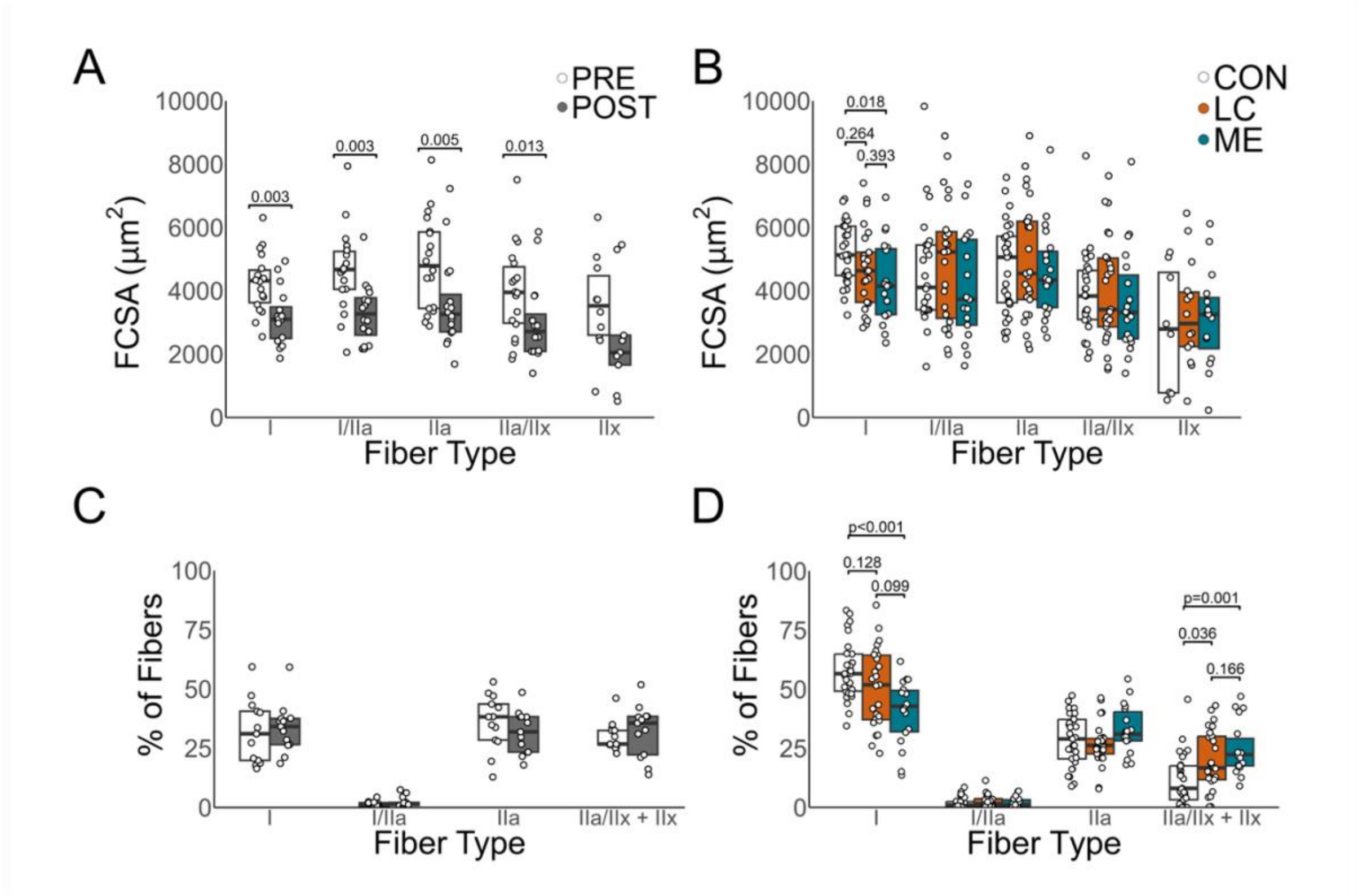

Supplementary Figure 7. **Bed rest induces atrophy across all muscle fiber types, while patients with ME/CFS have a type-I specific atrophy.** Average fiber cross-sectional area (FCSA) for each individual fiber type was reduced from pre- to post-bed rest (n=13; **A**), with the exception of the few pure type IIx fibers (*p*=0.329). Type I fiber FCSA was significantly lower in patients with myalgic encephalomyelitis (ME/CFS; ME; ANOVA: *p*=0.022, post-hoc: *p*=0.018, n=19; **B**) compared to healthy controls (CON, n=29), while long COVID patients (LC; *p*=0.393, n=25) were not significantly different from controls. Other fiber types were not significantly different in either patient group compared to healthy controls (ANOVA/ Kruskal-Wallis: I/IIa *p*=0.410, IIa *p*=0.847, IIa/IIx *p*=0.683, IIx *p*=0.817). Fiber type was subsequently calculated as the numerical percentage relative to total fiber number (**C**,**D**). Bed rest did not change fiber type distribution (**C**; Linear mixed model: *p*=0.629). ME/CFS patients had less type I fibers compared to controls (*p*<0.001) and tended to have less than long COVID patients (**D**; *p*=0.099). Both long COVID and ME/CFS patients had more type IIa/IIx hybrid fibers than controls. After Box-Cox transformations, FCSA data of type I, type I/IIa and IIa fibers were normally distributed, while FCSA data of type IIa/IIx and IIx fibers remained non-normally distributed. Data for percentage of type I in bed rest, percentage of I/II hybrids in all groups, and percentage of IIa fibers in all groups were normally distributed after Box-Cox transformations; all other data was non-normally distributed. Fiber type proportions were assessed using a linear mixed random effects model using % of proportion as the dependent variable and group*fiber type as the fixed effects, with subject ID as the random effects with post-hoc testing carried out per fiber type as described below. Comparisons between pre- and post- bed rest were assessed using paired t-tests for parametric data and Mann–Whitney *U* test for non-parametric data. Comparisons long COVID patients, ME/CFS patients, and controls were assessed using ANOVA, with Tukey HSD post-hoc testing for parametric data or Kruskal–Wallis *H* test, with pairwise Wilcoxon tests with Benjamini–Hochberg correction post-hoc for non-parametric data. Box plots display individual data points, median (center line) and the first and third quartiles (lower and upper bound of box).

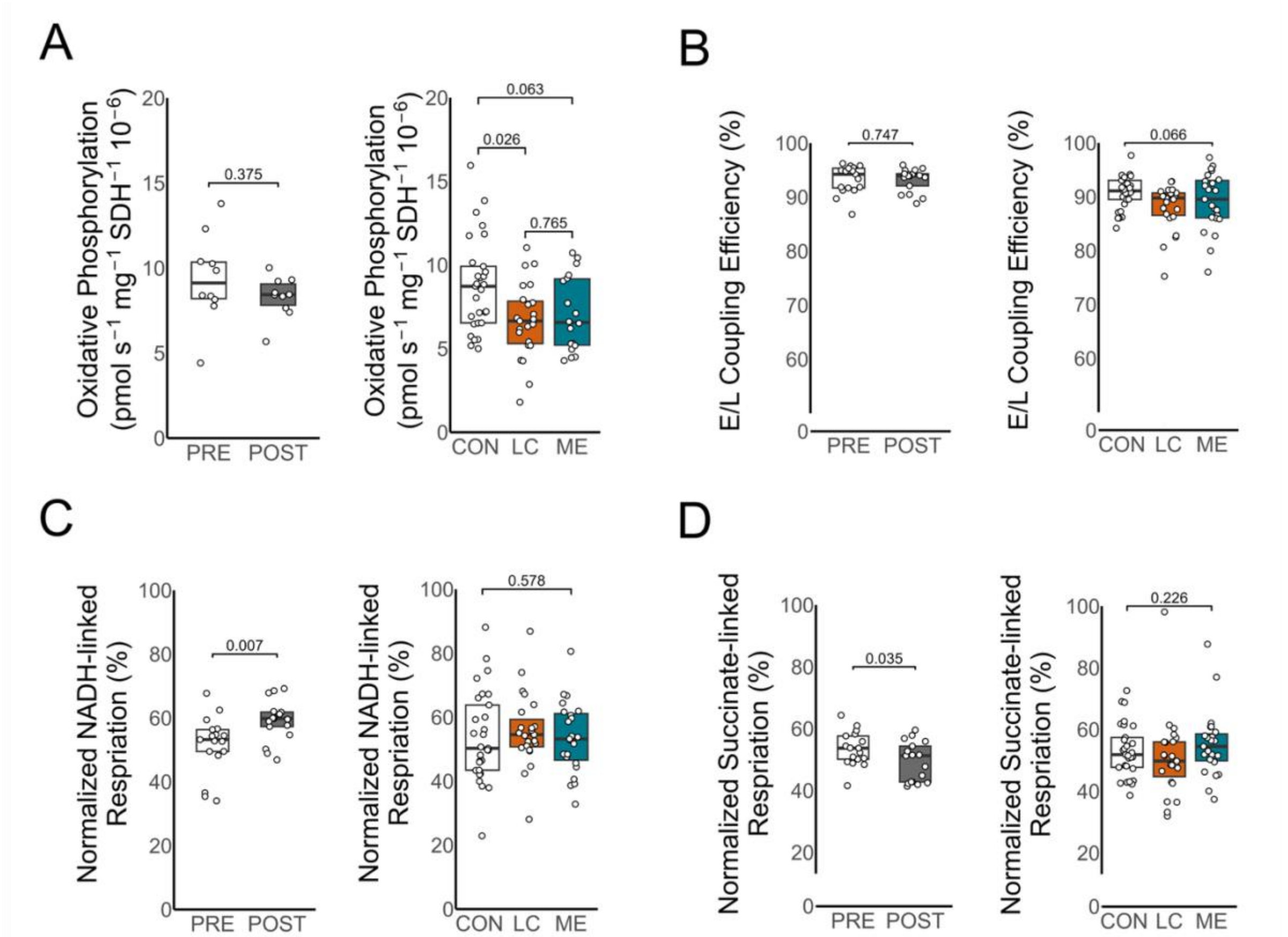

Supplementary Figure 8. **Bed rest does not affect intrinsic mitochondrial respiration, while both patients with long COVID and ME/CFS have reduced intrinsic mitochondrial capacity.** Oxidative phosphorylation capacity was normalized to succinate dehydrogenase (SDH) activity to provide a marker for intrinsic mitochondrial respiration. Bed rest (n=10) did not alter the normalized oxidative phosphorylation capacity, while patients with long COVID (LC, n=23) and myalgic encephalomyelitis/chronic fatigue (ME/CFS; ME, n=18) displayed lower capacities than healthy controls (CON, n=29, **A**). Bed rest (n=17) did not alter biochemical coupling (uncoupled/leak (E/L) coupling efficiency), while both patients with long COVID (n=23) and ME/CFS (n=23) tended to have lower biochemical coupling compared to healthy controls (Kruskal-Wallis: p=0.066, n=29, **B**). Bed rest (n=17) increased the normalized NADH-linked respiration (NADH-linked respiration normalized to oxidative phosphorylation capacity; **C**) and decreased the normalized succinate-linked respiration (rotenone-linked respiration normalized to electron transport capacity; **D**), whereas neither patients with long COVID (n=23) nor ME/CFS (n=23) were not different from healthy controls (n=29, NADH-linked normalized Kruskal-Wallis: p=0.578, Succinate-linked normalized Kruskal-Wallis: p=0.226). After Box-Cox transformations, all data remained non-normally distributed. Data from bed rest participants were compared using a paired Wilcoxon Signed Rank test. Data from patients with long COVID and ME/CFS and healthy controls were compared using a Kruskal-Wallis H test, with pairwise Wilcoxon tests with Benjamini–Hochberg correction post-hoc where applicable. Box plots display individual data points, median (center line) and the first and third quartiles (lower and upper bound of box). Pre-bed rest and healthy controls are displayed in white, post-bed rest is displayed in grey, patients with long COVID are displayed in orange, and patients with ME/CFS are displayed in green.

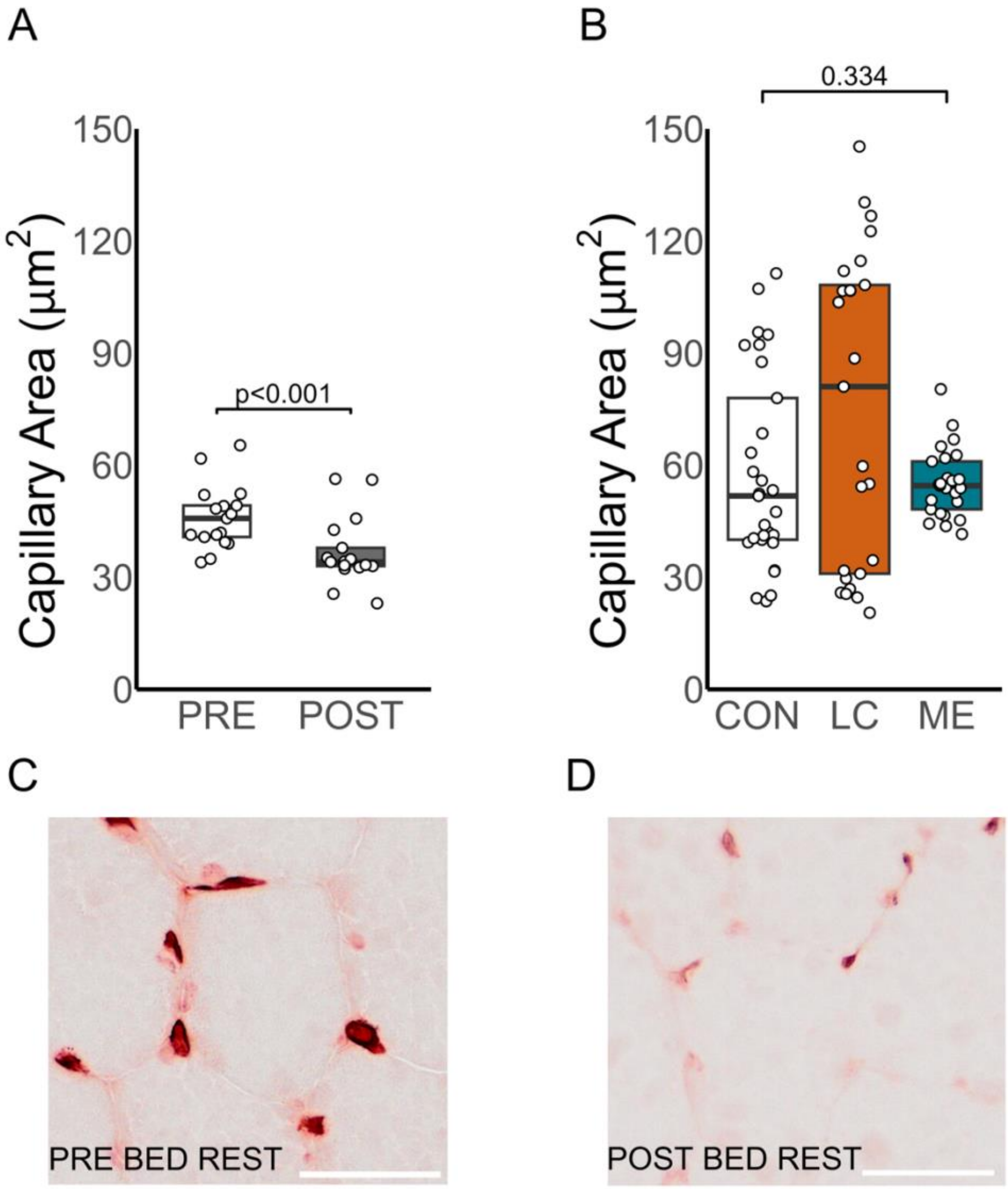

Supplementary Figure 9. **Bed rest induces a reduction in capillary area, while neither patient group is different to healthy controls.** Average capillary area decreased significantly following bed rest (n=17, **A**). Capillary area was not different between healthy controls (CON, n=29) and patients with myalgic encephalomyelitis/chronic fatigue syndrome (ME/CFS; ME, n=25, Kruskal-Wallis: p=0.334) or long COVID (LC, n=25; **B**). Typical examples of capillary staining in bed rest are shown in **C**. After Box-Cox transformations, capillary area data from bed rest participants was normally distributed, but data from patients with ME/CFS and long COVID and healthy controls remained non-normally distributed. Data from patients with ME/CFS and long COVID and healthy controls were compared using a Kruskal-Wallis H test. Box plots display individual data points, median (center line) and the first and third quartiles (lower and upper bound of box). Pre-bed rest and healthy controls are displayed in white, post-bed rest is displayed in grey, patients with long COVID are displayed in orange, and patients with ME/CFS are displayed in green. Scale bars represent 100μm.

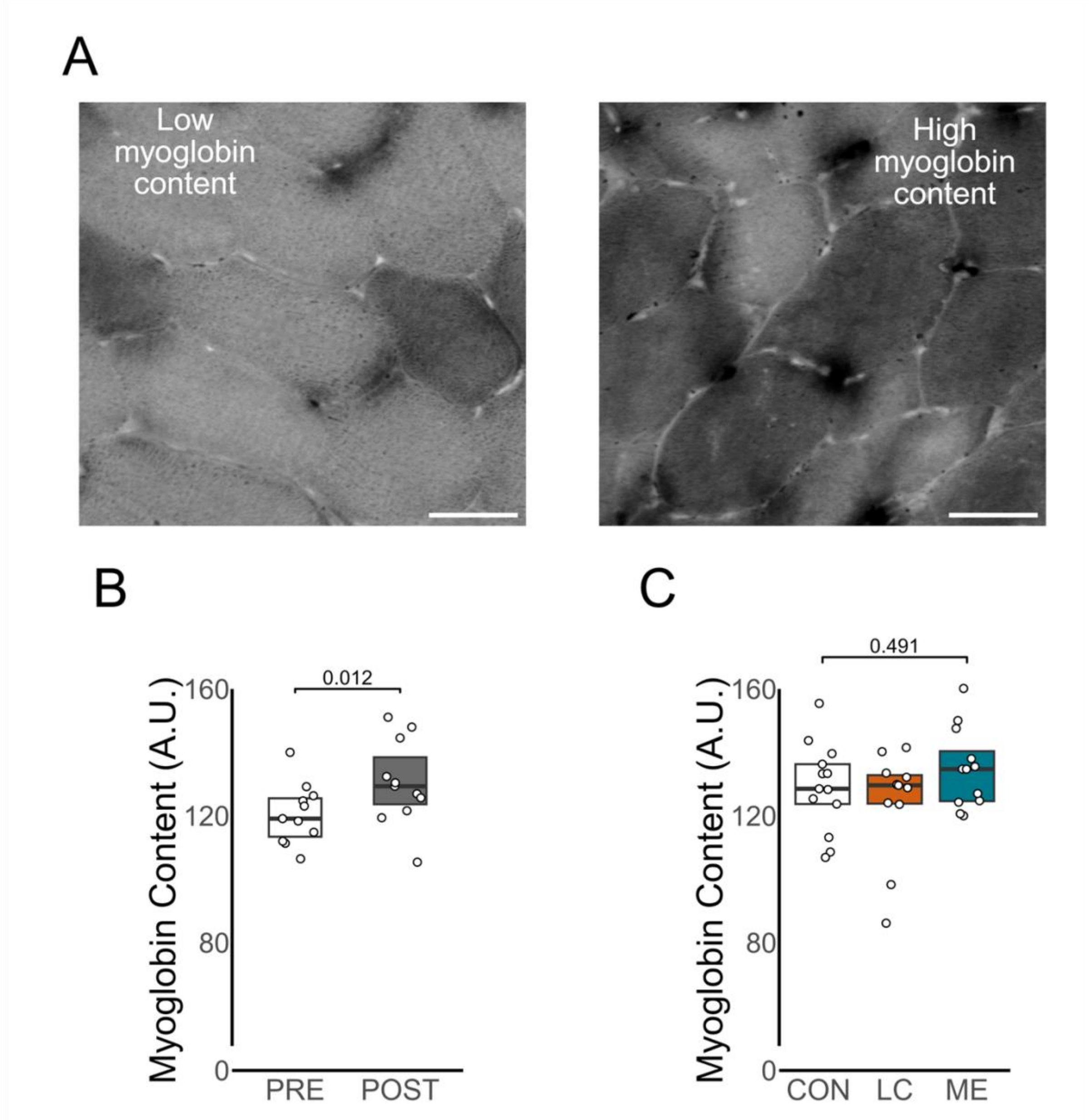

Supplementary Figure 10. **Myoglobin increases following bed rest, while neither patients with long COVID nor ME/CFS are different to healthy controls.** Typical examples of low myoglobin content and high myoglobin content are displayed (**A**). Myoglobin content was measured in a subset of participants. Following bed rest myoglobin content was significantly elevated (**B**; n=11), while no differences were found between healthy controls (**C**; n=13), patients with long COVID (LC, n=11), and patients with myalgic encephalomyelitis/chronic fatigue syndrome (ME/CFS; ME, n=12). Scale bar represents 50 µm. After Box-Cox transformations, myoglobin data from bed rest participants was normally distributed, but data from patients with ME/CFS and long COVID and healthy controls remained non-normally distributed. Data from bed rest participants were compared using a paired t-test. Data from patients with ME and long COVID and healthy controls were compared using a Kruskal-Wallis *H* test. Box plots display individual data points, median (center line) and the first and third quartiles (lower and upper bound of box). Pre-bed rest and healthy controls are displayed in white, post-bed rest is displayed in grey, patients with long COVID are displayed in orange, and patients with ME/CFS are displayed in green.

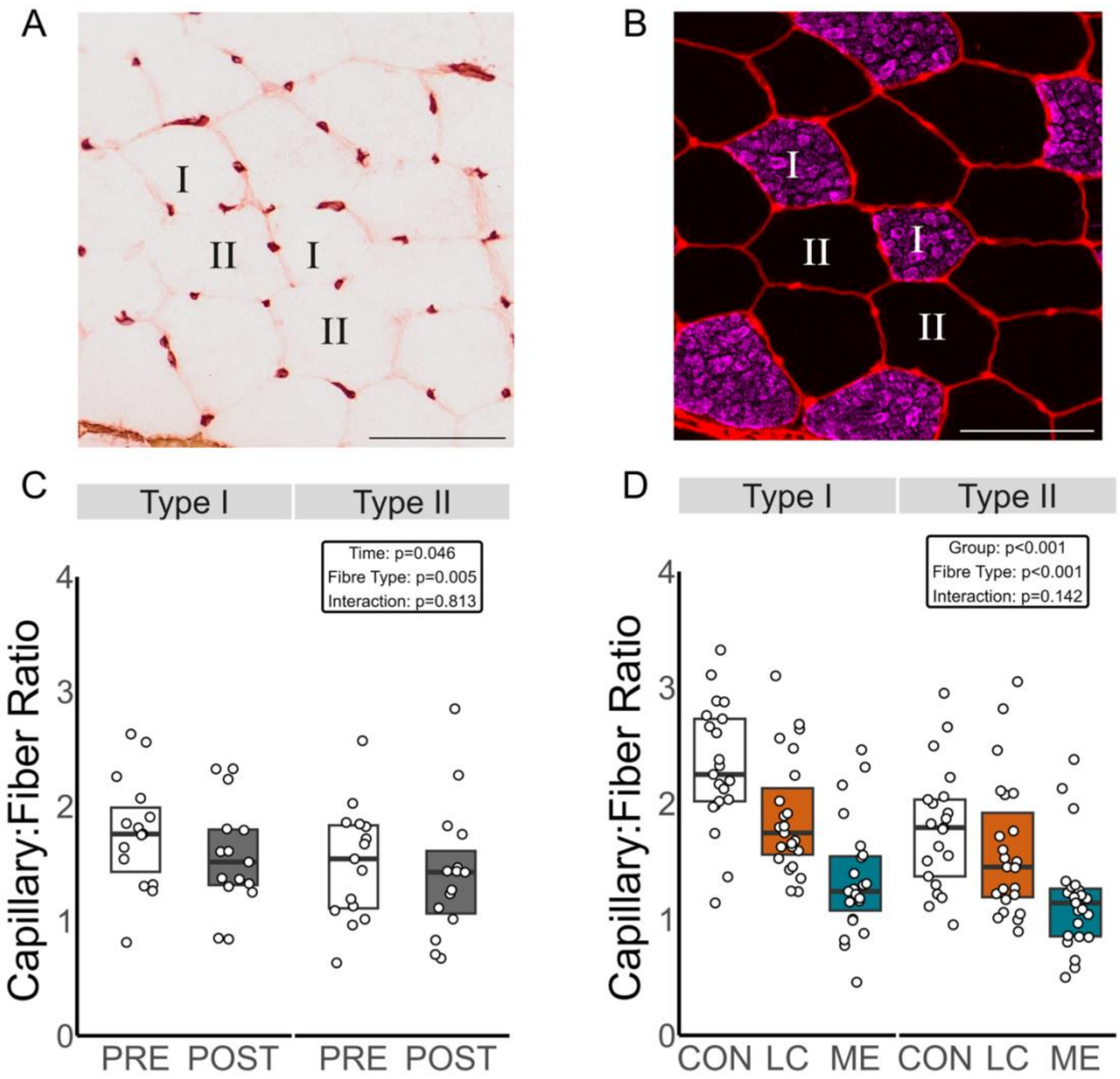

Supplemental Figure 11. **Capillary-to-fiber ratio is higher in type I fibers compared to type II fibers in all groups, while bed rest and both patient groups have lower capillary-to-fiber ratios in both fiber types.** Fiber type specific capillary-to-fiber ratios were analyzed by overlaying capillary staining (**A**) with fiber type staining (**B**) in a subgroup of participants, where possible. Fiber types were classified as type I positive (I) or type I negative (II). Bed rest participants (n=15) exhibited a reduction in capillary-to-fiber ratio for both fiber types ubiquitously (**C**). Patients with ME/CFS (ME/CFS; ME, n=23) had lower capillary-to-fiber ratios in both fiber types compared to both patients with long COVID (LC, p=0.0013, n=23) and healthy controls (CON, p<0.001, n=21; **D**). Patients with long COVID had the tendency (p=0.096) to have lower capillary-to-fiber ratios than healthy controls. In all groups, type I fibers displayed greater capillary-to-fiber ratios compared to type II fibers. After Box-Cox transformations, all data remained non-normally distributed. Data from bed rest participants were compared using a repeated measures 2-way aligned rank transform test. Data from patients with ME/CFS, patients with long COVID and healthy controls were compared using a 2-way aligned rank transform test, with a pairwise estimated marginal means post-hoc test for group effects. Box plots display individual data points, median (center line) and the first and third quartiles (lower and upper bound of box). Pre-bed rest and healthy controls are displayed in white, post-bed rest is displayed in grey, patients with long COVID are displayed in orange, and patients with ME/CFS are displayed in green. Scale bars represent 100 µm.

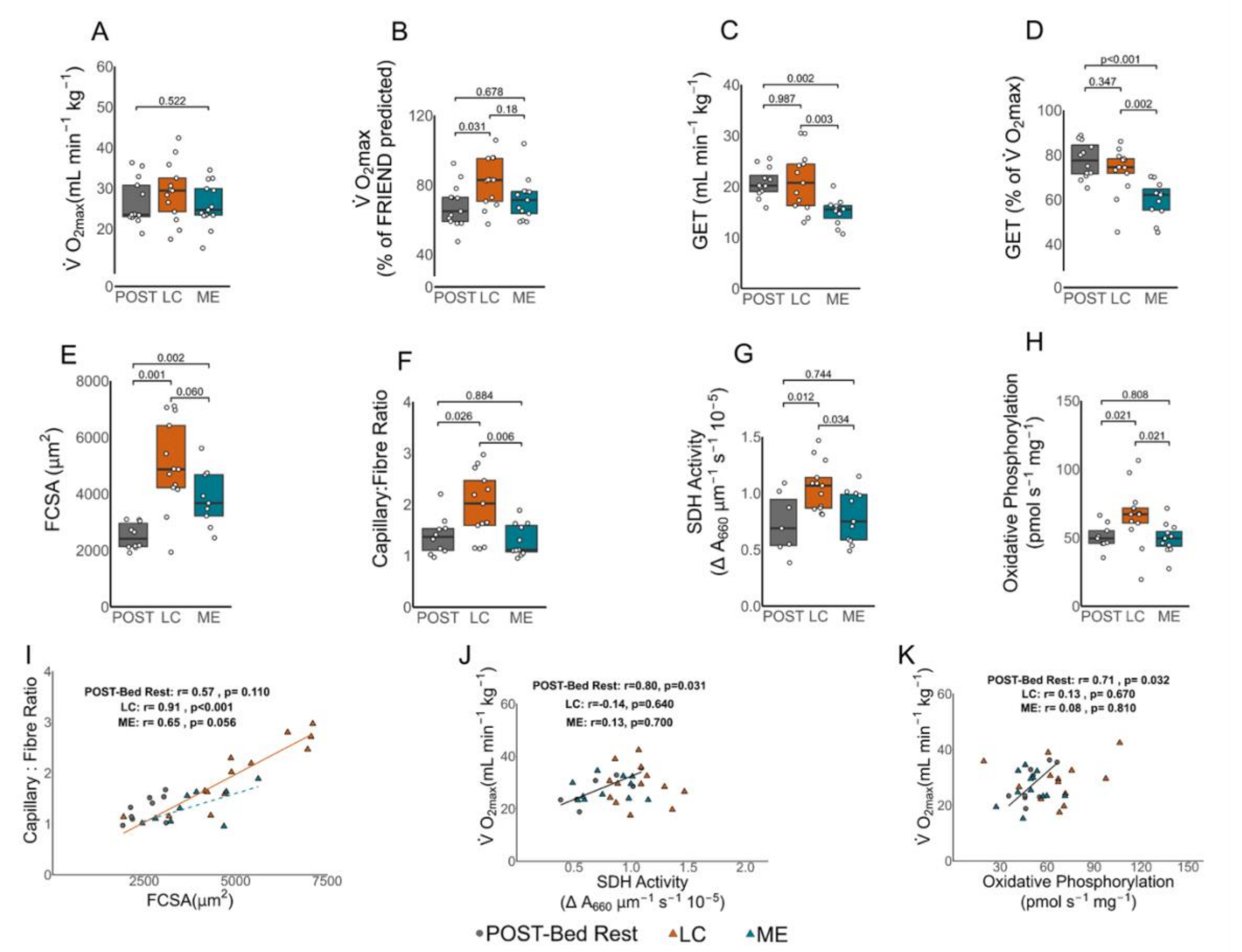

Supplemental Figure 12. **Despite similar $\dot{V}O_{2max}$ between post-bed rest and both patients with long COVID and ME/CFS, skeletal muscle alterations are different following bed rest compared to both patients with long COVID and ME/CFS.** A sub-analysis was conducted on age- and sex-matched individuals between post-bed rest (n=13), patients with long COVID (LC, n=13) and patients with myalgic encephalomyelitis/chronic fatigue syndrome (ME/CFS; ME, n=13). There were no differences in $\dot{V}O_{2max}$ between groups (**A**), however patients with long COVID tended to have higher % of predicted $\dot{V}O_{2max}$ based on the FRIEND equation (ANOVA: *p*=0.076; **B**). The gas exchange threshold was lower in patients with ME/CFS (n=11) than patients with long COVID (n=13; **C**). The gas exchange threshold relative to $\dot{V}O_{2max}$ was also earlier in patients with ME/CFS compared to post-bed rest and patients with long COVID (**D**). Skeletal muscle fiber cross-sectional area (FCSA) was lower after bed rest (n=10) compared to either ME/CFS (n=9) or long COVID (n=13), while there was a trend for patients with ME/CFS to present with smaller fibers than patients with long COVID (**E**; *p*=0.060). Capillary-to-fiber ratio was higher in long COVID (n=13) than either post-bed rest (n=10) or patients with ME/CFS (n=11; **F**). Similarly, succinate dehydrogenase (SDH) activity (**G**) and oxidative phosphorylation capacities (**H**) were higher in patients with long COVID (n=13, n=13) than either post-bed rest (n=7, n=13) or patients with ME/CFS (n=11, n=13). All groups had, or tended to have, a significant relation between FCSA and capillary-to-fiber ratio (**I**). There was a correlation between $\dot{V}O_{2max}$ and either SDH activity (**J**) or oxidative phosphorylation capacity (**K**) in participants after bed rest, but not in either patient group. Data from $\dot{V}O_{2max}$, gas exchange threshold relative to $\dot{V}O_{2max}$, FCSA, and oxidative phosphorylation remained non-normally distributed after Box-Cox transformations. Parametric comparisons were assessed using ANOVA, with Tukey HSD post-hoc testing. Non-parametric comparisons were assessed using Kruskal–Wallis *H* test, with pairwise Wilcoxon tests with Benjamini–Hochberg correction post-hoc. Box plots display individual data points, median (center line) and the first and third quartiles (lower and upper bound of box). Pre-bed rest and healthy controls are displayed in white, post-bed rest is displayed in grey, patients with long COVID are displayed in orange, and patients with ME/CFS are displayed in green. Solid lines represent significant correlations, dashed lines represent correlations with *p*<0.10.

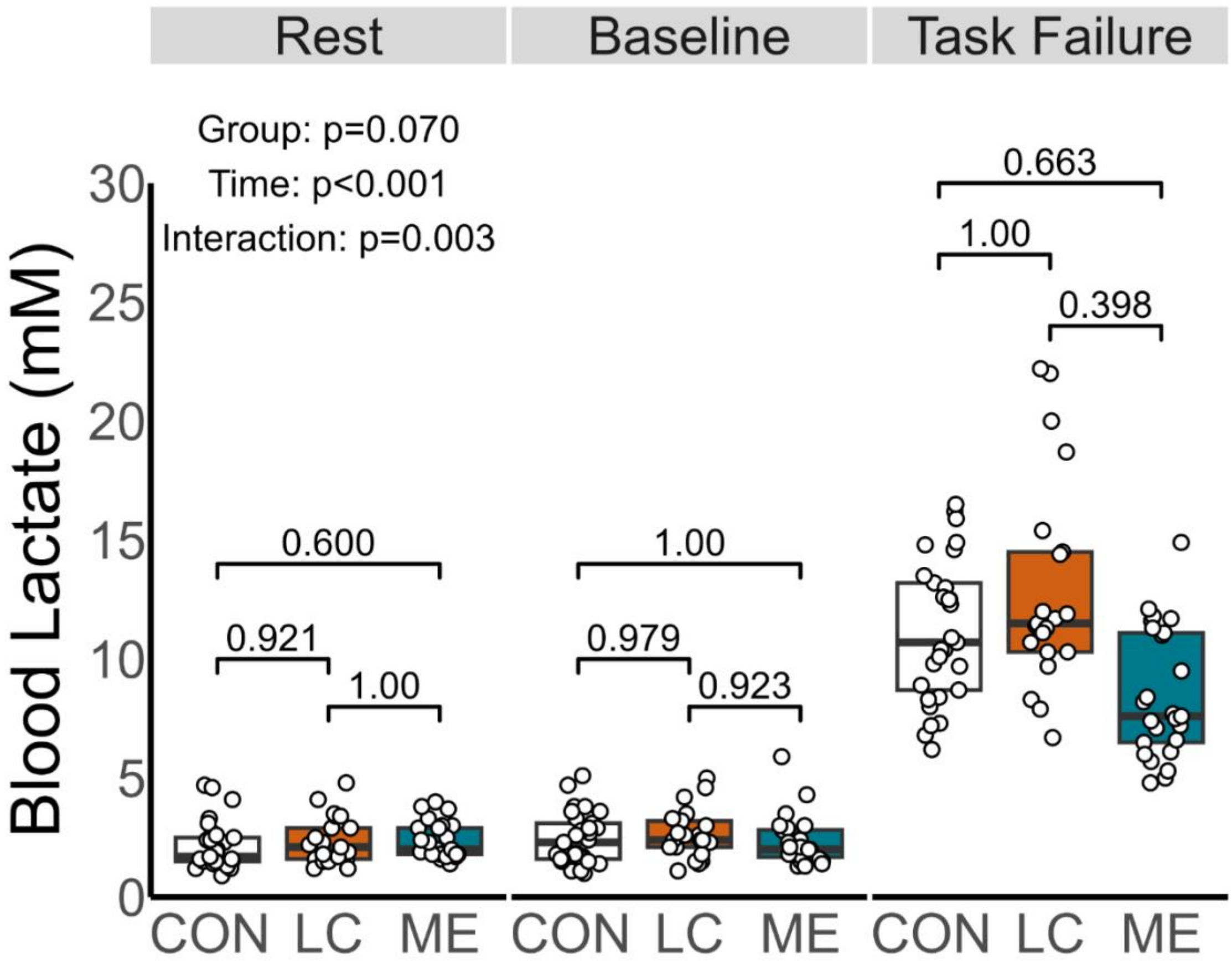

Supplemental Figure 13.**Blood lactate increased in patients with long COVID, patients with ME/CFS, and healthy controls, with patients with ME/CFS having a smaller increase in blood lactate at task failure.** Blood lactate measurements of healthy controls (CON, n=29), patients with long COVID (LC, n=21), and patients with myalgic encephalomyelitis/chronic fatigue syndrome (ME/CFS; ME, n=24) before and after maximal cardiopulmonary exercise testing. All groups showed elevations of blood lactate at task failure. No differences were found between rest and baseline in any group. Neither patient group was significantly different from healthy controls at any timepoint. After Box-Cox transformations, data at task failure was non-normally distributed. Data was compared using a repeated measures aligned rank transformation test with pairwise estimated marginal means post-hoc for interaction effects. Box plots display individual data points, median (center line) and the first and third quartiles (lower and upper bound of box). Pre-bed rest and healthy controls are displayed in white, post-bed rest is displayed in grey, patients with long COVID are displayed in orange, and patients with ME/CFS are displayed in green.
