# Supplementary material for: Skeletal muscle properties in long COVID and ME/CFS differ from those induced by bed rest
Source: Nat Commun. 2026 Jul 28;17:9125. doi: 10.1038/s41467-026-75725-y (PMC13518852; doi:10.1038/s41467-026-75725-y)
Supplement: Supplementary file 2 [file 41467_2026_75725_MOESM2_ESM.pdf]

nature portfolio

Peer Review File

Skeletal muscle properties in long COVID and ME/CFS differ from those induced by bed rest

Corresponding Author: Dr Rob Wüst

This file contains all reviewer reports in order by version, followed by all author rebuttals in order by version.

Version 1:

Reviewer comments:

Reviewer #1

(Remarks to the Author)
This paper presents an opportunistic and, perhaps, insightful cross-sectional comparison among long COVID and ME/CFS patients and also a longitudinal enforced bed rest cohort. The battery of exercise and skeletal muscle data is impressive and is analyzed to gain novel insights into the mechanisms responsible for the very low exercise capacity in the long COVID and ME/CFS patients. Given differential patterns of adaptation/degradation in skeletal muscle, specifically the absence of atrophy in COVID and ME/CFS patients, Charlton et al. conclude that "traditional rehabilitation strategies may not apply to these cohorts". Cobbling these groups together is an ambitious undertaking that involved navigating substantial previously published data. Although the authors are to be complimented on the litany of measurements and this extraordinary undertaking it presents as a plethora of results that assaults the reader without a strongly-defined overarching model. Development of such would help clarify the important responses from the chaff.
Other weaknesses that detract from otherwise high enthusiasm include: 1. Lack of longitudinal data in COVID, ME/CFS patients. 2. The bed rest condition is head-down tilt to better represent space flight, presumably. This is very different from just bed rest especially as regards fluid shifts that will impact cardiovascular function and this group are a decade younger than the patients and their control group. 3. The maximal exercise tests (ramp, step) did not include a validation step to provide confidence that VO2max was actually measured, doi: 10.1152/japplphysiol.01063.2016). It is implausible that all subjects exhibited a plateau of VO2 vs work rate – please provide data substantiating such. The statement in lines 442-3 does not cohere with laboratory findings elsewhere. 4. A better model of VO2max limitation is needed. Possibly using the eponymous "Wagner diagram." 5. In some respects it felt as though the authors were taking a stab at physiological mechanisms without developing theory based upon the latest evidence e.g., microvascular function, oxygen movement through tissues.

SPECIFIC/MINOR CONCERNS
LINE(S)
57 Reads like a non-sequitur. Might it not also just lead to lack of adaptations wherein the muscle remains the same? Please clarify.
63-67 Why if there are some "indications of atrophy" in COVID and ME/CFS is it later hypothesized that patients will display no muscle atrophy (line 72)?
75 By "tissue markers for oxygen supply" is capillary density or such meant? If so, without measurements of capillary function and RBC flux, distribution and hematocrit structure itself is a poor surrogate of "oxygen delivery."
121 Why would maximal heart rate increase? Is there precedence for this? Perhaps they just pushed themselves harder and had more experience in later tests? This is why a validation test is crucial.
154-156 Anyone who has read Peter Wagner's papers on VO2max determinants would not consider a single limiting step in the oxygen pathway (e.g., Exerc Sport Sci Rev. 2000 Jan;28(1):10-4 and doi: 10.1146/annurev.ph.58.030196.000321).
181 Perhaps some of the latest concepts in intramyocyte oxygen movement should be considered (e.g., doi: 10.1007/s00421-021-04854-7).
234 "hyperventilation" is defined as PCO2<40 mmHg. This was not measured nor were ventilatory patterns reported.
298-300 These ideas need better development.
304-306 Maybe in mice but are they in humans?
310 Be specific. What evidence for "local hypoxia" were you looking for?
303-312 Needs rewriting for clarity.
314- Please provide more evidence that fiber type transformation did or did not happen. Sometimes authors interpret smaller

fiber XSA – and thus less total area of a given fiber type – as evidence for transformation when it is not.

(Remarks on code availability)

Reviewer #2

(Remarks to the Author)
The study aimed to compare the exercise responses and skeletal muscle adaptations in long COVID and ME/CFS patients with those following 60-day strict bed rest. The hypothesis was that while aerobic capacity would be similarly reduced in patients and after bed rest, skeletal muscle adaptations would differ significantly. Patients were expected to show no muscle atrophy but reduced capillary-to-fiber ratios and densities, whereas bed rest was anticipated to lead to severe muscle atrophy and altered capillary ratios.
Key hypotheses and focuses:
i) Comparison of CPET responses and muscle adaptations in patients with long COVID and ME/CFS and Controls with those under strict bed rest.
ii) Expectation of differing skeletal muscle markers between patients and bed rest participants.
iii) Examining associations between muscle markers and exercise capacity across these groups through aerobic exercise responses and muscle biopsies.

What are the noteworthy results?
The remarkable results relate to the fact that a 60-day bed rest period was used as a time window for deconditioning effects. This is an exceptional approach. Furthermore, the muscle physiological/pathophysiological response to exercise was compared between long COVID, ME/CFS, healthy controls, and bed rest controls with remarkable experimental quality.

Will the work be of significance to the field and related fields?
The study will be of importance to the field and related disciplines in the area of post-acute infectious syndromes. According to current knowledge, no comparable protocols exist in the literature for the combination of bed rest and post-infectious diseases.

Does the study support the conclusions and claims, or is additional evidence needed?
The study's results indicate that deconditioning is not the sole factor explaining post-exercise-induced symptom exacerbation. On the other hand, the study cannot separate the contribution of deconditioning from other proven physiological factors.

Are there any deficiencies in the data analysis, interpretation, and conclusions? Do these preclude publication or require revision?
The following revisions (adjustments) are required:
i) Clinical case definition: The clinical case definition for ME/CFS needs to be clarified, as the ME/CFS diagnosis was made before 2020. Report the following aspects: type of infection (respiratory tract, EBV, gastroeneritis, inflamed bowel disease, rheumatological aspects, autonomic symptoms, e.g., POTS, dyspnea).
ii) For both groups (long COVID and ME/CFS): Report the frequency and severity of symptoms and the DSQ items in a table. This presents an opportunity for the paper, as Q9, for example, allows for differentiation between < 14 hours and > 14 hours (e.g., Kedor et al. 2022, https://doi.org/10.1038/s41467-022-32507-6). The data analysis should be expanded to include, for example, correlative measures (e.g., VO2-heart rate slope).
iii) Please report the lactate values at all sampling times (e.g., in Supplementary Figure 2).
iV) The muscle-associated analyses are excellent and show that deconditioning alone cannot explain PEM. This is very valuable and helpful. It is very helpful if you can summarize the pathophysiological changes (respiratory, cardiovascular, muscle physiological) in the discussion with regard to the following aspects: finding, pathophysiological interpretation concerning deconditioning and other explanations (e.g. Haunhorst et al. 2025, PMCID: PMC11825644).

(Remarks on code availability)
The source code is available as an R script as supplementary material to the document. Please add a README file with sufficient instructions for installation and execution. The code and all necessary notes are available at https://github.com/bcn95/LC_ME_CFS_BED_REST. The code can be installed and executed.

Reviewer #3

(Remarks to the Author)
Determining how much of the exertional intolerance characteristic of ME/CFS and Long COVID is related to simple deconditioning vs. disease is critical. The study aims to determine if decreased exercise capacity in both diseases can be linked to intrinsic skeletal muscle abnormalities that differ from those found in de-training. This represents an important step in the field as post exertional malaise precludes traditional exercise rehabilitation approaches that have been found to be useful in deconditioning.

The authors' key findings suggest bed rest caused muscle atrophy and reduced oxidative phosphorylation, while patients did not show muscle atrophy but had fewer capillaries and more glycolytic fibers.

The presentation of the data could be improved with some minor modifications:

-bedrest subjects was 66% male whereas healthy controls and patients was 50% male. The absolute VO2 max for males will be higher than females. VO2 max comparisons among groups should be expressed as percent of predicted, whose independent variables are driven by age, sex, and height. Ideal matching of VO2 max would be as a percent of predicted not absolute.

-the asymmetric sex distribution across groups and its influence on whole body exercise and muscle metabolism should be discussed and alluded to in the limitations section.

-there is a wide range of daily steps and absolute VO2 max in patients; it would be important to know how much of that variance is related to sex, training status and the disease itself.

-the influence of the head down position during bed rest should be discussed as a potential confounder (any deconditioning in patients would not be related to that body position). Dysautonomia is part of ME and LC exercise intolerance and arterial baroreceptors are reset according to body position.

-lines 236 and 237: increased physiologic dead space ventilation does not lead to hyperventilation, rather it leads to hyperpnea; this should be so indicated. Respiratory muscle weakness does not lead to hyperventilation or hyperpnea.

-264-274: the relationships between muscle mitochondrial density/ function vs. VO2 max in patients would be strengthened by expressing VO2max as a percent predicted. This would decrease the confounding effects of sex and body size.

-461: your group has nicely shown alterations in skeletal muscle mitochondrial function as a result of prior exercise and PEM in the patient group. Please indicate when your muscle biopsies were obtained related to the CPET and what was done to assure that patients were biopsied while in a "steady state" not during an episode of PEM.

-has your group considered triple staining of capillaries and muscle fiber types; see, eg., Bailly M, et al. Two new reliable immunohistochemical methods for simultaneous identification of capillaries, the three types of fibers and basal lamina in human skeletal muscle. Histochem Cell Biol. 2020 Sep;154(3):327-337. doi: 10.1007/s00418-020-01895-5. Epub 2020 Jun 26. PMID: 32591977. It would be extremely interesting to know if capillarization of type 1 versus type 2 fibers were differentially affected by bedrest and the two diseases.

(Remarks on code availability)

Reviewer #4

(Remarks to the Author)
I co-reviewed this manuscript with one of the reviewers who provided the listed reports. This is part of the Nature Communications initiative to facilitate training in peer review and to provide appropriate recognition for Early Career Researchers who co-review manuscripts.

(Remarks on code availability)

Version 2:

Reviewer comments:

Reviewer #1

(Remarks to the Author)
This paper is much improved in revision. The following points remain to be resolved, however.
Line(s)
142 How, specifically, might altered ventilatory patterns lower VO2max? There was no evidence for arterial O2 desaturation during maximal exercise was there?
143 Isn't an "elevation in VO2-HR slope" indicative of greater O2 extraction (or SV)? Please rewrite for clarity.
299 As before, please explain how maximal HR increases with bed rest.
301- As above, increased VO2-HR slope would indicate increased SV or peripheral O2 extractions???
341 Less obliquely cut capillaries surely?
354 No! Convective O2 delivery i.e., blood flow is controlled at the arteriolar not capillary level in skeletal muscle.
372 The Broxterman et al. paper cited is small muscle mass exercise where the CV limitation/ceiling is removed….a crucial distinction.

(Remarks on code availability)
I received the following: Your search did not match any documents

Reviewer #2

(Remarks to the Author)
***
Required revision i):
The following revisions (adjustments) are required:
i) Clinical case definition: The clinical case definition for ME/CFS needs to be clarified, as the ME/CFS diagnosis was made before 2020. Report the following aspects: type of infection (respiratory tract, EBV, gastroeneritis, inflamed bowel disease, rheumatological aspects, autonomic symptoms, e.g., POTS, dyspnea).

Response from the Reviewer: The requested revisions have been implemented and substantively strengthen the manuscript. Supplemental Figure 1 now presents the onset data for the cohort in detail. As acknowledged in the ME/CFS literature, recall bias regarding precise onset timing is a well-recognized methodological challenge; accordingly, a majority of participants reported an unknown exact onset while nonetheless identifying a distinct point in time at which symptoms emerged. Importantly, this pattern is entirely consistent with — and indeed expected under — the Canadian Consensus Criteria (CCC), thereby confirming that the case definition adheres to the most stringent and widely accepted diagnostic framework in the field. The presented data also demonstrate that this is not an exclusively long COVID cohort.

***
Required revision ii):
ii) For both groups (long COVID and ME/CFS): Report the frequency and severity of symptoms and the DSQ items in a table. This presents an opportunity for the paper, as Q9, for example, allows for differentiation between < 14 hours and > 14 hours (e.g., Kedor et al. 2022, https://doi.org/10.1038/s41467-022-32507-6). The data analysis should be expanded to include, for example, correlative measures (e.g.,
VO2-heart rate slope).
Response from the Reviewer: The DSQ-PEM data presented in the supplemental figures provide compelling and transparent evidence for the diagnostic validity of the Long COVID and ME/CFS cohort. The finding that all patients with Long COVID and ME/CFS met the threshold for a positive PEM diagnosis via the DSQ-PEM — while no healthy controls did — unambiguously confirms that the cohort fulfills the Canadian Consensus Criteria (CCC) for ME/CFS, with PEM scores consistently exceeding the required threshold of > 14. Moreover, the significantly elevated severity and frequency scores, the more negative responses to DSQ-PEM items 6–8 and 10, as well as the markedly prolonged recovery durations following activity, collectively indicate that the cohort represents severely affected individuals. This characterization of disease severity to be of considerable importance for the interpretation of the findings, as it establishes a well-defined and clinically meaningful patient population and thereby strengthens the generalizability and clinical relevance of the reported results.

***
Required revision iii):
iii) Please report the lactate values at all sampling times (e.g., in Supplementary Figure 2).
Response from the Reviewer: For the results of the paper, it is very significant that no significant differences exist for the investigated conditions concerning lactate. This may also be an argument against an effect of decoding alone... .
***

***
Required revision iv):
iV) The muscle-associated analyses are excellent and show that deconditioning alone cannot explain PEM. This is very valuable and helpful. It is very helpful if you can summarize the pathophysiological changes (respiratory, cardiovascular, muscle physiological) in the discussion with regard to the following aspects: finding, pathophysiological interpretation concerning deconditioning and other explanations (e.g. Haunhorst et al. 2025, PMCID: PMC11825644).
Response from the Reviewer: Thank you for the evidence-based classification that skeletal muscle mitochondrial dysregulation is associated with PEM (the data of the presented paper). This possibly points to a phenotypic approach.
***

(Remarks on code availability)

Thanks for the README file in the Source Code, there are enough instructions for installing and running!
The R-Code is transparent documented.

Reviewer #3

(Remarks to the Author)
Thank you for the thoughtful responses to my review. All of my questions have been adequately addressed.

(Remarks on code availability)

Reviewer #4

(Remarks to the Author)

I co-reviewed this manuscript with one of the reviewers who provided the listed reports. This is part of the Nature Communications initiative to facilitate training in peer review and to provide appropriate recognition for Early Career Researchers who co-review manuscripts.

(Remarks on code availability)

Version 3:

Reviewer comments:

Reviewer #1

(Remarks to the Author)
The authors have addressed all concerns

(Remarks on code availability)

Reviewer #2

(Remarks to the Author)
All reviewer comments have been addressed, and the manuscript is significantly improved compared to the initial version. I have no further comments.

(Remarks on code availability)
no further comments to the code: the code is fine, complete open source (github) and is very well documented

Open Access This Peer Review File is licensed under a Creative Commons Attribution 4.0 International License, which permits use, sharing, adaptation, distribution and reproduction in any medium or format, as long as you give appropriate credit to the original author(s) and the source, provide a link to the Creative Commons license, and indicate if changes were made.
In cases where reviewers are anonymous, credit should be given to 'Anonymous Referee' and the source.
The images or other third party material in this Peer Review File are included in the article's Creative Commons license, unless indicated otherwise in a credit line to the material. If material is not included in the article's Creative Commons license and your intended use is not permitted by statutory regulation or exceeds the permitted use, you will need to obtain permission directly from the copyright holder.
To view a copy of this license, visit https://creativecommons.org/licenses/by/4.0/

Reviewer #1 (Remarks to the Author)

This paper presents an opportunistic and, perhaps, insightful cross-sectional comparison among long COVID and ME/CFS patients and also a longitudinal enforced bed rest cohort. The battery of exercise and skeletal muscle data is impressive and is analyzed to gain novel insights into the mechanisms responsible for the very low exercise capacity in the long COVID and ME/CFS patients. Given differential patterns of adaptation/degradation in skeletal muscle, specifically the absence of atrophy in COVID and ME/CFS patients, Charlton et al. conclude that "traditional rehabilitation strategies may not apply to these cohorts". Cobbling these groups together is an ambitious undertaking that involved navigating substantial previously published data. Although the authors are to be complimented on the litany of measurements and this extraordinary undertaking it presents as a plethora of results that assaults the reader without a strongly-defined overarching model. Development of such would help clarify the important responses from the chaff.

> Response: We thank the reviewer for the comments. Below, we have outlined where we have improved the manuscript based on the reviewer comments.

Other weaknesses that detract from otherwise high enthusiasm include:

1. Lack of longitudinal data in COVID, ME/CFS patients.

> Response: We agree that longitudinal data in patients with long COVID and ME/CFS would provide more insight into disease progression and causal mechanisms. However, repeated skeletal muscle biopsies in these patient groups are not currently available, and are ethically demanding. Importantly, even a longitudinal design would not fully resolve the relative contributions of physical inactivity versus disease-intrinsic mechanisms (e.g., immune dysregulation, viral persistence, or spontaneous recovery), as these factors evolve concurrently and cannot be ethically or practically manipulated in patients. We have therefore explicitly acknowledged the lack of longitudinal patient data as a limitation and have framed our conclusions to focus on distinguishing inactivity-induced adaptations from disease-associated skeletal muscle characteristics, rather than on disease progression *per se*.
>
> Lines 421-427:
>
> *"A limitation of the present study is its cross-sectional design in patients with long COVID and ME/CFS, which precludes inference on temporal disease progression or causality. While longitudinal muscle biopsies would be informative, such data are currently unavailable, and ethical constraints limit experimental manipulation of activity levels in patients. Consequently, our conclusions are restricted to demonstrating that the skeletal muscle*

phenotype observed in patients differs from that induced by prolonged bed rest, rather than establishing the temporal origin of these abnormalities."

2. The bed rest condition is head-down tilt to better represent space flight, presumably. This is very different from just bed rest especially as regards fluid shifts that will impact cardiovascular function and this group are a decade younger than the patients and their control group.

Response: We thank the reviewer for the critique. Indeed, the head down tilt was used as a space flight memetic, as the study was conducted in collaboration with NASA and European Space Agency, and head-down tilt bed rest induces a slightly greater cephalic fluid shift compared to horizontal bed rest. We agree that head-down tilt bed rest is not completely equivalent to ambulatory physical inactivity and introduces haemodynamic features that slightly differ from those experienced by patients with long COVID or ME/CFS, particularly with respect to sustained fluid redistribution and loss of orthostatic loading. Plasma volume significantly decreases after long-term bed rest, and these effects may differentially influence cardiovascular responses compared to patients, in whom preload failure occurs due to dysautonomia.

Our intention in using the head-down tilt model was not to claim physiological equivalence to patient deconditioning, but to employ one of the few available long-term, tightly controlled models of severe physical inactivity in humans. Thus, we feel that the results of this head-down tilt bed rest can be compared with patients with long COVID and ME/CFS despite the limitations induced by the fluid shift *per se*. The cost of performing such studies are around 1 million US dollars per participant (we included 24 participants in this analysis). As such, we feel that such a relatively simple post-hoc cross-comparison is valuable for this purpose. However, we have revised the Discussion and Limitations to explicitly acknowledge that cardiovascular comparisons should be interpreted with caution, and that the bed-rest comparison is most informative for skeletal muscle structural and metabolic adaptations rather than haemodynamic mechanisms *per se*.

We have now noted that the fluid redistribution, loss of orthostatic loading, and plasma volume loss may have implications on the vasculature in the Discussion (lines 336-339), in which the lack of shear stress may contribute to a reduction in capillary area, which may also implicate changes to capillarization itself.

Lines 340-342:

"This may be […] due to the lack of shear stress experienced during head-down tilt resulting in a structural remodeling of the capillary beds[54]"

We also noted the age difference. Due to the large age difference between cohorts, we already added the supplementary analysis of age- and sex-matching between the pre-bed rest condition and healthy controls, for comparison of how similar the control groups are (see Supplemental Figure 4). We now also included the age- and sex-matched bed rest cohort with the long COVID and ME/CFS patients as an exploratory analysis (see Supplemental Figure 12, provided below). While this is clearly an underpowered analysis, as neither study design was meant to accommodate this analysis, we believe that in doing so, it further supports the data when analyzed separately, as they largely yield similar results.

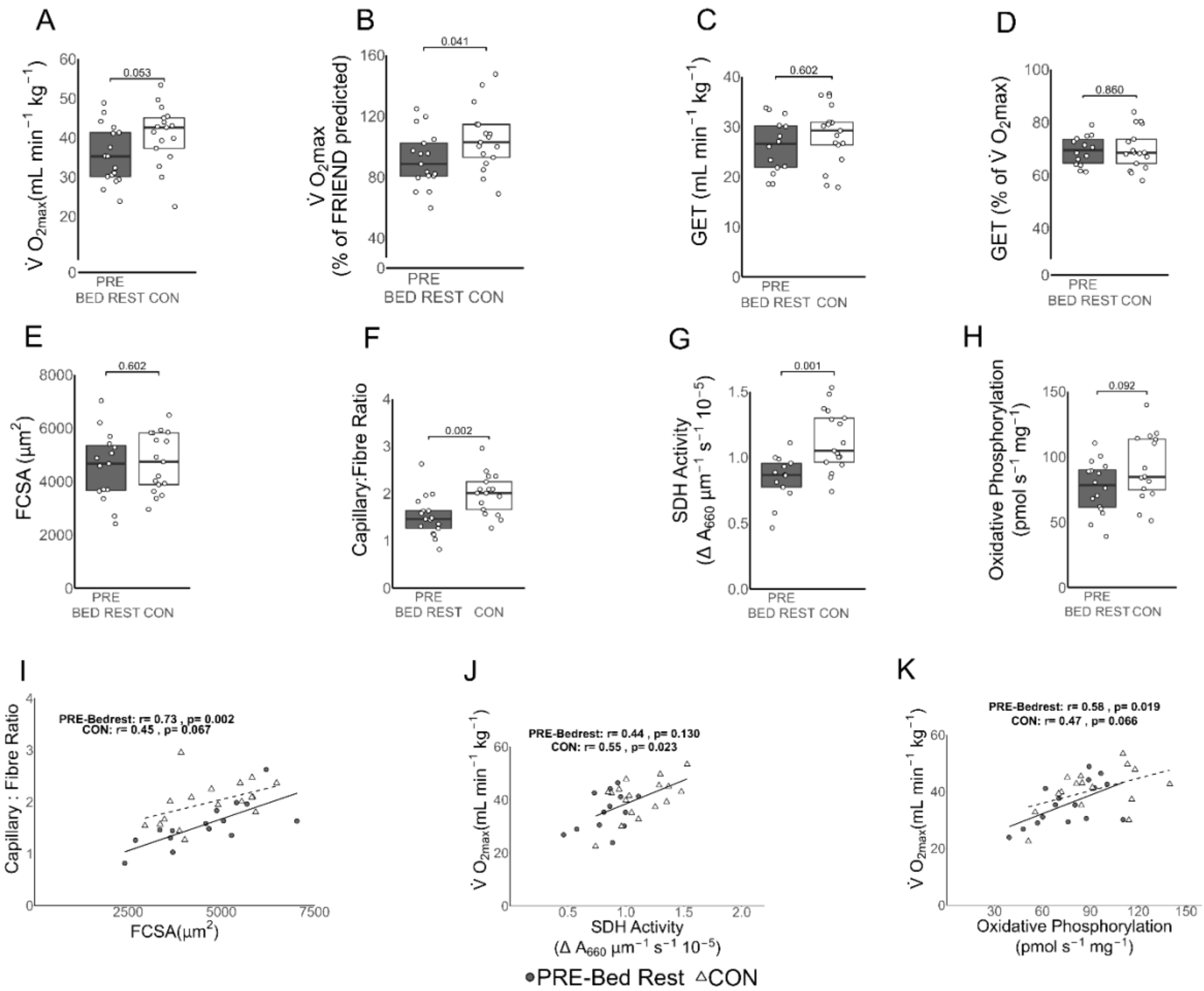

***Supplemental Figure 4**: A sub-analysis was conducted on age- and sex-matched individuals between healthy individuals pre-bed rest (n=17) and healthy individuals matched to patients with long COVID and ME/CFS (n=17; Supplemental Table 1). There was a tendency for $\dot{V}O_{2max}$ to be lower in bed rest participants compared to the patient-matched healthy controls (**A,** P=0.053) when expressed relative to body weight, and a significant difference when expressed as % of predicted $\dot{V}O_{2max}$ based on the FRIEND equation (**B**). However, there were no differences in in gas exchange threshold (GET) (**C**), the occurrence of the gas exchange threshold relative to $\dot{V}O_{2max}$ (**D**) or*

*fiber cross sectional area (FCSA, **E**) between groups. Participants before bed rest had a lower capillary-to-fiber ratio (**F**), lower succinate dehydrogenase (SDH) activity (**G**), and oxidative phosphorylation capacity (**H**) than patient-matched healthy controls. Both groups had, or tended to have a significant relation between FCSA and capillary-to-fiber ratio (**I**). Only patient-matched healthy controls had a significant relation between $\dot{V}O_{2max}$ and SDH activity (**J**). Both groups had, or tended to have, a significant association between $\dot{V}O_{2max}$ and oxidative phosphorylation capacity (**K**). Data for $\dot{V}O_{2max}$, FCSA, and SDH activity remained non-normally distributed after Box-Cox transformations. Parametric comparisons were assessed using non-paired t-tests. Non-parametric comparisons were assessed using Wilcoxon signed rank tests. Solid lines represent significant correlations, dashed lines represent correlations with P<0.10.*

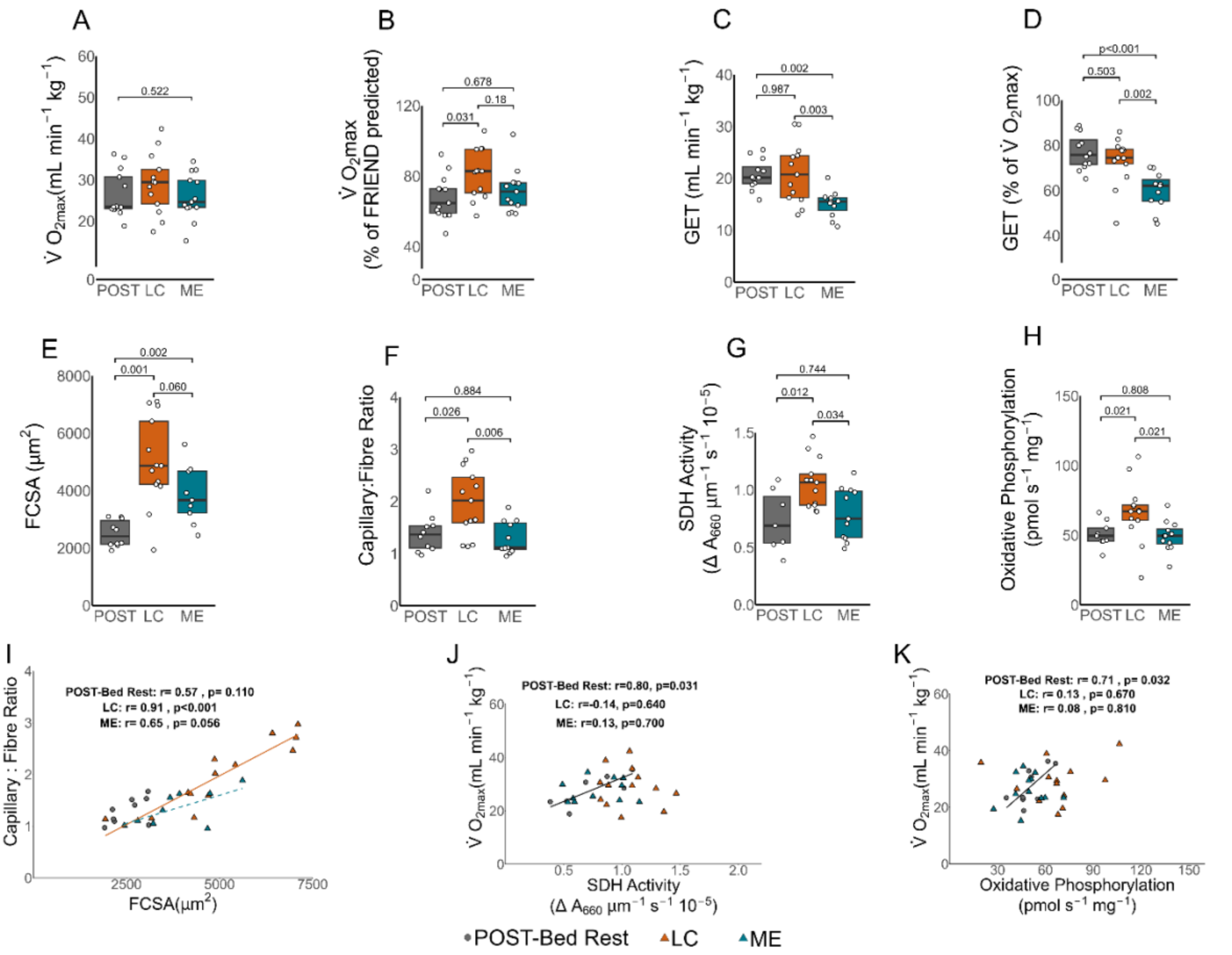

***Supplemental Figure 12**: A sub-analysis was conducted on age- and sex-matched individuals between post-bed rest (n=13), patients with long COVID (LC, n=13) and patients with ME/CFS (n=13). There were no differences in $\dot{V}O_{2max}$ between groups (**A**), however patients with long COVID had significantly higher % of predicted $\dot{V}O_{2max}$ based on the FRIEND equation (**B**). The gas exchange threshold was lower in patients with ME/CFS than patients with long COVID (**C**). The gas exchange threshold relative to $\dot{V}O_{2max}$ was also*

*earlier in patients with ME/CFS compared to post-bed rest and patients with long COVID (**D**). Skeletal muscle fiber cross-sectional area (FCSA) was lower after bed rest compared to either ME/CFS or long COVID, while there was a trend for patients with ME/CFS to present with smaller fibers than patients with long COVID (**E**). Capillary-to-fiber ratio was higher in long COVID than either post-bed rest or patients with ME/CFS (**F**). Similarly, succinate dehydrogenase (SDH) activity (**G**) and oxidative phosphorylation capacities (**H**) were higher in patients with long COVID than either post-bed rest or patients with ME/CFS. All groups had, or tended to have, a significant relation between FCSA and capillary-to-fiber ratio (**I**). There was a correlation between* $\dot{V}O_{2max}$ *and either SDH activity (**J**) or oxidative phosphorylation capacity (**K**) in participants after bed rest, but not in either patient group. Data from* $\dot{V}O_{2max}$*, gas exchange threshold relative to* $\dot{V}O_{2max}$*, FCSA, and oxidative phosphorylation remained non-normally distributed after Box-Cox transformations. Parametric comparisons were assessed using ANOVA, with Tukey HSD post-hoc testing. Non-parametric comparisons were assessed using Kruskal–Wallis H test, with pairwise Wilcoxon tests with Benjamini–Hochberg correction post-hoc. Solid lines represent significant correlations, dashed lines represent correlations with P<0.10.*

3. The maximal exercise tests (ramp, step) did not include a validation step to provide confidence that VO2max was actually measured, doi: 10.1152/japplphysiol.01063.2016). It is implausible that all subjects exhibited a plateau of VO2 vs work rate – please provide data substantiating such. The statement in lines 442-3 does not cohere with laboratory findings elsewhere.

Response: Thank you, we agree that this was a premature statement. We have reevaluated the plateaus in line with Poole et al 2008 and Midgley 2009, and have provided secondary end point justifications in a supplementary table, now integrated into Supplemental Table 2 (included below).

Supplemental Table 2: Primary and secondary endpoint criteria of $\dot{V}O_{2max}$ testing. Values represent the number of participants meeting each individual primary or secondary $\dot{V}O_{2max}$ criterion. The final row indicates the number of participants who failed to meet any primary or secondary criterion. Using this criteria, only 7 of 129 tests failed to meet any accepted primary or secondary $\dot{V}O_{2max}$ criteria, indicating the vast majority of tests provided a physiologically valid estimate of maximal oxygen uptake, even in the absence of a plateau. Lactate values were not collected during the bed rest study.

**Primary Endpoints**	**Before Bedrest (n=24)**	**After Bedrest (n=24)**	**CON (n=30)**	**LC (n=25)**	**ME (n=26)**
Final 2min $\dot{V}O_2$ slope <100mL/min (n)	4	3	5	10	10
Final 2min $\dot{V}O_2$Slope >50% difference (n)	1	9	7	13	7
Points outside 95% CI in final 2min (n)	11	7	17	11	7
**Secondary Endpoints**					
Achieved Adjusted Max Heart Rate (n)	2	9	13	8	4
Achieved blood lactate >8mM (n)	ND	ND	24	19	11
Achieved RER >1.15 (n)	19	18	20	16	8
Number of participants that failed to meet any endpoint criteria (n)	3	1	0	0	3

4. A better model of VO2max limitation is needed. Possibly using the eponymous "Wagner diagram."

We agree with the reviewer that $\dot{V}O_{2max}$ should be interpreted within an integrative, multi-component framework such as the Wagner oxygen cascade, rather than implying a single dominant site of limitation. Our original wording was overly definitive in this respect. While we do not have the full set of measurements required to parameterize the Wagner diagram quantitatively, we have revised the manuscript to explicitly frame our findings within this

conceptual model. Specifically, we now clarify that although mitochondrial impairments are present, the lack of association between mitochondrial markers and $\dot{V}O_{2max}$ in patients, alongside altered capillarization and cardiovascular indices, suggests that $\dot{V}O_{2max}$ limitation in long COVID and ME/CFS likely reflects interacting upstream (i.e. convective and diffusive O_2 transport) and downstream (i.e., mitochondrial) constraints rather than a sole mitochondrial bottleneck. We have additionally included a completely new section in the discussion aimed particularly to address the $\dot{V}O_{2max}$ limitations in health and diseased conditions.

Lines 169-173:

"*Given that physical activity has been shown to shift limitations of maximal aerobic capacity from oxygen utilization to oxygen supply*[26] *and given the association between mitochondrial function and whole-body aerobic exercise capacity*[27]*, we assessed mitochondrial respiration of permeabilized muscle fibers and succinate dehydrogenase (SDH) activity in sections, to provide further insight into mitochondrial alterations upon bed rest and in patients.*"

Lines 177-184:

"*As mitochondria are the ultimate recipients in the oxygen cascade, we hypothesized that either SDH activity or oxidative phosphorylation would correlate with $\dot{V}O_{2max}$. Indeed, both SDH activity and oxidative phosphorylation capacity were correlated with $\dot{V}O_{2max}$ in healthy controls (Figure 3C+D), and such a relation was present following bed rest. However, this association was absent in patients with long COVID and ME/CFS. While the precise limitation of $\dot{V}O_{2max}$ in patients is obscured, the lack of association of $\dot{V}O_{2max}$ with mitochondrial variables suggests that reductions in exercise capacity are not limited by mitochondrial density or respiratory capacity per se.*"

Lines 358-386:

"***Limitations to $\dot{V}O_{2max}$***

$\dot{V}O_{2max}$ is determined by the integrated function of convective oxygen delivery, diffusive oxygen transport, and mitochondrial oxygen utilization, as conceptualized in the Wagner diagram[62,63]*. Although we measured several components of this pathway, we did not assess all sites required to localize the dominant limitation. Importantly, while mitochondrial oxidative capacity was reduced in patients, its lack of association with $\dot{V}O_{2max}$ (in contrast to healthy controls and bed-rest participants) argues against a purely mitochondrial limitation. Instead, our findings are consistent with the presence of interacting limitations upstream of the mitochondria, including impaired muscle oxygen diffusive capacity or maximal convective oxygen delivery,*

which cannot be resolved within the current dataset. Notably, reduced whole-body oxygen extraction does not necessarily imply low intramyocyte PO_2, as a reduction in mitochondrial oxygen utilization would be expected to reduce the diffusion gradient required to support $\dot{V}O_2$ rather than increase it. Localized reductions in tissue PO_2 would therefore require sufficiently impaired diffusive conductance or microvascular perfusion heterogeneity, mechanisms that were not directly assessed here. This situation contrasts with healthy sedentary individuals, where the primary limitation to $\dot{V}O_{2max}$ is mitochondrial[26].”

5. In some respects it felt as though the authors were taking a stab at physiological mechanisms without developing theory based upon the latest evidence e.g., microvascular function, oxygen movement through tissues.

Response: We thank the reviewer for the critique. We have revised several sections to highlight the physiological responses we expected, and what the results indicate in terms of pathophysiology. Particularly, we have removed conflations of structural indices of oxygen diffusive capacity with that of functional oxygen delivery and extraction. We have provided some examples below where we have corrected or clarified this. We have additionally added a section within the discussion regarding the interconnectivity between various parameters and their potential influence on $\dot{V}O_{2max}$ limitation.

Lines 353-356:

“The reduced capillary-to-fiber for a given muscle size may reduce the potential for convective blood flow, which may limit oxygen supply at high oxygen utilization rates (i.e. maximal exercise) or impair waste product removal in patients with long COVID and ME/CFS.”

Lines 373-386:

“Invasive CPET results indicate that at maximal exercise, arterial PO_2 in long COVID and ME/CFS is similar to healthy people[38,39]. Intramyocyte oxygen movement is dependent on convective oxygen delivery, oxygen diffusion through the tissue, and mitochondrial oxygen consumption[59]. The decrease in capillary-to-fibre ratio for a given fiber size reduces the potential for oxygen convective delivery. While the reduced mitochondrial oxidative capacity was reduced in both patients with long COVID and ME/CFS, which would suggest an elevation in intracellular PO_2, we were not able to measure intracellular PO_2 directly. However, as myoglobin is an integral piece in intramyocyte oxygen diffusion, we suspected that if intracellular PO_2 was indeed higher and oxygen convective delivery was impaired, there may be a compensatory response of myoglobin in order to maintain the oxygen diffusion gradient. As myoglobin content was similar amongst the groups, it would suggest that intramyocyte diffusion capacity may be maintained. However, it is unknown whether any of hemoglobin, capillary or myocyte membrane function is

altered, which also impacts oxygen diffusive capacity. Future work should aim to address whether membrane functions in these domains are altered and may contribute to impaired oxygen extraction."

And the new section about ***Limitations to $\dot{V}O_{2max}$'*** *on* Lines 358-386, see previous comment for details.

SPECIFIC/MINOR CONCERNS
LINE(S)
57 Reads like a non-sequitur. Might it not also just lead to lack of adaptations wherein the muscle remains the same? Please clarify.

Response: We have adjusted this to better reflect that patients may not achieve intensities or durations that would maintain adaptations, and over time may result in deconditioning. To provide a bit more background information, the ACSM recommends exercise intensities above the gas exchange threshold to induce whole-body and skeletal muscle adaptations. This is difficult for patients whose symptoms worsen at intense exercise. Unpublished data suggests that exercise intensities around the GET provides a risk factor for PEM induction. We have adjusted the sentence to:

Lines 55-57:

"*Due to PEM, long COVID and ME/CFS patients may not achieve such intensities or durations frequently enough to induce or maintain whole-body or skeletal muscle adaptations to exercise[13,14]. Depending on habitual activity levels, this may result in either maintenance of baseline muscle properties or, if activity is substantially reduced, progressive deconditioning over time[15].*"

63-67 Why if there are some "indications of atrophy" in COVID and ME/CFS is it later hypothesized that patients will display no muscle atrophy (line 72)?

Response: Thank you, the statement about 'atrophy' was confusing indeed. We unfortunately used two definitions of atrophy interchangeably, namely atrophic fibers (which are extremely small and have a very specific triangular shape), and smaller fibers in general. We have now clarified the presence of atrophic fibers compared to muscle atrophy.

Lines 65-69:

"Patients with long COVID and ME/CFS exhibit lower aerobic exercise capacity and mitochondrial respiration [6,7,9,10], reduced capillary densities[9] , muscle fiber atrophy[7], and higher prevalences of atrophic fibers (small, triangular fibers)[6], which has often been attributed to deconditioning[15,25]."

75 By “tissue markers for oxygen supply” is capillary density or such meant? If so, without measurements of capillary function and RBC flux, distribution and hematocrit structure itself is a poor surrogate of “oxygen delivery.”

> Response: Yes, we agree. This sentence has been adjusted to reflect capillarization rather than oxygen supply:
>
> Lines 69-79:
>
> *“We hypothesized that while whole body aerobic capacity would be similarly lower between patients and following bed rest, adaptations in skeletal muscle markers for oxygen supply and utilization would diverge. […], we reasoned that in a cohort of non-hospitalized patients, patients would display no muscle atrophy, whilst exhibiting reduced capillary-to-fiber ratios and capillary densities.”*

121 Why would maximal heart rate increase? Is there precedence for this? Perhaps they just pushed themselves harder and had more experience in later tests? This is why a validation test is crucial.

> Response: This is a typical phenomenon in bed rest and deconditioning studies, as the fluid shift results in marked reductions in plasma volume, which results in reductions in stroke volume. Typically, end-diastolic volume is also reduced upon bed rest. As a compensation, heart rates at rest and any given workload are elevated. This is a common finding in bed rest studies. We have now included this in the discussion on lines 296-298.
>
> Lines 298-300: “*The increase in both resting heart rate and maximal heart rate are likely reflective of a decreased stroke volume[33], potentially due to the reduced plasma volume observed after bed rest[36]. ”*

154-156 Anyone who has read Peter Wagner’s papers on VO2max determinants would not consider a single limiting step in the oxygen pathway (e.g., Exerc Sport Sci Rev. 2000 Jan;28(1):10-4 and doi: 10.1146/annurev.ph.58.030196.000321)

> Response: We completely agree, and we apologize if these lines suggested that we would consider only a single limiting step for $\dot{V}O_{2max}$. See also the previous comment about the Wagner diagram. We have now revised this to underscore that mitochondria are not the sole determinant of $\dot{V}O_{2max}$, but rather can be predictive of maximal oxygen utilization, as they are the ultimate recipients. We find that more mitochondria correlate with higher maximal oxygen uptake. The lack of a relation between mitochondrial markers and $\dot{V}O_{2mas}$ in patients (but not in controls and after bed rest) indicates that other

factors contribute. We have also adjusted the manuscript to reflect that we cannot decipher the exact limitation.

The textual changes are the same as in the previous comment about the Wagner-diagram

181 Perhaps some of the latest concepts in intramyocyte oxygen movement should be considered (e.g., doi: 10.1007/s00421-021-04854-7).

Response: Yes, we agree. We have now incorporated some of these concepts within the text.

Lines 169-173:

"Given that physical activity has been shown to shift limitations of maximal aerobic capacity from oxygen utilization to oxygen supply[26] and given the association between mitochondrial function and whole-body aerobic exercise capacity[27], we assessed mitochondrial respiration of permeabilized muscle fibers and succinate dehydrogenase (SDH) activity in sections, to provide further insight into mitochondrial alterations upon bed rest and in patients."

Lines 177-184:

"As mitochondria are the ultimate recipients in the oxygen cascade, we hypothesized that either SDH activity or oxidative phosphorylation would correlate with $\dot{V}O_{2max}$. Indeed, both SDH activity and oxidative phosphorylation capacity were correlated with $\dot{V}O_{2max}$ in healthy controls (Figure 3C+D), and such a relation was present following bed rest. However, this association was absent in patients with long COVID and ME/CFS. While the precise limitation of $\dot{V}O_{2max}$ in patients is obscured, the lack of association of $\dot{V}O_{2max}$ with mitochondrial variables suggests that reductions in exercise capacity are not limited by mitochondrial density or respiratory capacity per se."

Lines 373-386:

"*Invasive CPET results indicate that at maximal exercise, arterial PO2 in long COVID and ME/CFS is similar to healthy people[38,39]. Intramyocyte oxygen movement is dependent on convective oxygen delivery, oxygen diffusion through the tissue, and mitochondrial oxygen consumption[59]. The decrease in capillary-to-fibre ratio for a given fiber size reduces the potential for oxygen convective delivery. While the reduced mitochondrial oxidative capacity was reduced in both patients with long COVID and ME/CFS, which would suggest an elevation in intracellular PO_2, we were not able to measure intracellular PO_2 directly. However, as myoglobin is an integral piece in intramyocyte oxygen diffusion, we suspected that if intracellular PO_2 was indeed higher and oxygen convective delivery was impaired, there may be a compensatory*

response of myoglobin in order to maintain the oxygen diffusion gradient. As myoglobin content was similar amongst the groups, it would suggest that intramyocyte diffusion capacity may be maintained. However, it is unknown whether any of hemoglobin, capillary or myocyte membrane function is altered, which also impacts oxygen diffusive capacity. Future work should aim to address whether membrane functions in these domains are altered and may contribute to impaired oxygen extraction."

And the new paragraph about ***Limitations to*** $\dot{V}O_{2max}$ on Lines 353-366. See above.

234 "hyperventilation" is defined as PCO2<40 mmHg. This was not measured nor were ventilatory patterns reported.

Response: We used the term hyperventilation based on the $\dot{V}CO_2$ and $\dot{V}E/\dot{V}CO_2$ responses. Since the definition of hyperventilation includes additional requirements, and we do not know the PCO_2 responses, we now refrain from using the term 'hyperventilation'. We have adjusted the text to reflect that the patterns we observed have also been observed in hyperventilation, and that we cannot conclude this is hyperventilation *per se*.

Lines 288-292:

"*While arterial PCO_2 was not measured, a lower VE_{max} and higher VE_{max}/VCO_{2max} and VE-VCO_2 slope at submaximal exercise suggests earlier indications of hyperpnea*[28-30] *in patients. The causes of these findings are currently unclear, however reduced blood or muscle CO_2 buffering capacity*[31]*, increased chemoreceptor sensitivity*[32]*, or pulmonary vascular dysfunction resulting in increased dead space ventilation*[33] *may contribute.*"

298-300 These ideas need better development.

304-306 Maybe in mice but are they in humans?

310 Be specific. What evidence for "local hypoxia" were you looking for?

303-312 Needs rewriting for clarity.

Response: We thank the reviewer for these four comments that we address together. After careful deliberation, we have removed the discussion pertaining to hypoxia, as we do not have enough proof to further substantiate these ideas. Rather, we have now included revisions regarding intramyocyte oxygen movement, and a new section regarding $\dot{V}O_{2max}$. limitations. These changes are now also in line with the reviewer's comments.

314- Please provide more evidence that fiber type transformation did or did not happen. Sometimes authors interpret smaller fiber XSA – and thus less total area of a given fiber type – as evidence for transformation when it is not.

Response: Yes, we agree. We have included a new figure (Supplemental Figure 7), in which fiber type proportions were calculated as numerical proportion of fibers of a given type relative to all fibers present. This shows similar patterns to the fiber type composition expressed as the cross-sectional area of each fiber type relative to the whole cross-sectional area suggesting fibers of all types exhibited a similar degree of atrophy. We are currently performing follow-up experiments to better understand the molecular pathways underlying this fiber type shift. This is however outside the scope for this manuscript.

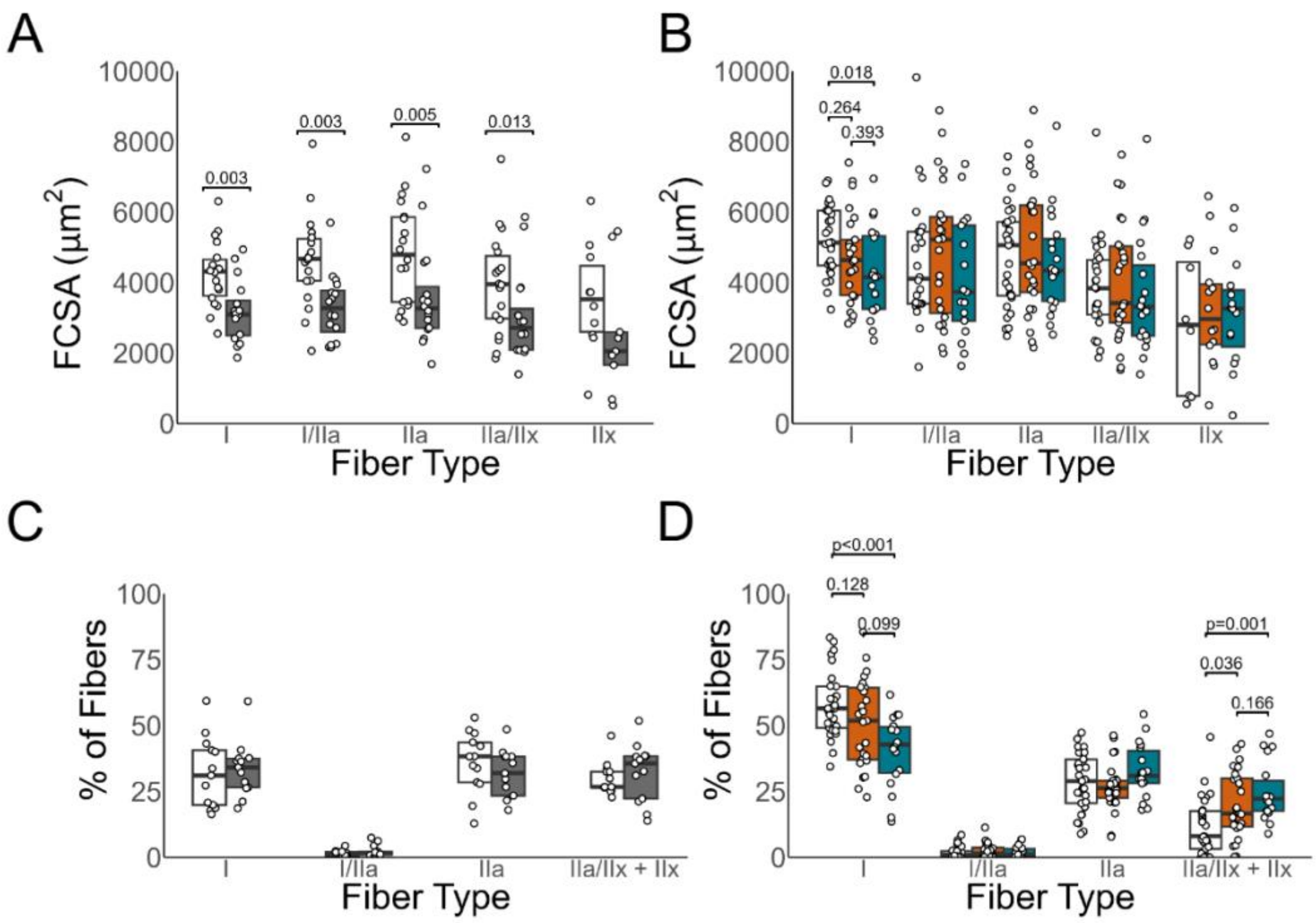

***Supplementary Figure 7**: Average fiber cross-sectional area (FCSA) for each individual fiber type was reduced from pre- to post-bed rest (**A**), with the exception of the few pure type IIx fibers. Type I fiber FCSA was significantly lower in patients with ME/CFS (**B**, green) compared to healthy controls, while patients with long COVID (orange) were not significantly different from healthy controls. Fiber type was subsequently calculated as the numerical percentage relative to all fiber number (**C**,**D**). Fiber type distribution did not change after long-term bed rest (**C**). Patients with ME/CFS had lower proportions of type I fibers compared to healthy controls and tended to be lower than patients with long COVID (**D**, P=0.099). Both patients with long COVID and ME/CFS displayed higher proportions of type IIa/IIx hybrid fibers*

than healthy controls. After Box-Cox transformations, data for FCSA of type I, type I/IIa and IIa fibers were normally distributed, while data for FCSA of type IIa/IIx and IIx fibers remained non-normally distributed. Data for percentage of type I in bed rest, percentage of I/II hybrids in all groups, and percentage of IIa fibers in all groups were normally distributed after Box-Cox transformations; all other data was non-normally distributed. Comparisons between pre- and post- bed rest were assessed using paired t-tests for parametric data and Mann–Whitney U test for non-parametric data. Comparisons between patients with long COVID and patients with ME/CFS and healthy controls were assessed using ANOVA, with Tukey HSD post-hoc testing for parametric data or Kruskal–Wallis H test, with pairwise Wilcoxon tests with Benjamini–Hochberg correction post-hoc for non-parametric data.

Reviewer #2 (Remarks to the Author)

The study aimed to compare the exercise responses and skeletal muscle adaptations in long COVID and ME/CFS patients with those following 60-day strict bed rest. The hypothesis was that while aerobic capacity would be similarly reduced in patients and after bed rest, skeletal muscle adaptations would differ significantly. Patients were expected to show no muscle atrophy but reduced capillary-to-fiber ratios and densities, whereas bed rest was anticipated to lead to severe muscle atrophy and altered capillary ratios.
Key hypotheses and focuses:
i) Comparison of CPET responses and muscle adaptations in patients with long COVID and ME/CFS and Controls with those under strict bed rest.
ii) Expectation of differing skeletal muscle markers between patients and bed rest participants.
iii) Examining associations between muscle markers and exercise capacity across these groups through aerobic exercise responses and muscle biopsies.

What are the noteworthy results?
The remarkable results relate to the fact that a 60-day bed rest period was used as a time window for deconditioning effects. This is an exceptional approach. Furthermore, the muscle physiological/pathophysiological response to exercise was compared between long COVID, ME/CFS, healthy controls, and bed rest controls with remarkable experimental quality.
Will the work be of significance to the field and related fields?
The study will be of importance to the field and related disciplines in the area of post-acute infectious syndromes. According to current knowledge, no comparable protocols exist in the literature for the combination of bed rest and post-infectious diseases.

Does the study support the conclusions and claims, or is additional evidence needed?

The study's results indicate that deconditioning is not the sole factor explaining post-exercise-induced symptom exacerbation. On the other hand, the study cannot separate the contribution of deconditioning from other proven physiological factors.

Response: We thank the reviewer for his/her positive comments about the design and experimental approach, and relevance for the field.

Are there any deficiencies in the data analysis, interpretation, and conclusions? Do these preclude publication or require revision?
The following revisions (adjustments) are required:
i) Clinical case definition: The clinical case definition for ME/CFS needs to be clarified, as the ME/CFS diagnosis was made before 2020. Report the following aspects: type of infection (respiratory tract, EBV, gastroeneritis, inflamed bowel disease, rheumatological aspects, autonomic symptoms, e.g., POTS, dyspnea).

Response: We have now included this data in Supplemental Figure 1 (provided below). As there remains some recall bias with ME/CFS diagnosis, a majority of our cohort experienced an unknown onset, but do recall a particular point in time. This is consistent with the Canadian Consensus Criteria.

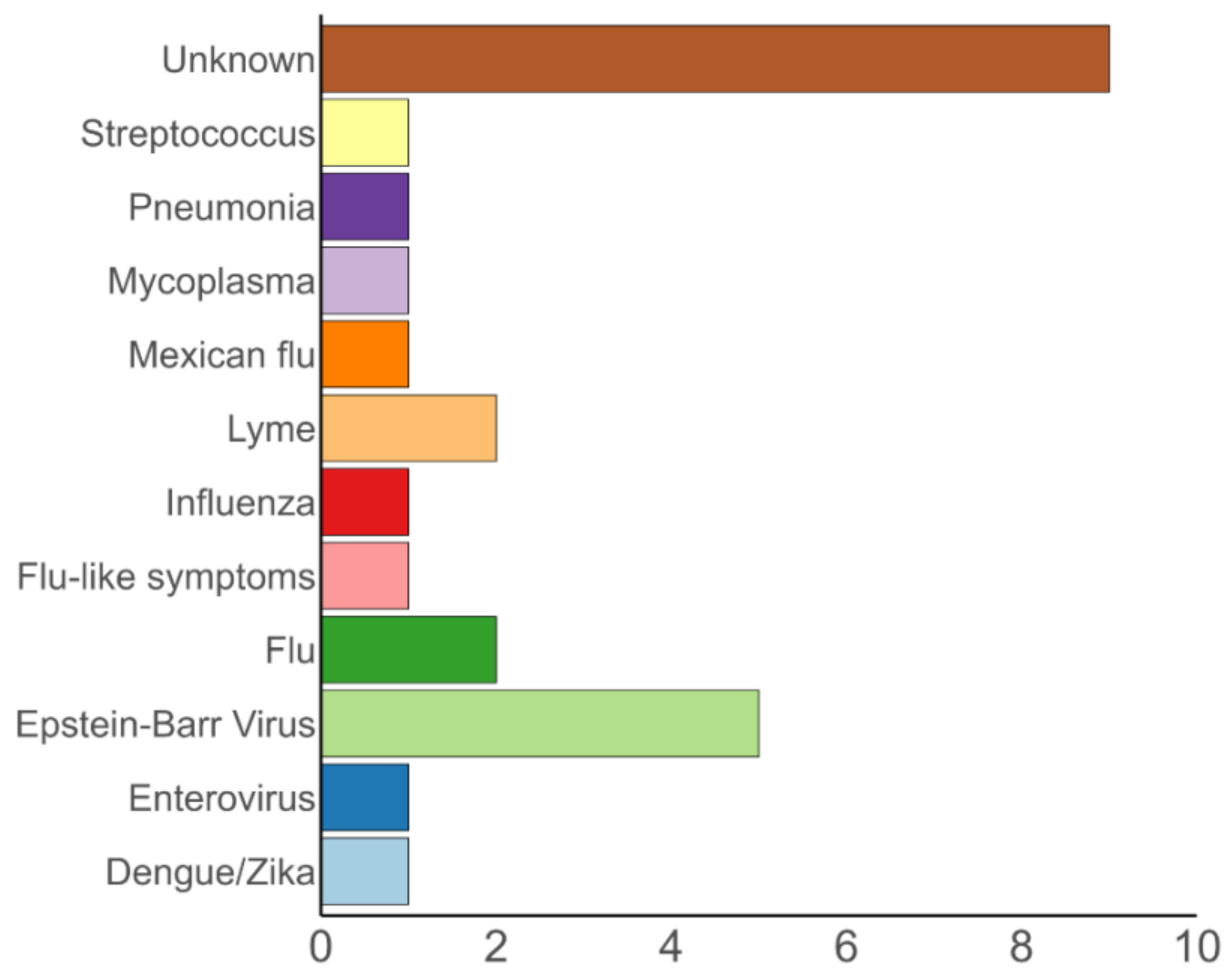

***Supplemental Figure 1**: Infection type prior to ME/CFS diagnosis. All ME/CFS diagnoses were made prior to 2020. Bars display the total number of responses.*

ii) For both groups (long COVID and ME/CFS): Report the frequency and severity of symptoms and the DSQ items in a table. This presents an opportunity for the paper, as Q9, for example, allows for differentiation between < 14 hours and > 14 hours (e.g., Kedor et al. 2022, https://doi.org/10.1038/s41467-022-32507-6). The data analysis should be expanded to include, for example, correlative measures (e.g., VO2-heart rate slope).

Response: We have now included the DSQ-PEM questionnaire data (see Supplemental Figure 2, provided below). While we agree that a correlation between Q9 would perhaps aid to tease out differences in severity, our cohort appeared to have been nearly uniform in their responses, and thus a correlation analysis with these questionnaire data is not possible.

A

		CON	LC	ME/CFS
Frequency	DSQ-PEM1	0 (0,1)***, †††	4 (1,4)**	4 (2,4)
	DSQ-PEM2	0 (0,1)***, †††	3 (0,4)	3.5 (1,4)
	DSQ-PEM3	0 (0,0)***, †††	3 (2,4)	3 (1,4)
	DSQ-PEM4	0 (0,0)***, †††	3 (0,4)*	3 (1,4)
	DSQ-PEM5	0 (0,0)***, †††	3 (1,4)	3 (2,4)
Severity	DSQ-PEM1	0 (0,1)***, †††	3 (1,4)***	3 (3,4)
	DSQ-PEM2	0 (0,1)***, †††	2 (0,3)**	3 (1,4)
	DSQ-PEM3	0 (0,0)***, †††	3 (1,4)	3 (1,4)
	DSQ-PEM4	0 (0,0)***, †††	2 (0,4)**	3 (1,4)
	DSQ-PEM5	0 (0,0)***, †††	3 (1,4)*	3 (2,4)

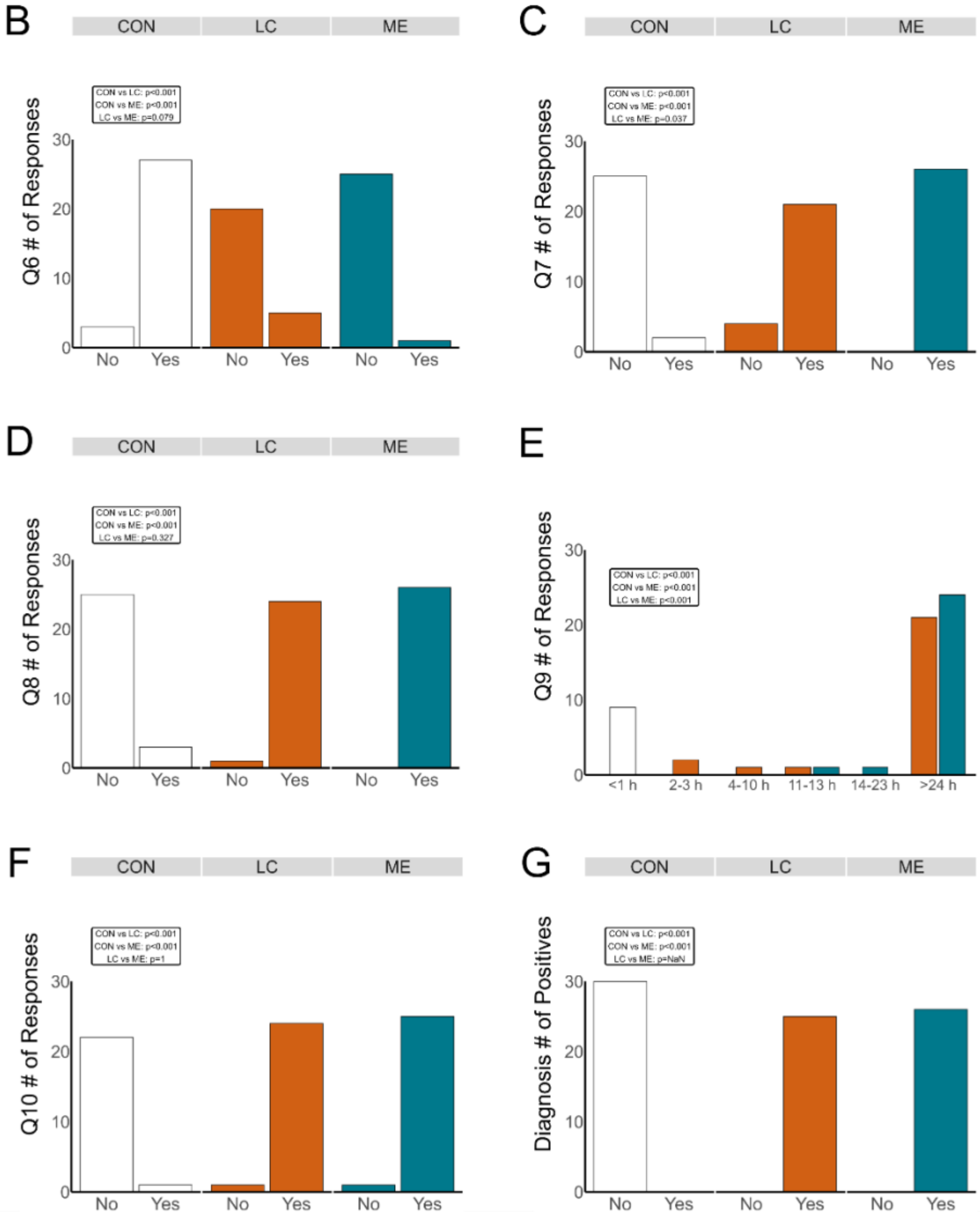

***Supplemental Figure 2**: DePaul Symptom Questionnaire-Post-exertional Malaise (DSQ-PEM) responses. Patients with long COVID and ME/CFS reported significantly higher DSQ-PEM severity and frequency scores (**A**) compared to healthy controls. Patients with long COVID and ME/CFS responded more negatively to questions 6-8 and 10 of the DSQ-PEM (**B-D,F**) and indicated longer durations of feeling worse following activities (**E**) compared to healthy controls. All patients with long COVID and ME/CFS had a positive PEM diagnosis using the DSQ-PEM (**G**), while no controls were positive for PEM. Responses in A are displayed as median (minimum, maximum). Comparisons were made using a Kruskal–Wallis H test, with pairwise Wilcoxon tests with Benjamini–Hochberg correction post-hoc. **A**: *p<0.05, **p<0.01, ***p<0.001. **B-G**: *: Compared to ME/CFS, †: Compared to long COVID.*

iii) Please report the lactate values at all sampling times (e.g., in Supplementary Figure 2).

Response: We have added this data in Supplemental Figure 13A (provided below).

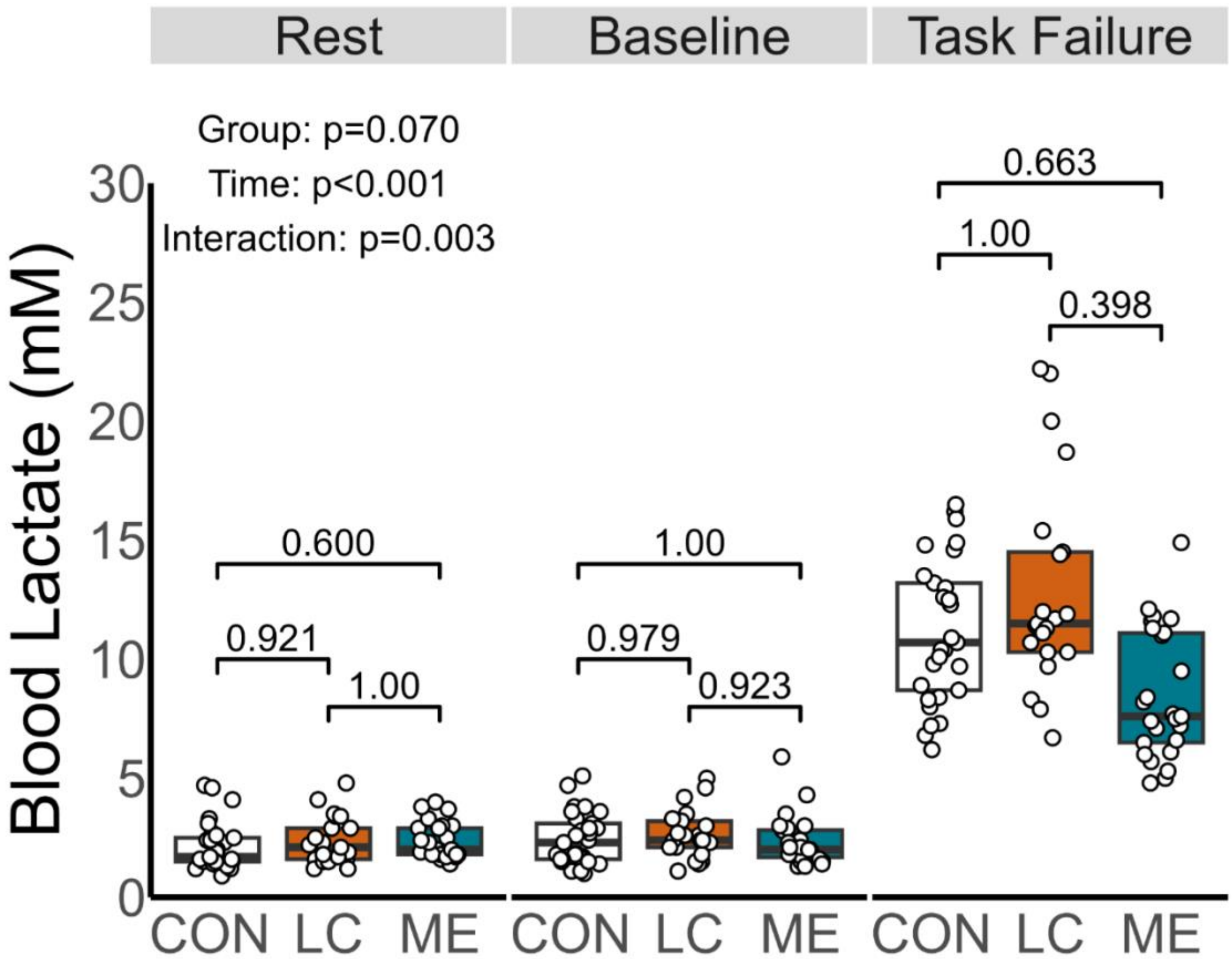

***Supplemental Figure 13**: Blood lactate measurements of healthy controls (CON, n=29), patients with long COVID (LC, n=21), and patients with ME/CFS (ME, n=24) before and after maximal cardiopulmonary exercise testing. All groups showed elevations of blood lactate at task failure. No differences were found between rest and baseline in any group. Neither patient group was significantly different from healthy controls at any timepoint. After Box-Cox transformations, data at task failure was non-normally distributed. Data was compared using a repeated measures aligned rank transformation test with pairwise estimated marginal means post-hoc for interaction effects.*

iV) The muscle-associated analyses are excellent and show that deconditioning alone cannot explain PEM. This is very valuable and helpful. It is very helpful if you can summarize the pathophysiological changes (respiratory, cardiovascular, muscle physiological) in the discussion with regard to the following aspects: finding, pathophysiological interpretation concerning deconditioning and other explanations (e.g. Haunhorst et al. 2025, PMCID: PMC11825644).

Response: We have now incorporated a short section on other potential pathological factors.

Lines 409-417:

"Shared pathophysiology of long COVID and ME/CFS

Previous studies have indicated an overlap in potential pathological mechanisms in both long COVID and ME/CFS, that include (but certainly not limited to) autonomic dysfunction, microclot formation in the blood, endothelial dysfunction and mitochondrial dysfunction[67]. In our current study, we provide evidence for skeletal muscle mitochondrial impairments to be implicated in both long COVID and ME/CFS. However, as both diseases are heterogenous, other mechanisms may be involved depending on clinical presentation. In our current cohort, we focused on long COVID patients that exhibited PEM as a key symptom. Thus, it may be that the mitochondrial dysfunction we highlight is more unique to those patients that exhibit PEM rather than other long COVID symptoms.

The source code is available as an R script as supplementary material to the document. Please add a README file with sufficient instructions for installation and execution. The code and all necessary notes are available at https://github.com/bcn95/LC_ME_CFS_BED_REST. The code can be installed and executed.

Response: Thank you. A README file is indeed added.

Reviewer #3 (Remarks to the Author):

Determining how much of the exertional intolerance characteristic of ME/CFS and Long COVID is related to simple deconditioning vs. disease is critical. The study aims to determine if decreased exercise capacity in both diseases can be linked to intrinsic skeletal muscle abnormalities that differ from those found in de-training. This represents an important step in the field as post exertional malaise precludes traditional exercise rehabilitation approaches that have been found to be useful in deconditioning.
The authors' key findings suggest bed rest caused muscle atrophy and reduced oxidative phosphorylation, while patients did not show muscle atrophy but had fewer capillaries and more glycolytic fibers.

Response: We thank the reviewer for the positive comments.

The presentation of the data could be improved with some minor modifications:

-bedrest subjects was 66% male whereas healthy controls and patients was 50% male. The absolute VO2 max for males will be higher than females. VO2 max comparisons among groups should be expressed as percent of predicted, whose independent variables are driven by age, sex, and height. Ideal matching of VO2 max would be as a percent of predicted not absolute.

Response: We have now included the $\dot{V}O_{2max}$ data as a percentage of predicted. We have further adjusted the $\dot{V}O_{2max}$ and GET to be relative to body weight, as this better reflects relationships with ex vivo measures, as they were normalized to either tissue mass or tissue thickness.

We have performed additional analyses of age- and sex-matched bed rest participants (see Supplemental Figure 12, provided below), which shows similar results as our complete cohort. While this sub-analysis was exploratory and underpowered, we find that the conclusions are consistent with the results of the complete cohort.

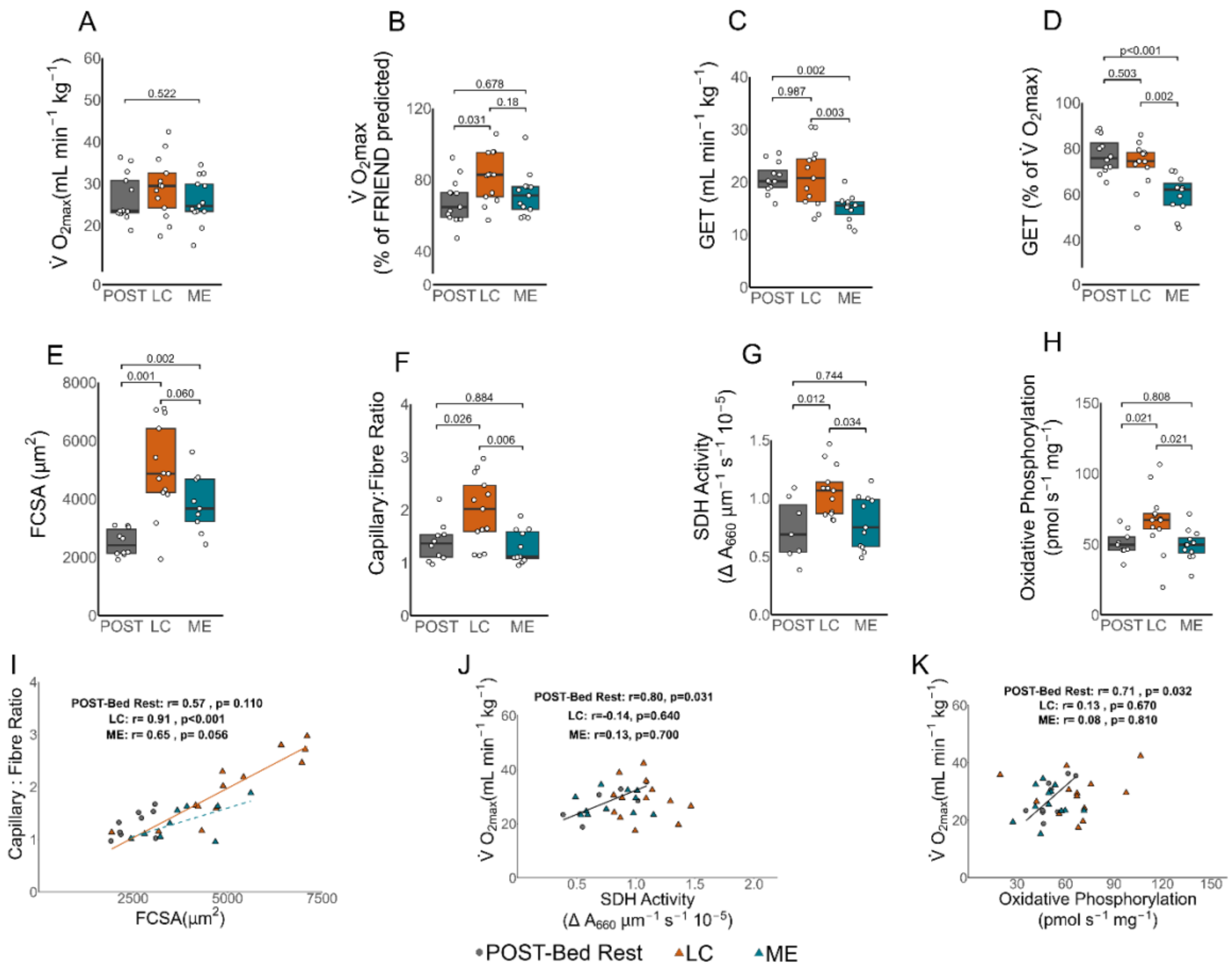

Supplemental Figure 12*: A sub-analysis was conducted on age- and sex-matched individuals between post-bed rest (n=13), patients with long COVID (LC, n=13) and patients with ME/CFS (n=13). There were no differences in* $\dot{V}O_{2max}$ *between groups (****A****), however patients with long COVID had significantly higher % of predicted* $\dot{V}O_{2max}$ *based on the FRIEND equation (****B****). The gas exchange threshold was lower in patients with ME/CFS than patients with long COVID (****C****). The gas exchange threshold relative to* $\dot{V}O_{2max}$ *was also earlier in patients with ME/CFS compared to post-bed rest and patients with long COVID (****D****). Skeletal muscle fiber cross-sectional area (FCSA) was lower after bed rest compared to either ME/CFS or long COVID, while there was a trend for patients with ME/CFS to present with smaller fibers than patients with long COVID (****E****). Capillary-to-fiber ratio was higher in long COVID than either post-bed rest or patients with ME/CFS (****F****). Similarly, succinate dehydrogenase (SDH) activity (****G****) and oxidative phosphorylation capacities (****H****) were higher in patients with long COVID than either post-bed rest or patients with ME/CFS. All groups had, or tended to have, a significant relation between FCSA and capillary-to-fiber ratio (****I****). There was a correlation between* $\dot{V}O_{2max}$ *and either SDH activity (****J****) or oxidative phosphorylation capacity (****K****) in participants after bed rest, but not in either patient group. Data from* $\dot{V}O_{2max}$*, gas exchange threshold relative to* $\dot{V}O_{2max}$*, FCSA, and oxidative*

phosphorylation remained non-normally distributed after Box-Cox transformations. Parametric comparisons were assessed using ANOVA, with Tukey HSD post-hoc testing. Non-parametric comparisons were assessed using Kruskal–Wallis H test, with pairwise Wilcoxon tests with Benjamini–Hochberg correction post-hoc. Solid lines represent significant correlations, dashed lines represent correlations with P<0.10.

-the asymmetric sex distribution across groups and its influence on whole body exercise and muscle metabolism should be discussed and alluded to in the limitations section.

Response: We had highlighted this limitation in the "Results" section, that although the disease cohort was age- and sex-matched (See Table 1, each group is 50% female), the bed rest cohort contained significantly fewer females. This was why the sub-group analysis between cohorts were included in the results section (Supplemental Table 1 and Supplemental Figures 4 and 12, provided below), in which patients and bed rest participants were age- and sex-matched retrospectively, creating an equal divide amongst males and females (13 participants per group, 7 females per group), and where healthy controls and pre-rest participants were similarly matched (17 participants per group, 7 females per group).

We have now added a limitation particular to the bed rest cohort to the discusison, in which we concede that sex differences in the bed rest study may exist, but the cohort was not powered to elucidate such differences.

Lines 442-443:

"While we attempted to account for any sex differences, we cannot exclude any sex-specific alterations that may occur during bed rest, due to an underpowered comparison."

We have added the following table and figures to the manuscript to provide relevant information related to sex and age-differences between patients and controls.

Supplemental Table 1*: Sub-cohort characteristics with both cohorts now matched for age and sex. Data presented as median (IQR). All data remained non-normally distributed following Box-Cox transformations. Group differences were compared using a Kruksal-Wallis test, with pairwise Wilcoxon signed-rank test post-hoc where applicable. ME/CFS: myalgic encephalomyelitis/chronic fatigue syndrome.*

	Bed rest	Healthy Control	Bed rest	Long COVID	ME/CFS

	Pre		Post		
**Participants (# Females)**	17 (7)	17 (7)	13 (7)	13 (7)	13 (7)
**Age (years)**	34 (29-43)	34 (29-42)	29 (27-40)	31 (28-42)	32 (28-42)
**Height (cm)**	175 (172-179) *	180 (176-186)	174 (162-175)	177 (168-191)	175 (172-186)
**Weight (kg)**	76 (69-80)	74 (63-86)	70 (66-76)	82 (71-90)	84 (75- 86)
**Symptom Duration (days)**	n/a	n/a	n/a	500 (408-599) †††	3793 (2463-4613)
**Daily Steps (steps*day^{-1})**	ND	7011 (4770-8316)	0	3970 (2794-5030)	3827 (2722-4901)

***: *vs.* healthy control; †: *vs.* ME/CFS; §: *vs.* Bed rest**

***: P<0.05; **: P<0.01; ***: P<0.001**

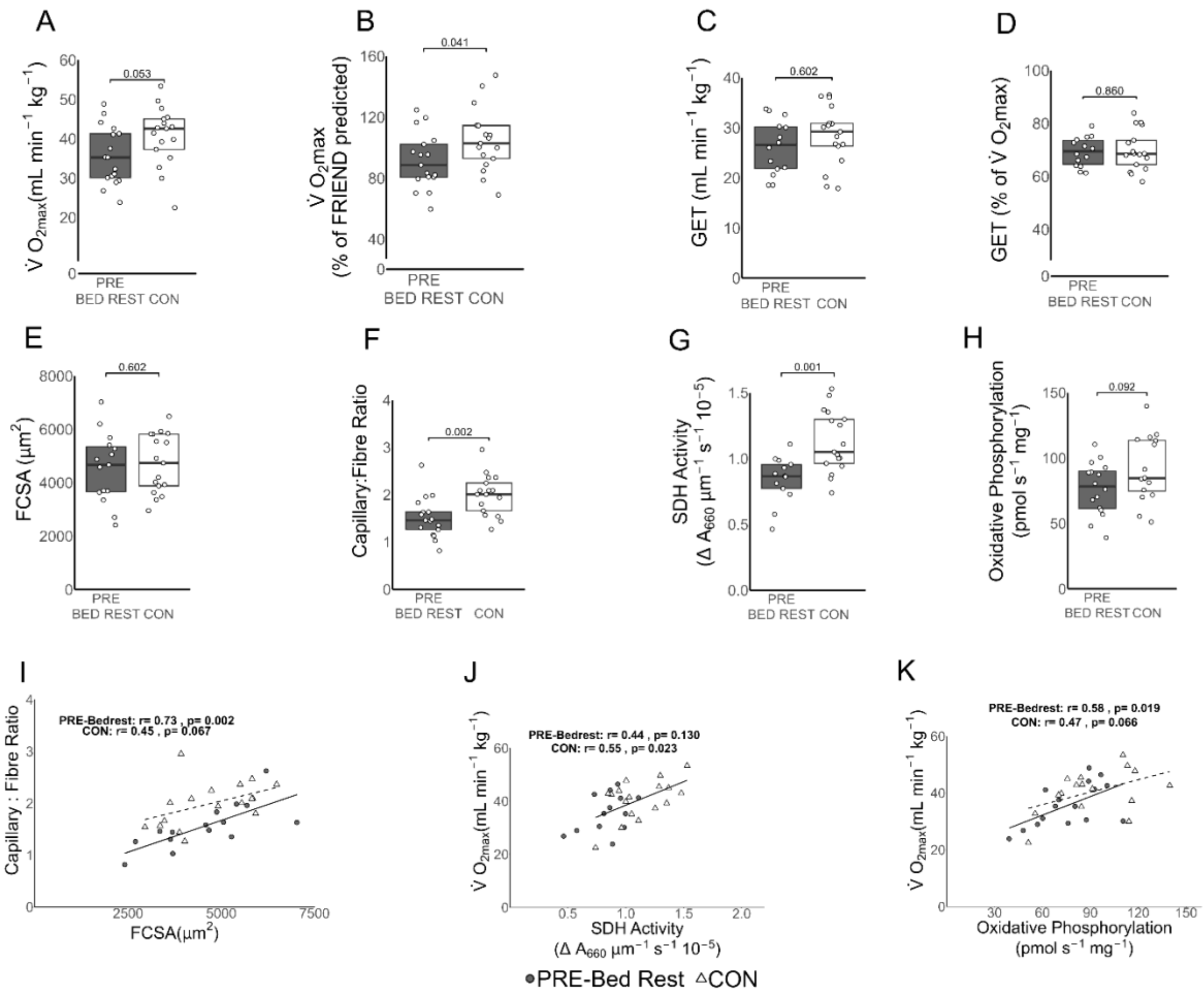

***Supplemental Figure 4**: A sub-analysis was conducted on age- and sex-matched individuals between healthy individuals pre-bed rest (n=17) and healthy individuals matched to patients with long COVID and ME/CFS (n=17; Supplemental Table 1). There was a tendency for $\dot{V}O_{2max}$ to be lower in bed rest participants compared to the patient-matched healthy controls (**A,** P=0.053) when expressed relative to body weight, and a significant difference when expressed as % of predicted $\dot{V}O_{2max}$ based on the FRIEND equation (**B**). However, there were no differences in in gas exchange threshold (GET) (**C**), the occurrence of the gas exchange threshold relative to $\dot{V}O_{2max}$ (**D**) or fiber cross sectional area (FCSA, **E**) between groups. Participants before bed rest had a lower capillary-to-fiber ratio (**F**), lower succinate dehydrogenase (SDH) activity (**G**), and oxidative phosphorylation capacity (**H**) than patient-matched healthy controls. Both groups had, or tended to have a significant relation between FCSA and capillary-to-fiber ratio (**I**). Only patient-matched healthy controls had a significant relation between $\dot{V}O_{2max}$ and SDH activity (**J**). Both groups had, or tended to have, a significant association between $\dot{V}O_{2max}$ and oxidative phosphorylation capacity (**K**). Data for $\dot{V}O_{2max}$, FCSA, and SDH activity remained non-normally distributed after Box-Cox transformations. Parametric comparisons were assessed using non-paired t-*

tests. Non-parametric comparisons were assessed using Wilcoxon signed rank tests. Solid lines represent significant correlations, dashed lines represent correlations with P<0.10.

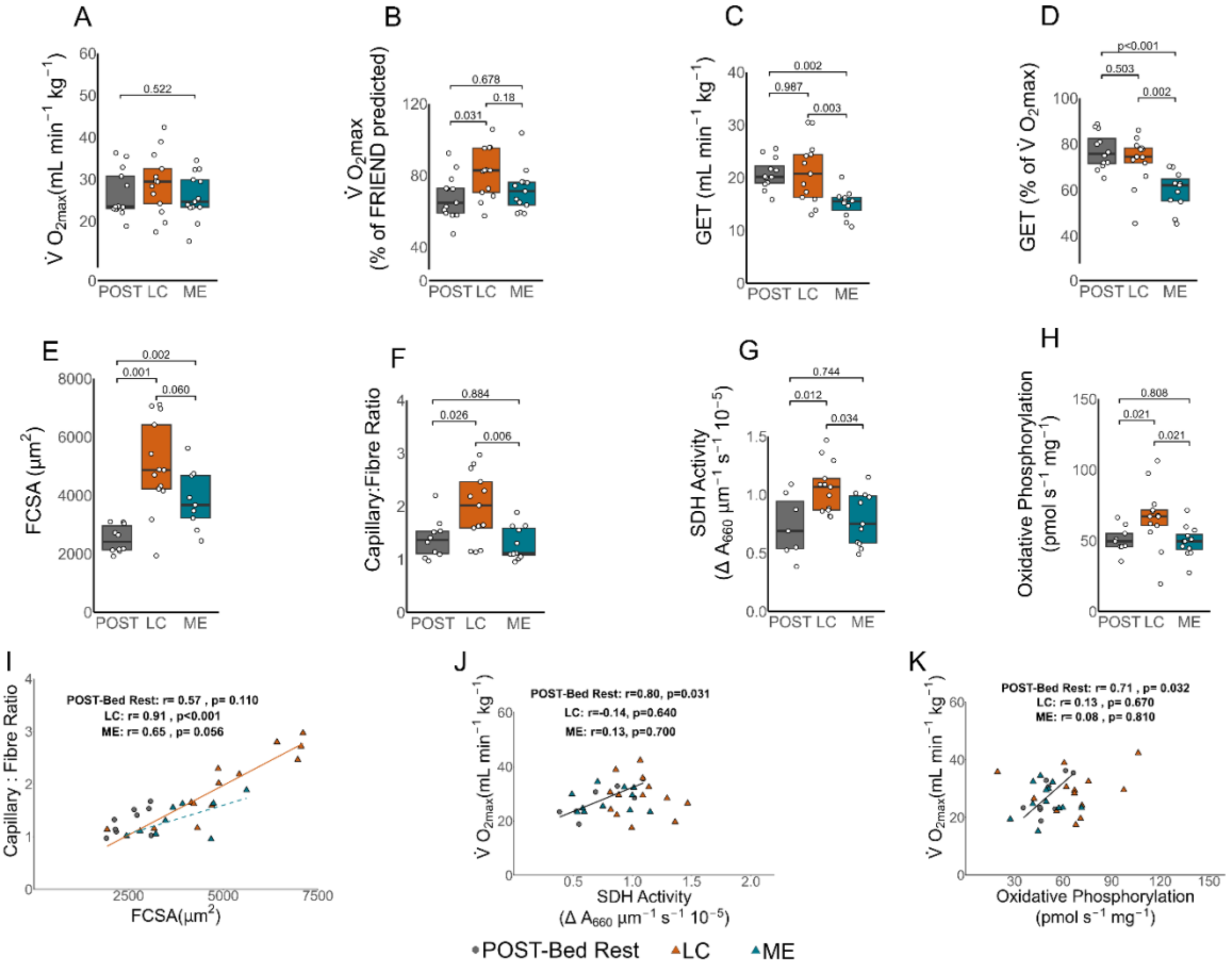

***Supplemental Figure 12**: A sub-analysis was conducted on age- and sex-matched individuals between post-bed rest (n=13), patients with long COVID (LC, n=13) and patients with ME/CFS (n=13). There were no differences in $\dot{V}O_{2max}$ between groups (**A**), however patients with long COVID had significantly higher % of predicted $\dot{V}O_{2max}$ based on the FRIEND equation (**B**). The gas exchange threshold was lower in patients with ME/CFS than patients with long COVID (**C**). The gas exchange threshold relative to $\dot{V}O_{2max}$ was also earlier in patients with ME/CFS compared to post-bed rest and patients with long COVID (**D**). Skeletal muscle fiber cross-sectional area (FCSA) was lower after bed rest compared to either ME/CFS or long COVID, while there was a trend for patients with ME/CFS to present with smaller fibers than patients with long COVID (**E**). Capillary-to-fiber ratio was higher in long COVID than either post-bed rest or patients with ME/CFS (**F**). Similarly, succinate dehydrogenase (SDH) activity (**G**) and oxidative phosphorylation capacities (**H**) were higher in patients with long COVID than either post-bed rest or*

*patients with ME/CFS. All groups had, or tended to have, a significant relation between FCSA and capillary-to-fiber ratio (**I**). There was a correlation between $\dot{V}O_{2max}$ and either SDH activity (**J**) or oxidative phosphorylation capacity (**K**) in participants after bed rest, but not in either patient group. Data from $\dot{V}O_{2max}$, gas exchange threshold relative to $\dot{V}O_{2max}$, FCSA, and oxidative phosphorylation remained non-normally distributed after Box-Cox transformations. Parametric comparisons were assessed using ANOVA, with Tukey HSD post-hoc testing. Non-parametric comparisons were assessed using Kruskal–Wallis H test, with pairwise Wilcoxon tests with Benjamini–Hochberg correction post-hoc. Solid lines represent significant correlations, dashed lines represent correlations with P<0.10.*

-there is a wide range of daily steps and absolute VO2 max in patients; it would be important to know how much of that variance is related to sex, training status and the disease itself.

Response: We indeed agree that there is substantial heterogeneity with both daily steps and $\dot{V}O_{2max}$. Unfortunately, due to lack of information regarding pre-disease training status and physical activity levels, we are unable to dissect the influence of previous training status on either daily steps or $\dot{V}O_{2max}$. We now acknowledge this in the limitations section.

Lines 432-436:

"*There was likely self-selection bias towards patients with milder symptoms, as they volunteered to travel to the laboratory for multiple occasions and undergo a maximal exercise test. Our results are therefore not directly applicable to home-bound patients[68]. Similarly, we were unable to account for training status before disease onset. We do not know if previous training status influences susceptibility of skeletal muscle alterations[69,70].*

We have included additional analyses correcting for sex and age for both daily steps and $\dot{V}O_{2max}$ in patients. With respect to $\dot{V}O_{2max}$ measures, we conducted an analysis including sex, age, height and weight (as all are known to influence $\dot{V}O_{2max}$) in addition to condition. For $\dot{V}O_{2max}$ (L/min), $\dot{V}O_{2max}$ (ml/kg/min), % of predicted $\dot{V}O_{2max}$ (Wasserman equation), there was only a tendency for a sex effect (p=0.057) for Wasserman predicted. All $\dot{V}O_{2max}$ measures yielded an age effect, however none of these measures displayed group-by-age or group-by-sex interactions. Only $\dot{V}O_{2max}$ (L/min) shows a group-by-age effect, while both the Wasserman and FRIEND predicted equations yield tendencies towards a group-by-sex interaction (p=0.056 for Wasserman and p=0.088 for FRIEND). Graphs are provided below to illustrate this.

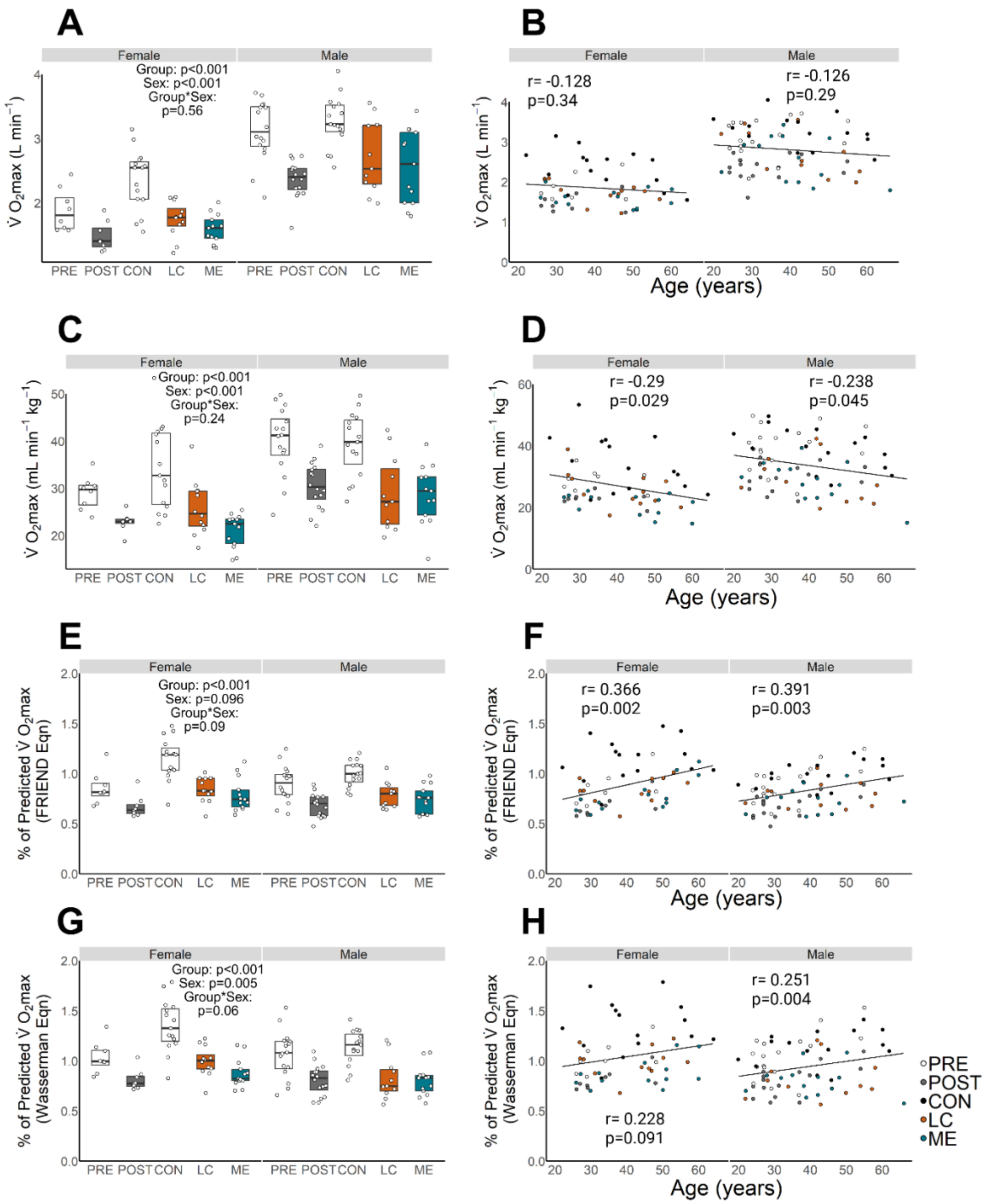

Males had higher absolute measures of $\dot{V}O_{2max}$ (A, L/min) and normalized to body weight (C, mL/kg/min). Using the FRIEND equation (E), females tended to achieve higher than their age- and sex- predicted $\dot{V}O_{2max}$, regardless of group. Similarly, using the Wasserman equation (G), females did achieve higher percent-predicted $\dot{V}O_{2max}$. While absolute $\dot{V}O_{2max}$ measures (B) did not relate to age in either males or females, relative $\dot{V}O_{2max}$ (D) was negatively associated with relative $\dot{V}O_{2max}$ measures in both males and females. Both the FRIEND and Wasserman percent-predicted $\dot{V}O_{2max}$

measures either related or tended to relate positively to age in both males and females.

With respect to daily steps, a sex-by-group interaction was observed, driven by a higher step count in healthy women relative to all groups, while the daily step count in healthy control men was not higher than men with long COVID or ME/CFS (p=0.394 vs LC and p=0.149 vs ME). However, step counts provide only a partial proxy for habitual physical activity, as they do not capture non-locomotive activities such as cycling or resistance exercise, nor do they reflect historical training status. While daily steps were related to $\dot{V}O_{2max}$, this measure does not allow us to disentangle the relative contributions of sex, habitual activity patterns, and disease-related limitations to the observed heterogeneity in $\dot{V}O_{2max}$. Accordingly, variation in daily steps should be interpreted as an incomplete indicator of activity status rather than a mechanistic explanation for between-group differences.

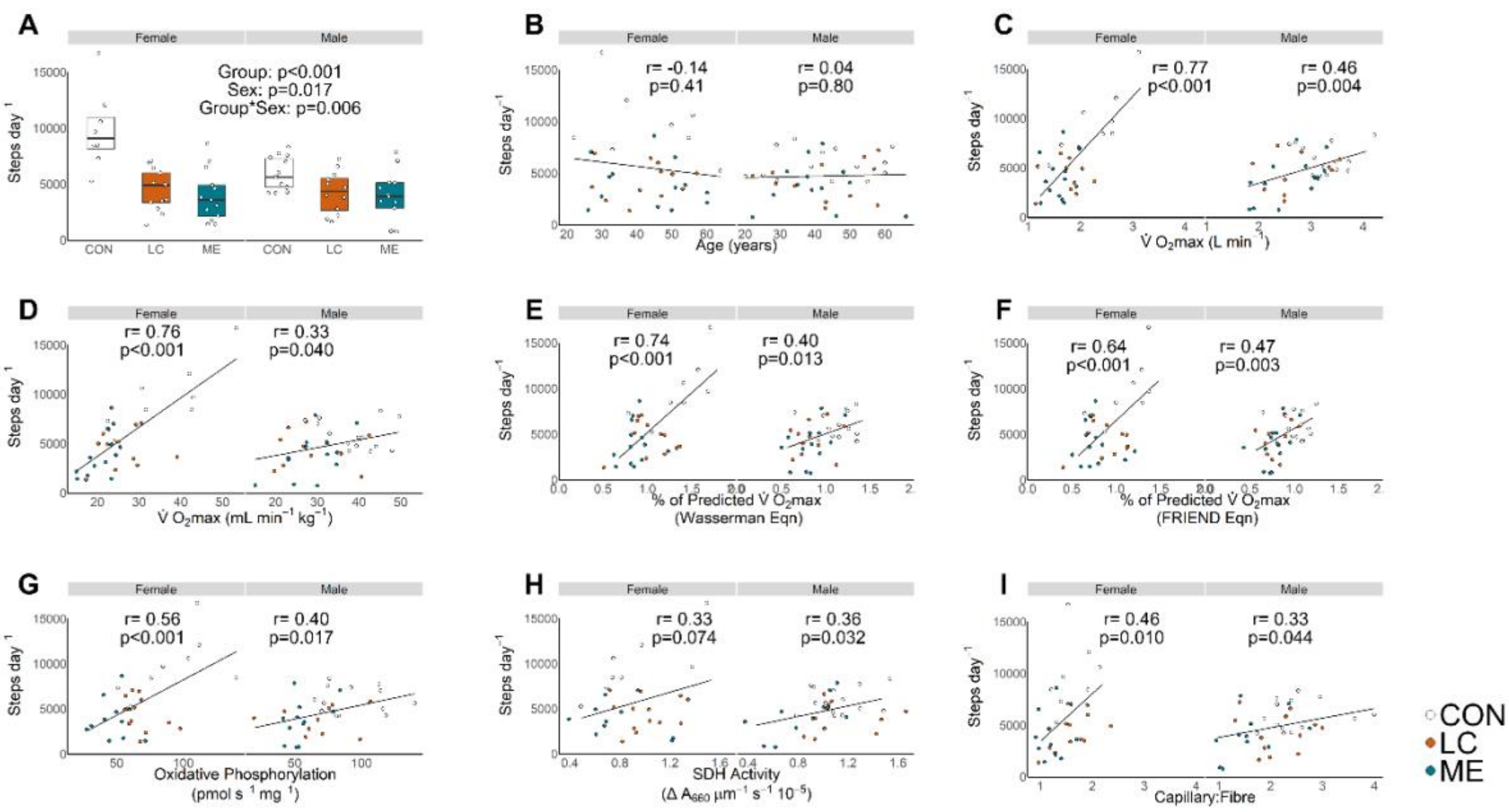

Daily step counts showed a Group x Sex interaction (A), in which female controls were significantly higher than all other groups. Daily steps did not correlate with age (B), but did correlate with measures of $\dot{V}O_{2max}$ (C-F). There were modest correlations between daily step counts and mitochondrial measures (G+H), but no relation with capillary:fibre ratio (I).

-the influence of the head down position during bed rest should be discussed as a potential confounder (any deconditioning in patients would not be related to that body position). Dysautonomia is part of ME and LC exercise intolerance and arterial baroreceptors are reset according to body position.

Response: We agree that the head-down position is less ideal than a horizontal bed rest. There have been some bed rest studies with a horizontal design, but we do not have indications that the skeletal muscle alterations are significantly different from those observed in our bed rest cohort. As such, there are no indications that there are significant differences in both bed rest models. Head-down tilt bed rest induces a slightly greater cephalic fluid shift than horizonal bed rest, but there are currently no clear side-by-side comparisons with both bed rest models. The fluid shift has an immediate impact on the cardiovascular function, as plasma volume significantly decreases after long-term bed rest. We have now highlighted this in several sections, including the limitations.

Lines 298-299:

"The increase in both resting heart rate and maximal heart rate are likely reflective of a decreased stroke volume[37], potentially due to the reduced plasma volume observed after bed rest[37]. "

Lines 443-447:

"A further limitation is the use of head-down tilt bed rest as a comparator for clinical deconditioning. Although this model enables long-term experimental control of physical inactivity, it introduces haemodynamic alterations that differ from those experienced by ambulatory patients, particularly with respect to fluid redistribution and orthostatic loading. As such, conclusions drawn from the bed-rest comparison are most robust for skeletal muscle structural and metabolic outcomes, whereas cardiovascular interpretations are necessarily more constrained. Additionally, head-down tilt bed rest may differently affect women due to sex-specific hormones on blood pressure regulation[71]."

-lines 236 and 237: increased physiologic dead space ventilation does not lead to hyperventilation, rather it leads to hyperpnea; this should be so indicated. Respiratory muscle weakness does not lead to hyperventilation or hyperpnea.

Response: We have adjusted the text to more adequately reflect that these phenomena do not necessitate hyperventilation. We have further removed weak respiratory muscles as a contributor, as this was an erroneous statement.

Lines 288-292:

"While arterial PCO_2 was not measured, reductions in $\dot{V}E_{max}$ and increases in $\dot{V}E_{max}/\dot{V}CO_{2max}$ and the $\dot{V}E - \dot{V}CO_2$ slope are consistent with responses typically seen during hyperventilation[29–31], or hyperpnea. The causes of these findings are currently unclear, however reduced blood or muscle CO_2 buffering capacity[32], increased chemoreceptor sensitivity[33], or pulmonary

vascular dysfunction resulting in increased dead space ventilation[34] may contribute."

-264-274: the relationships between muscle mitochondrial density/ function vs. VO2 max in patients would be strengthened by expressing VO2max as a percent predicted. This would decrease the confounding effects of sex and body size.

Response:

We agree with the reviewer that expressing $\dot{V}O_{2max}$ as a percentage of predicted is appropriate for comparing aerobic capacity across individuals differing in age and sex, and we have included % predicted $\dot{V}O_{2max}$ for group-level comparisons. However, for analyses relating $\dot{V}O_{2max}$ to cellular and tissue-level markers such as SDH activity and oxidative phosphorylation capacity, we believe absolute or mass-normalized $\dot{V}O_{2max}$ measures are more physiologically appropriate.

Percent-predicted $\dot{V}O_{2max}$ incorporates age- and sex-specific population expectations, whereas SDH activity and mitochondrial respiratory capacity are absolute properties of skeletal muscle tissue and are not expressed relative to predicted values. As a result, correlating % predicted $\dot{V}O_{2max}$ with absolute mitochondrial markers risks introducing age/sex-related scaling effects that are not intrinsic to the muscle measurements themselves. For example, identical absolute $\dot{V}O_{2max}$ values can correspond to very different percentages of predicted $\dot{V}O_{2max}$ depending on age and sex, despite equivalent tissue-level oxidative capacity.

We therefore used $\dot{V}O_{2max}$ expressed relative to body mass (mL/kg/min) which aligns dimensionally with mitochondrial measures normalized to tissue mass and/or area, for structure–function analyses. At present, predicted reference values do not exist for SDH activity or mitochondrial respiratory capacity, precluding a fully "predicted-to-predicted" comparison.

The relevant analyses are displayed in the Figure below.

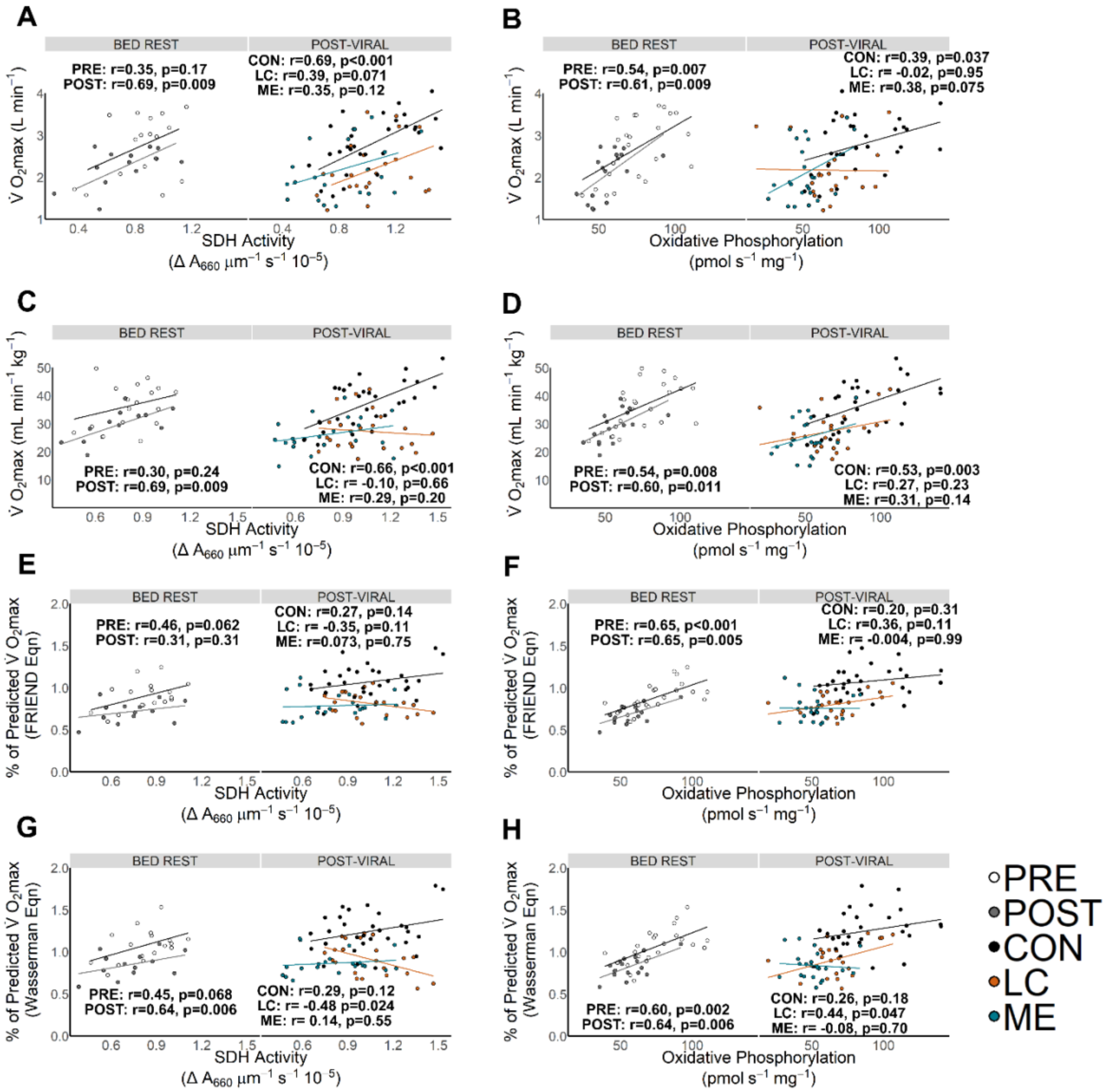

-461: your group has nicely shown alterations in skeletal muscle mitochondrial function as a result of prior exercise and PEM in the patient group. Please indicate when your muscle biopsies were obtained related to the CPET and what was done to assure that patients were biopsied while in a "steady state" not during an episode of PEM.

Response: We have clarified that the biopsies were obtained 6-days prior to the exercise testing. Unfortunately, we are unable to account for all previous PEM episodes, as triggers vary by individual, however we instructed all participants to refrain from activity at least 24-hours prior to the biopsy. We also anecdotally know that patients ‘prepared’ for study inclusion, by limiting

exercise and exertion in the weeks prior to inclusion. In this respect, it's good to mention that the visits were scheduled in weeks beforehand. We have added the following sentence to better reflect this:

Lines 575-577:

"*Muscle biopsies from patients and healthy controls were obtained 6 days prior to the CPET, and all participants were instructed to refrain from strenuous activity at least 24 hours prior to the biopsy procedure.* "

-has your group considered triple staining of capillaries and muscle fiber types; see, eg., Bailly M, et al. Two new reliable immunohistochemical methods for simultaneous identification of capillaries, the three types of fibers and basal lamina in human skeletal muscle. Histochem Cell Biol. 2020 Sep;154(3):327-337. doi: 10.1007/s00418-020-01895-5. Epub 2020 Jun 26. PMID: 32591977. It would be extremely interesting to know if capillarization of type 1 versus type 2 fibers were differentially affected by bedrest and the two diseases.

We thank the reviewer for the insightful recommendation. We have not performed this triple staining *per se*, because of the following reason: for staining capillaries using immunohistochemistry, people often use CD31 as the main marker for capillaries. We performed some pilot experiments comparing our lectin staining (not immune-labeled) with the CD31 staining, and found that the CD31 does not stain all capillaries in a section. CD31 is an endothelial cell marker that is mainly dependent on shear stress, and actual flow inside the capillary. As such, while it is technically possible to perform such experiment, we are not sure whether this yields important insights, because the CD31 staining will be partly dependent of shear stress-induced endothelial activation.

While we did not perform the triple stain, we did conduct a fiber type-specific capillary analysis, in which we identified fibers present in both the lectin-stained capillary images and the fluorescent fiber type images from serial sections. We were then able to calculate the number of capillaries for a given fiber type (classed as Type I or Type II). This is now included in the text and Supplemental Figure 11. This was limited by the fibers that could be identified in both the capillary images and fiber type images, thus resulting in lower sample sizes. This analysis has now been included, and reveals that capillarization is equally affected in bed rest and patient groups regardless of fibre type.

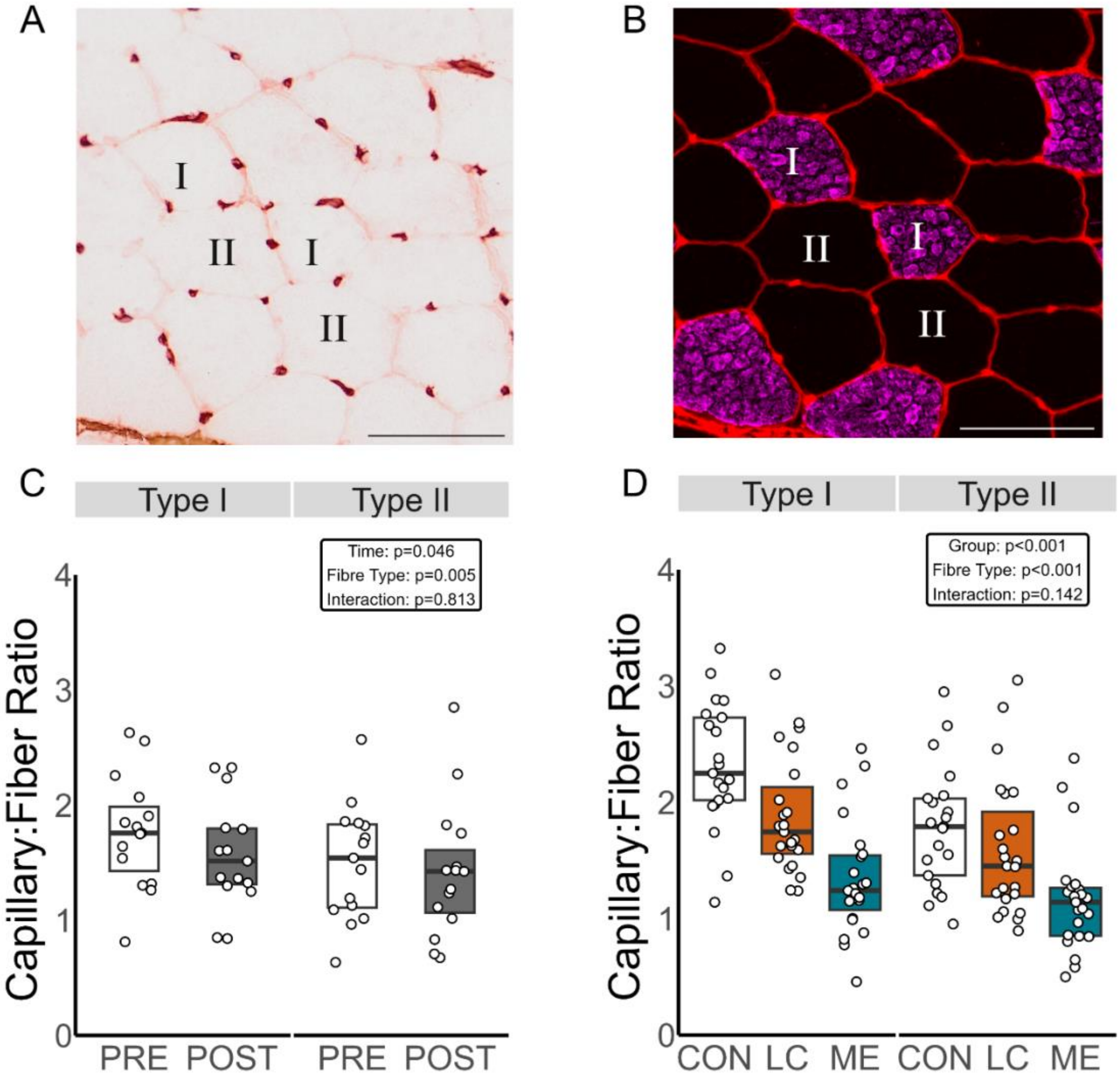

***Supplemental Figure 11**: Fiber type specific capillary-to-fiber ratios were analyzed by overlaying capillary staining (A) with fiber type staining (B) in a subgroup of participants, where possible. Fiber types were classified as type I positive (I) or type I negative (II). Bed rest participants (n=15) exhibited a reduction in capillary-to-fiber ratio for both fiber types ubiquitously (C). Patients with ME/CFS (ME, n=23) had lower capillary-to-fiber ratios in both fiber types compared to both patients with long COVID (LC, n=23) and healthy controls (CON, n=21; D; P=0.0013 and P<0.001 respectively). Patients with long COVID had the tendency (P=0.096) to have lower capillary-to-fiber ratios than healthy controls. In all groups, type I fibers displayed greater capillary-to-fiber ratios compared to type II fibers. After Box-Cox transformations, all data remained non-normally distributed. Data from bed rest participants were compared using a repeated measures 2-way aligned rank transform test. Data from patients with ME/CFS, patients*

with long COVID and healthy controls were compared using a 2-way aligned rank transform test, with a pairwise estimated marginal means post-hoc test for group effects. Scale bars represent 100 μm.

Reviewer #4 (Remarks to the Author):

I co-reviewed this manuscript with one of the reviewers who provided the listed reports. This is part of the Nature Communications initiative to facilitate training in peer review and to provide appropriate recognition for Early Career Researchers who co-review manuscripts.

We thank this reviewer for their help in the review process.

Reviewer #1 (Remarks to the Author)

This paper is much improved in revision. The following points remain to be resolved, however.

We thank the reviewer for the positive feedback. Below we have outlined the response to the remaining points and where we made adjustments in the manuscript.

Line(s) 142 How, specifically, might altered ventilatory patterns lower VO2max? There was no evidence for arterial O2 desaturation during maximal exercise was there?

Thank you. We did not measure arterial O_2 saturation, and thus to avoid suggesting that the altered ventilatory patterns contributed to the reduced $\dot{V}O_{2max}$, we have changed the wording to :

"Whole body exercise results suggest that altered ventilatory patterns occurred concomitantly with the reduction in $\dot{V}O_{2max}$ following bed rest"

143 Isn't an "elevation in VO2-HR slope" indicative of greater O2 extraction (or SV)? Please rewrite for clarity.

Thank you, this was indeed unclear. We have calculated the maximal O_2 pulse as $\dot{V}O_{2max}$/maximal heart rate, and the HR-$\dot{V}O_2$-slope, rather than the $\dot{V}O_2$-HR -slope. The units are beats*L^{-1}. Hence, this is the inverse relationship to the one reported. We apologize for this error. We have adjusted the term $\dot{V}O_2$-HR slope to HR-$\dot{V}O_2$slope throughout. Also, we have added this to the text on line 143-144:

"which are suggestive of a lower stroke volume or peripheral oxygen extraction"

299 As before, please explain how maximal HR increases with bed rest.

Thank you. Our study design was not aimed to study why maximal heart rate increased with bed rest. As such, we can only speculate about the causes of the increase in maximal heart rate. We have added the following sentence to the discussion in lines 299-301.

"*The increase in both resting and maximal heart rate, which has been noted previously (Spaak et al., 2005; Convertino et al., 1990; Kamiya et al., 2003), following bed rest is not fully understood, but may be related to reductions in plasma and stroke volume (Spaak et al., 2005), altered baroreflex function (Convertino et al., 1990), or shifts in the balance between parasympathetic*

and sympathetic nervous system activity (Kamiya et al., 2003) observed after bed rest."

301- As above, increased VO2-HR slope would indicate increased SV or peripheral O2 extractions???

We have added this to the text on line 302:

"our patient groups exhibited an increased HR-VO_2 slope"

341 Less obliquely cut capillaries surely?

Thank you. We have adjusted this to "less".

354 No! Convective O2 delivery i.e., blood flow is controlled at the arteriolar not capillary level in skeletal muscle.

Thank you. We agree that blood flow is indeed controlled at the arteriolar level. Our intention here was not to imply that capillaries control convective O_2 delivery, but rather that a lower capillary density reduces the surface area and spatial distribution for oxygen exchange at the fibre level. We have revised the text to clarify this distinction.

We have adjusted this section to read as:

"*This suggests that fewer capillaries are available per unit fibre area in patients, which is distinct from the capillary alterations following strict bed rest (Hendrickse et al. 2022). This reduces the surface area and spatial distribution for gas exchange at high metabolic rates, and represents a structural limitation to oxygen diffusion, irrespective of upstream regulation of blood flow at the arteriolar level*."

372 The Broxterman et al. paper cited is small muscle mass exercise where the CV limitation/ceiling is removed….a crucial distinction.

We thank the reviewer for the critique and have clarified in the text that the limitation refers to mitochondrial when compared to convective oxygen delivery when the cardiovascular system is not limiting. We have altered the section to:

This situation contrasts with healthy sedentary individuals, where mitochondrial capacity is the primary limitation to $\dot{V}O_{2max}$ in small muscle mass exercise (i.e. under conditions where cardiovascular limitations are minimized) (Broxterman et al., 2024). Although we utilized whole-body exercise in the current study, and the mitochondrial oxidative ceiling is far less

likely to be reached in this mode of exercise, our observation of the significant association between SDH-activity and oxidative phosphorylation and $\dot{V}O_{2max}$ that occurs after bed rest (Figure 3C), and across different populations(Van der Zwaard et al., 2016) is at least consistent with the notion of a role for mitochondria in explaining the variation of $\dot{V}O_{2max}$ in patient populations.

Reviewer #2 (Remarks to the Author):

We would like to thank the reviewer for their positive reception of the changes. We are pleased that the reviewer found our responses adequately addressed their concerns.

***

Required revision i):

The following revisions (adjustments) are required:

i) Clinical case definition: The clinical case definition for ME/CFS needs to be clarified, as the ME/CFS diagnosis was made before 2020. Report the following aspects: type of infection (respiratory tract, EBV, gastroeneritis, inflamed bowel disease, rheumatological aspects, autonomic symptoms, e.g., POTS, dyspnea).

Response from the Reviewer: The requested revisions have been implemented and substantively strengthen the manuscript. Supplemental Figure 1 now presents the onset data for the cohort in detail. As acknowledged in the ME/CFS literature, recall bias regarding precise onset timing is a well-recognized methodological challenge; accordingly, a majority of participants reported an unknown exact onset while nonetheless identifying a distinct point in time at which symptoms emerged. Importantly, this pattern is entirely consistent with — and indeed expected under — the Canadian Consensus Criteria (CCC), thereby confirming that the case definition adheres to the most stringent and widely accepted diagnostic framework in the field. The presented data also demonstrate that this is not an exclusively long COVID cohort.

We are pleased that reviewer finds the changes agreeable.

***

Required revision ii):

ii) For both groups (long COVID and ME/CFS): Report the frequency and severity of symptoms and the DSQ items in a table. This presents an opportunity for the paper, as Q9, for example, allows for differentiation between < 14 hours and > 14 hours (e.g., Kedor et al. 2022, https://doi.org/10.1038/s41467-022-32507-6). The data analysis should be expanded to include, for example, correlative measures (e.g., VO2-heart rate slope).

Response from the Reviewer: The DSQ-PEM data presented in the supplemental figures provide compelling and transparent evidence for the diagnostic validity of the Long COVID and ME/CFS cohort. The finding that all patients with Long COVID and ME/CFS met the threshold for a positive PEM diagnosis via the DSQ-PEM — while no healthy controls did — unambiguously confirms that the cohort fulfills the Canadian Consensus Criteria (CCC) for ME/CFS, with PEM scores consistently exceeding the required threshold of > 14. Moreover, the significantly elevated severity and frequency scores, the more negative responses to DSQ-PEM items 6–8 and 10, as well as the markedly prolonged recovery durations following activity, collectively indicate that the cohort represents severely affected individuals. This characterization of disease severity to be of considerable importance for the interpretation of the findings, as it establishes a well-defined and clinically meaningful patient population and thereby strengthens the generalizability and clinical relevance of the reported results.

We thank the reviewer for the positive commentary.

***

Required revision iii):
iii) Please report the lactate values at all sampling times (e.g., in Supplementary Figure 2).
Response from the Reviewer: For the results of the paper, it is very significant that no significant differences exist for the investigated conditions concerning lactate. This may also be an argument against an effect of decoding alone... .
***

We thank the reviewer for the insight. Whilst we would like to draw more conclusions from this data, the exercise tests were not conducted to analyze lactate accumulation throughout, as was done in Lien *et al*., Physiological Reports, 2019 (doi: 10.14814/phy2.14138). This leaves an opportunity for future research to investigate the lactate accumulation time course in future research.

***

Required revision iv):
iV) The muscle-associated analyses are excellent and show that deconditioning alone cannot explain PEM. This is very valuable and helpful. It is very helpful if you can summarize the pathophysiological changes (respiratory, cardiovascular, muscle physiological) in the discussion with regard to the following aspects: finding, pathophysiological interpretation concerning deconditioning and other explanations (e.g. Haunhorst et al. 2025, PMCID: PMC11825644).
Response from the Reviewer: Thank you for the evidence-based classification that

skeletal muscle mitochondrial dysregulation is associated with PEM (the data of the presented paper). This possibly points to a phenotypic approach.
***

We thank the reviewer for the positive comments.

Reviewer #2 (Remarks on code availability):

Thanks for the README file in the Source Code, there are enough instructions for installing and running!
The R-Code is transparent documented.

Thank you.

Reviewer #3 (Remarks to the Author):

Thank you for the thoughtful responses to my review. All of my questions have been adequately addressed.

We are pleased to hear that the reviewer is satisfied with the changes.

Reviewer #4 (Remarks to the Author):

I co-reviewed this manuscript with one of the reviewers who provided the listed reports. This is part of the Nature Communications initiative to facilitate training in peer review and to provide appropriate recognition for Early Career Researchers who co-review manuscripts.

We thank the reviewer for their time.
