# Supplementary material for: Skeletal muscle properties in long COVID and ME/CFS differ from those induced by bed rest
Source: Nat Commun. 2026 Jul 28;17:9125. doi: 10.1038/s41467-026-75725-y (PMC13518852; doi:10.1038/s41467-026-75725-y)
Supplement: Supplementary file 3 [file 41467_2026_75725_MOESM3_ESM.pdf]

nature portfolio

Corresponding author(s): Dr. Rob Wüst

Last updated by author(s): 01/04/2026

Reporting Summary

Nature Portfolio wishes to improve the reproducibility of the work that we publish. This form provides structure for consistency and transparency in reporting. For further information on Nature Portfolio policies, see our Editorial Policies and the Editorial Policy Checklist.

Statistics

For all statistical analyses, confirm that the following items are present in the figure legend, table legend, main text, or Methods section.

n/a	Confirmed	
☐	☒	The exact sample size (*n*) for each experimental group/condition, given as a discrete number and unit of measurement
☐	☒	A statement on whether measurements were taken from distinct samples or whether the same sample was measured repeatedly
☐	☒	The statistical test(s) used AND whether they are one- or two-sided<br>*Only common tests should be described solely by name; describe more complex techniques in the Methods section.*
☐	☐	A description of all covariates tested
☐	☒	A description of any assumptions or corrections, such as tests of normality and adjustment for multiple comparisons
☐	☒	A full description of the statistical parameters including central tendency (e.g. means) or other basic estimates (e.g. regression coefficient) AND variation (e.g. standard deviation) or associated estimates of uncertainty (e.g. confidence intervals)
☐	☒	For null hypothesis testing, the test statistic (e.g. *F*, *t*, *r*) with confidence intervals, effect sizes, degrees of freedom and *P* value noted<br>*Give P values as exact values whenever suitable.*
☒	☐	For Bayesian analysis, information on the choice of priors and Markov chain Monte Carlo settings
☒	☐	For hierarchical and complex designs, identification of the appropriate level for tests and full reporting of outcomes
☐	☒	Estimates of effect sizes (e.g. Cohen's *d*, Pearson's *r*), indicating how they were calculated

Our web collection on statistics for biologists contains articles on many of the points above.

Software and code

Policy information about availability of computer code

Data collection

Exercise test: Lode Excalibur Sport, Lode, Groningen, The Netherlands or Monark L7TT, Monark Sports & Medical, Vansbro, Sweden
Pulmonary gas exchange: Cosmed Quark CPET; Cosmed, Rome, Italy.
Lactate measurement: Lactate Pro 2 LT-1730, ARKRAY Ltd., United Kingdom.
Step counter: Actigraph wGT3X-BT
Mitochondrial respiration data collection software: DatLab Version 7.4.0.4 (Ororoboros Instruments, Innsbruck, AT)
Imaging: VS200 Research Slide Scanner (Olympus) using Slideview 5.0. for fluorescent images and a DMRB microscope (Leica, Wetzlar) using a CCD camera, calibrated gray filters, and an individual calibration curve at 660 nm for SDH activity and myoglobin.
Clinical data was collected in CASTOR EDC

Data analysis	All data were analyzed using R studio built under R version 4.0.3 (R Core Team 2013, Vienna, Austria). Custom R codes are available online at https://github.com/bcn95/LC_ME_CFS_BED_REST. Custom R code was used to analyze data as described in the "Methods" section of the manuscript. Custom R codes can be found at https://github.com/bcn95/LC_ME_CFS_BED_REST with a README file and source data file.<br><br>Image processing and analysis was conducted using ImageJ (National Institutes of Health, version 1.53u).<br><br>Fiber type composition was performed with ImageJ and Sandia Matlab AnalysiS Hierarchy (SMASH) Toolbox (version 1.0) in Matlab (version 2022a).<br><br>All figures were made using Biorender.

For manuscripts utilizing custom algorithms or software that are central to the research but not yet described in published literature, software must be made available to editors and reviewers. We strongly encourage code deposition in a community repository (e.g. GitHub). See the Nature Portfolio guidelines for submitting code & software for further information.

Data

Policy information about availability of data

All manuscripts must include a data availability statement. This statement should provide the following information, where applicable:

- Accession codes, unique identifiers, or web links for publicly available datasets
- A description of any restrictions on data availability
- For clinical datasets or third party data, please ensure that the statement adheres to our policy

All necessary data to recreate figures and data analysis can be found in the source data file, which can be found at https://github.com/bcn95/LC_ME_CFS_BED_REST (DOI: 10.5281/zenodo.20642303).

Research involving human participants, their data, or biological material

Policy information about studies with human participants or human data. See also policy information about sex, gender (identity/presentation), and sexual orientation and race, ethnicity and racism.

Reporting on sex and gender	Sex is reported for all participants. Sex of participants was determined based on self-report. Patients were enrolled based on having long COVID or ME/CFS. All long COVID and ME/CFS were age and sex matched with the control group. There was no sex or gender analysis. Ethical approval was obtained by Medical Ethics Committee of the Amsterdam UMC (NL78394.018.21) for the long COVID and ME/CFS cohort and the Medical Association North Rhine (number 2018143) and NASA (Johnson Space Center, Houston, United States) for the AGBRESA cohort.
Reporting on race, ethnicity, or other socially relevant groupings	There was no reporting on race, ethnicity or other socially relevant grouping.
Population characteristics	Long COVID and ME/CFS cohort:<br>Patients with long COVID were diagnosed by two experienced clinicians for long COVID symptomology and exclusion of potential differential diagnoses. All long COVID patients were diagnosed with post-exertional malaise (PEM) by the DSQ-PEM74, had a minimum period of long COVID-related symptoms of six months, and were between 18 and 65 years old. No symptoms were present before the confirmed diagnosis of SARS-CoV-2. None of the included participants were admitted to the hospital during acute SARS-CoV-2 infection and were healthy prior to NAAT or serology-proven SARS-CoV-2 infection. Reported symptom durations were from symptom onset to study enrolment (February 2022 for long COVID and October 2023 for ME/CFS). Exclusion criteria were a medical history of cardiovascular/pulmonary disease, diabetes mellitus, concurrent treatment with metabolism or coagulant-altering drugs during the study period (statins, corticosteroids, SGLT2 inhibitors, GLP1 receptor agonists, platelet aggregation blockers and any anticoagulants), adiposity and body mass index >35 (related to muscle biopsy obtainment), pregnancy, active infection, severe renal dysfunction or any other prior chronic illness or >6 alcohol units per day or >14 alcohol units per week. Smoking was not an exclusion criterion. One long COVID patient was excluded due to a recent SARS-CoV-2 re-infection (<7 days).<br>Patients with ME/CFS all fulfilled the Canadian Consensus Criteria (CCC), exhibited PEM according to the DSQ-PEM and consulted with a ME/CFS specialist or post-COVID physician, and were between 18 and 65 years old. ME/CFS diagnosis was prior to 2020, and all patients had a confirmed previous diagnosis of SARS-CoV-2. Exclusion criteria were identical to those of the patients with long COVID.<br>None of the healthy controls had residual symptoms after the SARS-CoV-2 infection and none were hospitalized within 6 months of study participation. Healthy controls withdrew because of the invasive nature of the protocol (n=2), symptoms related to a burn-out (n=1), and a novel diagnosis of uncontrolled hypertension (n=1).<br><br>AGBRESA cohort:<br>24 healthy participants underwent a 60-day strict 6 degrees head-down tilt bed rest (8 Females/16 Males, 27-38 years old). Participants followed standardized diets, consuming 1.6 times their resting metabolic rate before bed rest and 1.3 times their resting metabolic rate during bed rest. Fluid intake was controlled, and ingestion of caffeine and alcohol were prohibited.
Recruitment	Long COVID and ME/CFS cohort:<br>Long COVID and ME/CFS patients and healthy controls were recruited from the Amsterdam UMC post-covid clinic. Long COVID patients were diagnosed by two experienced clinicians for long COVID symptomology and exclusion of potential

differential diagnoses. Patients with ME/CFS all fulfilled the Canadian Consensus Criteria (CCC), exhibited PEM according to the DSQ-PEM and consulted with a ME/CFS specialist or post-COVID physician, and were between 18 and 65 years old. ME/CFS diagnosis was prior to 2020, and all patients had a confirmed previous diagnosis of SARS-CoV-2. Exclusion criteria were identical to those of the patients with long COVID.
Our inclusion from the post-covid clinic may have let to a more severe ill population as they are the ones to seek assistance of healthcare professionals.

AGBRESA:

Ethics oversight

Long COVID and ME/CFS cohort: Medical Ethics Committee of the Amsterdam UMC (NL78394.018.21)

AGBRESA cohort: Medical Association North Rhine (number 2018143) and NASA (Johnson Space Center, Houston, United States)

Note that full information on the approval of the study protocol must also be provided in the manuscript.

Field-specific reporting

Please select the one below that is the best fit for your research. If you are not sure, read the appropriate sections before making your selection.

☒ Life sciences ☐ Behavioural & social sciences ☐ Ecological, evolutionary & environmental sciences

For a reference copy of the document with all sections, see nature.com/documents/nr-reporting-summary-flat.pdf

Life sciences study design

All studies must disclose on these points even when the disclosure is negative.

Sample size

Long COVID and ME/CFS cohort:
A reduction in exercise capacity is a prerequisite for the identification of post-exertional fatigue. As the reported time-course of reductions in exercise capacity with post-exertional malaise syndrome is highly similar, the data of Snell et al. (2013) can be utilized to determine the required sample size for this project. Snell et al. (2013) reported a large effect size for differences in exercise capacity between groups (Cohen's d = 1.1). Hence, to assess for the hypothesized effect size of 1.1 with a two-tailed alpha level of 0.05 and a β of 0.95, with a case-control ratio of 1, a group size of 19 would be required.
We anticipate that the heterogeneity of our patient population is larger, and that not all patients will undergo all testing. Therefore, we conservatively assess that a group size of 25 will provide enough statistical and clinical power.

AGBRESA cohort:
The sample size of 24 (8 subjects in each of 3 arms) was pre-determined by NASA and ESA. Based on the selected sample size, power analyses were an intrinsic part of the peer-review process and performed by a team of statisticians. The hypothesis was that an increase in RNFL thickness (change from baseline to after intervention) will be smaller with daily antigravity exposure (intervention 1 and 2). With 8 subjects per group our power would be 80% to detect a difference of 5.5 µm or greater in mean thickness values between intervention and control subjects using a two-sided test, with alpha=0.05. This calculation indicates that with sample size of 8 per group, our power is adequate to detect a difference between groups if the intervention 1 or 2 attenuate the increase in RNFL thickness by 50% (or more) compared to the HDT alone (4.5 to 6 µm / 10 to 11 µm).

Data exclusions

Due to software issues, three patients with ME/CFS undertook ramp rates that were too high, resulting in a duration of exercise that was not sufficient to establish acute exercise responses. Evaluation of maximal exercise was still possible in these participants, thus submaximal values were excluded.

For respiration experiments, a membrane integrity measure above 1.15 was deemed insufficient, and those data points were excluded from respiration related analyses (n=14 of 129 measures), as described in the methods.

Replication

None of the findings were reproduced due to the invasive nature of the biopsy studies, and the length of the bed rest study.

Randomization

Long COVID and ME/CFS cohort: No randomization. All participants were age and sex matched, and followed the same study procedure.

AGBRESA cohort: Participants were randomized to one of the three treatment arms during the head-down tilt bed rest: Arm 1: Intervention Group 1: 6° HDBR with supine centrifugation at +1Gz at the center of mass for 30 minutes continuous per day; Arm 2: Intervention group 2: 6° HDBR with supine centrifugation at +1Gz at the center of mass for 6 bouts of 5 minutes per day (sessions 2-6 are separated by 5 minutes of rest); Arm 3: control Group: 6° HDBR with no centrifugation (passive control). All participants enrolled in the AGBRESA study underwent 55 days of bed rest. The primary outcomes of the original study (published as Laurie et al.,2021. Optic disc edema and chorioretinal folds develop during strict 6° head-down tilt bed rest with or without artificial gravity. Physiological reports) found no differences between arms, and therefore the treatment was deemed unsuccessful. For the purposes of the current manuscript, there was therefore no distinction between arms.

Blinding

Investigators were not blinded for either study cohort, as both were observational studies.

Reporting for specific materials, systems and methods

We require information from authors about some types of materials, experimental systems and methods used in many studies. Here, indicate whether each material, system or method listed is relevant to your study. If you are not sure if a list item applies to your research, read the appropriate section before selecting a response.

Materials & experimental systems

n/a	Involved in the study	
☐	☒	Antibodies
☒	☐	Eukaryotic cell lines
☒	☐	Palaeontology and archaeology
☒	☐	Animals and other organisms
☐	☒	Clinical data
☒	☐	Dual use research of concern
☒	☐	Plants

Methods

n/a	Involved in the study	
☒	☐	ChIP-seq
☒	☐	Flow cytometry
☒	☐	MRI-based neuroimaging

Antibodies

Antibodies used

See Table 2 for full details.
BA-D5, 1µg/mL, DSHB; DSHB Cat# BA-D5, RRID:AB_2235587
6H1, 5µg/mL, DSHB; DSHB Cat# 6H1, RRID:AB_1157897
SC-71 1µg/mL, DSHB; DSHB Cat# SC-71, RRID:AB_2147165
WGA 350, 1:25, Invitrogen; W11263
Ulex europaeus agglutinin I, 20µg/mL , Vector Laboratories; Vector Laboratories Cat# B-1065, RRID:AB_2336766
Goat Anti-mouse IgG2B 555, 1:2000, Invitrogen; Thermo Fisher Scientific Cat# A-21147, RRID:AB_2535783
Goat Anti-mouse IgM 488, 1:2000, Invitrogen; Thermo Fisher Scientific Cat# A-21042, RRID:AB_2535711
Goat Anti-mouse IgG1 647,1:2000, Invitrogen; Thermo Fisher Scientific Cat# A-21240, RRID:AB_2535809
Vectastain Elite ABC Kit PK-6100, Vector Laboratories; Vector Laboratories Cat# PK-6100, RRID:AB_2336819
ImmPACT™ AMEC Red Peroxidase Substrate, Vector Laboratorie; Vector Laboratories Cat# SK-4285, RRID:AB_2336519

Validation

All antibodies are commercially available and validated by the vendors. No additional validation was performed. Listed below are relevant citations. Full data can be found in Table 2.

Muscle fiber type
BA-D5 (MHC-I), 1ug/ml, DSHB, (DSHB Cat# BA-D5, RRID:AB_2235587), confirmed species reactivity for humans, Schiaffino S, Gorza L, Sartore S, Saggin L, Ausoni S, Vianello M, Gundersen K, Lømo T. Three myosin heavy chain isoforms in type 2 skeletal muscle fibres. J Muscle Res Cell Motil. 1989 Jun;10(3):197-205. doi: 10.1007/BF01739810. PMID: 2547831.
SC-71 (MHC-IIA), 1ug/ml, DSHB, (DSHB Cat# SC-71, RRID:AB_2147165) ;confirmed species reactivity for humans, Schiaffino S, Gorza L, Sartore S, Saggin L, Ausoni S, Vianello M, Gundersen K, Lømo T. Three myosin heavy chain isoforms in type 2 skeletal muscle fibres. J Muscle Res Cell Motil. 1989 Jun;10(3):197-205. doi: 10.1007/BF01739810. PMID: 2547831.
6H1 (MHC-IIX) 5ug/ml, DSHB (60 min), (DSHB Cat# 6H1, RRID:AB_1157897), confirmed species reactivity for humans,
Goat-anti mouse IgG2b 555, 1:1000, Invitrogen A21147; (Thermo Fisher Scientific Cat# A-21147, RRID:AB_2535783)
Goat anti-mouse IgG1 647, 1:1000, Invitrogen A21240; (Thermo Fisher Scientific Cat# A-21240, RRID:AB_2535809)
Goat anti-mouse IgM 488, 1:1000, Invitrogen A21042; (Thermo Fisher Scientific Cat# A-21042, RRID:AB_2535711)

Endothelial cells
Biotinylated UEA-1, 1:100, B-1065, Vector Laboratories, (Vector Laboratories Cat# B-1065, RRID:AB_2336766)
Vectastain Elite ABC Kit PK-6100, Vector Laboratories, (Vector Laboratories Cat# PK-6100, RRID:AB_2336819)
ImmPACT™ AMEC Red Peroxidase Substrate SK-4285, Vector Laboratories; (Vector Laboratories Cat# SK-4285, RRID:AB_2336519)

Clinical data

Policy information about clinical studies
All manuscripts should comply with the ICMJE guidelines for publication of clinical research and a completed CONSORT checklist must be included with all submissions.

Clinical trial registration

Long COVID and ME/CFS cohort: NCT05225688
AGBRESA cohort: DRKS00015677

Study protocol

Long COVID and ME/CFS cohort: https://clinicaltrials.gov/study/NCT05225688
AGBRESA cohort: https://drks.de/search/en/trial/DRKS00015677

Data collection	Long COVID and ME/CFS cohort:<br>Post-COVID-19 clinic of the Amsterdam University Medical Centers (UMC) and the Faculty of Behavioral and Movement Sciences (Vrije Universiteit Amsterdam). Clinical data was stored in CASTOR EDC. All patients were recruited between 2022 and 2024.<br><br>AGBRESA cohort:<br>German Aerospace Agency (Other Deutsches Zentrum für Luft- und Raumfahrt, Institut für Luft- und Raumfahrtmedizin Köln; recruitment dates: 2019-03-25 -2021-12-08)
Outcomes	Full outcomes can be found on https://clinicaltrials.gov/study/NCT05225688 and https://drks.de/search/en/trial/DRKS00015677<br><br>Long COVID and ME/CFS cohort:<br>The primary outcomes of the long COVID and ME/CFS cohort was whole-body aerobic exercise capacity, mitochondrial respiration, succinate dehydrogenase activity, fiber type composition, and capillarization.<br>Mitochondrial respiration data collection software: DatLab Version 7.4.0.4 (Ororoboros Instruments, Innsbruck, AT)<br>Maximal aerobic exercise capacity: Cycling load: Lode Excalibur Sport, Lode, Groningen, The Netherlands or Monark L7TT, Monark Sports & Medical, Vansbro, Sweden; Pulmonary gas exchange: Cosmed Quark CPET; Cosmed, Rome, Italy.<br>Succinate dehydrogenase activity, fiber type composition, and capillarization imaging: VS200 Research Slide Scanner (Olympus) using Slideview 5.0. for fluorescent images and a DMRB microscope (Leica, Wetzlar) using a CCD camera, calibrated gray filters, and an individual calibration curve at 660nm for SDH activity.<br>Succinate dehydrogenase activity and capillarization analsysis: Image processing and analysis was conducted using ImageJ (National Institutes of Health, version 1.53u and later).<br>Fiber type composition analysis was performed with ImageJ and Sandia Matlab AnalysiS Hierarchy (SMASH) Toolbox (version 1.0) in Matlab (version 2022a).<br><br>AGBRESA cohort:<br>The primary outcome measurement of AGBRESA was an increase in peripapillary retinal nerve fiber layer thickness (RNFL), and published as Laurie et al.,2021. Optic disc edema and chorioretinal folds develop during strict 6° head-down tilt bed rest with or without artificial gravity. Physiological reports.<br>Mitochondrial respiration data collection software: DatLab Version 7.4.0.4 (Ororoboros Instruments, Innsbruck, AT)<br>Maximal aerobic exercise capacity: Cycling load: Lode Excalibur Sport, Lode, Groningen, The Netherlands; pulmonary gas exchange: Innocor, Innovision, Odense, Denmark)

Plants

Seed stocks	*Report on the source of all seed stocks or other plant material used. If applicable, state the seed stock centre and catalogue number. If plant specimens were collected from the field, describe the collection location, date and sampling procedures.*
Novel plant genotypes	*Describe the methods by which all novel plant genotypes were produced. This includes those generated by transgenic approaches, gene editing, chemical/radiation-based mutagenesis and hybridization. For transgenic lines, describe the transformation method, the number of independent lines analyzed and the generation upon which experiments were performed. For gene-edited lines, describe the editor used, the endogenous sequence targeted for editing, the targeting guide RNA sequence (if applicable) and how the editor was applied.*
Authentication	*Describe any authentication procedures for each seed stock used or novel genotype generated. Describe any experiments used to assess the effect of a mutation and, where applicable, how potential secondary effects (e.g. second site T-DNA insertions, mosiacism, off-target gene editing) were examined.*
